# Genome-wide CRISPR-KO Screen Uncovers mTORC1-Mediated Gsk3 Regulation in Naive Pluripotency Maintenance and Dissolution

**DOI:** 10.1016/j.celrep.2018.06.027

**Published:** 2018-07-11

**Authors:** Meng Li, Jason S.L. Yu, Katarzyna Tilgner, Swee Hoe Ong, Hiroko Koike-Yusa, Kosuke Yusa

**Affiliations:** 1 Wellcome Sanger Institute, Hinxton, Cambridge CB10 1SA, UK

**Keywords:** CRISPR, screening, naive pluripotency, exit from pluripotency, Akt, mTORC1, mTORC2, GATOR1, Nprl2, Tsc2

## Abstract

The genetic basis of naive pluripotency maintenance and loss is a central question in embryonic stem cell biology. Here, we deploy CRISPR-knockout-based screens in mouse embryonic stem cells to interrogate this question through a genome-wide, non-biased approach using the Rex1GFP reporter as a phenotypic readout. This highly sensitive and efficient method identified genes in diverse biological processes and pathways. We uncovered a key role for negative regulators of mTORC1 in maintenance and exit from naive pluripotency and provided an integrated account of how mTORC1 activity influences naive pluripotency through Gsk3. Our study therefore reinforces Gsk3 as the central node and provides a comprehensive, data-rich resource that will improve our understanding of mechanisms regulating pluripotency and stimulate avenues for further mechanistic studies.

## Highlights

• Genome-wide CRISPR screening identifies naive pluripotency regulators in mouse ESCs
• mTORC1-negative regulators from two axes show opposing phenotypes
• Gator1 is required for proper self-renewal and differentiation via Gsk3 regulation
• Tsc2 loss causes Akt-dependent, mTORC1-dependent Gsk3 suppression

## Graphical Abstract

**Figure:**
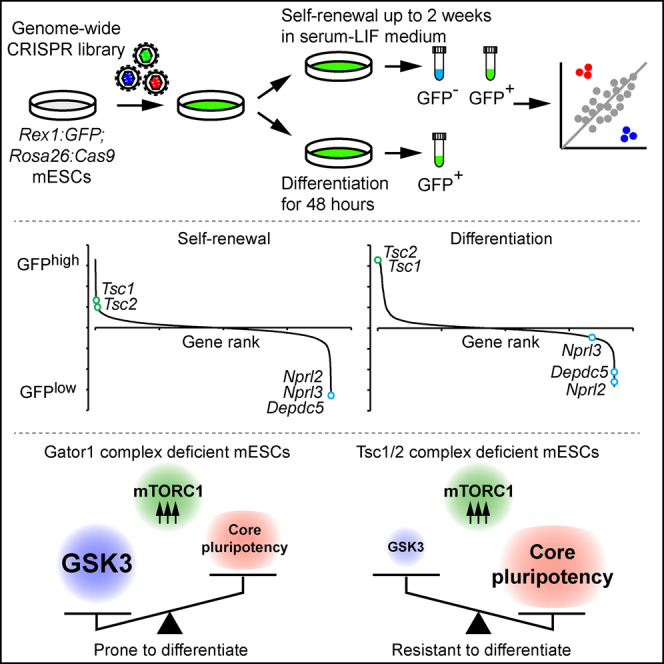

## Teaser

Li et al. conducted genome-wide CRISPR screens in mouse ESCs to identify genes affecting maintenance of and exit from naive pluripotency using a Rex1GFP reporter. They show that loss of two mTORC1-negative regulators, Tsc1/2 and Gator1, can cause opposing phenotypes through differential regulation of Gsk3 activity.

## Introduction

Mouse embryonic stem cells (mESCs) are derived from the inner cell mass of blastocyst-stage embryos and can be indefinitely propagated while maintaining the ability to differentiate into all three germ layers. They have served not only as a platform for genome manipulation and production of transgenic mice but also as an essential model system to study the molecular mechanisms of self-renewal and differentiation. In particular, mechanisms that underpin the maintenance of pluripotency have been the subject of intense research, establishing the framework through which the pluripotent state is regulated by intrinsic and extrinsic factors. Intrinsically, the core transcription factors Pou5f1, Sox2, and Nanog act together with accessory factors Esrrb, Klf2, and Tfcp2l1 to consolidate the pluripotent identity (Hackett and Surani, 2014). Extrinsically, leukemia inhibitory factor (LIF)-STAT3 signaling plays a key role to sustain pluripotency (Ohtsuka et al., 2015) and Wnt signaling cooperates to suppress differentiation (Sato et al., 2004), whereas FGF-MAPK signaling is essential for mESCs to initiate differentiation (Kunath et al., 2007). In uncovering these basic principles, culture conditions that permit the preservation of pluripotency via dual inhibition of MEK and Gsk3 kinases (termed 2i) were established (Ying et al., 2008). mESCs cultured with 2i closely resemble epiblasts in pre-implantation embryos, sharing transcriptomic and epigenomic features that reflect the ground or naive state of pluripotency (Leitch et al., 2013, Marks et al., 2012).

As the mechanisms of pluripotency maintenance have become clearer, research focus has shifted toward understanding how the exit from pluripotency and initiation of lineage specification are achieved. In response to differentiation cues, mESCs must resolve the naive pluripotency network and initiate transcriptional events that drive the progression through an intermediate or formative state to the primed state (Smith, 2017). One key factor regulating this process is Tcf7l1, which is a transcriptional suppressor and colocalizes with Pou5l1 and Sox2, thereby counteracting their transcriptional activation and suppressing the intrinsic pluripotency program (Cole et al., 2008). Conversely, loss of Tcf7l1 resulted in upregulation of Nanog, severely delaying the onset of differentiation (Pereira et al., 2006). The activity of Tcf7l1 is subject to regulation by Wnt signaling and thus depends on Gsk3 activity. Inhibition of Gsk3 results in the nuclear translocation of β-catenin, which upon binding to Tcf7l1, abrogates its suppressor activity (Wray et al., 2011, Yi et al., 2011). This is a clear example of how extrinsic signaling dictates the dissolution of the core pluripotency network. Although reverse and forward genetic approaches have been successful in identifying such factors (Betschinger et al., 2013, Guo et al., 2011, Kaji et al., 2006, Leeb et al., 2014, Pereira et al., 2006), the full repertoire of genes and pathways involved in this process remains elusive.

The CRISPR-Cas system is the defense machinery found in a range of bacterial and archaea species (Makarova et al., 2015). Among them, the CRISPR-Cas9 system derived from *Streptococcus pyogenes* is most extensively characterized (Jinek et al., 2012, Jinek et al., 2014, Nishimasu et al., 2014, Sternberg et al., 2014) and has been adapted into versatile genetic tools (Adli, 2018). The key advantage of the CRISPR-Cas9 system is the high consistency and efficiency in generating targeted gene knockouts, which has enabled us and others to carry out genome-scale CRISPR-knockout (KO) screening in mammalian cells (Koike-Yusa et al., 2014, Shalem et al., 2014, Wang et al., 2014). CRISPR-KO screening has shown superior detection sensitivity compared to RNAi screens (Evers et al., 2016), and its resolving power is evident in the unraveling of genetic dependencies in cancer cells (Hart et al., 2015, Tzelepis et al., 2016, Wang et al., 2017).

Here we performed CRISPR-KO phenotypic screens to gain more in-depth insight and comprehensive understanding of the maintenance of and exit from naive pluripotency. The unbiased nature of CRISPR-KO screening revealed multiple genes and protein complexes whose functions have not previously been associated with pluripotency maintenance and/or differentiation. In particular, our screen revealed that regulation of Gsk3 activity is a key requirement in initiating differentiation. In addition, regulation of Gsk3 is mediated by Akt/mTOR signaling, subsequently linking nutrient and energy metabolism pathways to the exit from naive pluripotency. Our study therefore represents the most comprehensive account of the factors involved in the regulation of naive pluripotency, providing a key resource for further experimental interrogation.

## Results

### CRISPR-KO Self-Renewal Screen Identifies Genes Regulating Naive Pluripotency

We previously performed a cell-essentiality screen in JM8 mESCs and identified 1,680 genes as essential for survival and proliferation (Tzelepis et al., 2016). However, because the phenotypic readout was proliferation, we could not distinguish factors that positively or negatively affect pluripotency maintenance from those affecting cell survival and/or proliferation. In this study, we therefore redesigned a screen using a Rex1GFP reporter (Wray et al., 2011) as a phenotypic readout. *Rex1* (also known as *Zfp42*) expression is strictly restricted to the naive pluripotent state, and its pattern reflects a heterogeneity typically observed in mESCs cultured in the serum + LIF (SL) condition (Chambers et al., 2007). Upon differentiation, *Rex1* is rapidly downregulated, allowing the near real-time readout of the pluripotent state. However, caution is required when interpreting Rex1GFP phenotype, because non-related mechanisms can influence the GFP expression level. Because genes required for cell survival and/or proliferation in the 2i + LIF (2iL) condition have not been investigated and may differ from those required in the SL condition, we sought to perform a cell-essentiality screen in 2iL in parallel. We generated Rex1GFP-Cas9 mESCs by introducing the *Cas9* gene into the *Rosa26* locus (Figure S1) and used this line as wild-type mESCs throughout this study.

**Figure 1:**
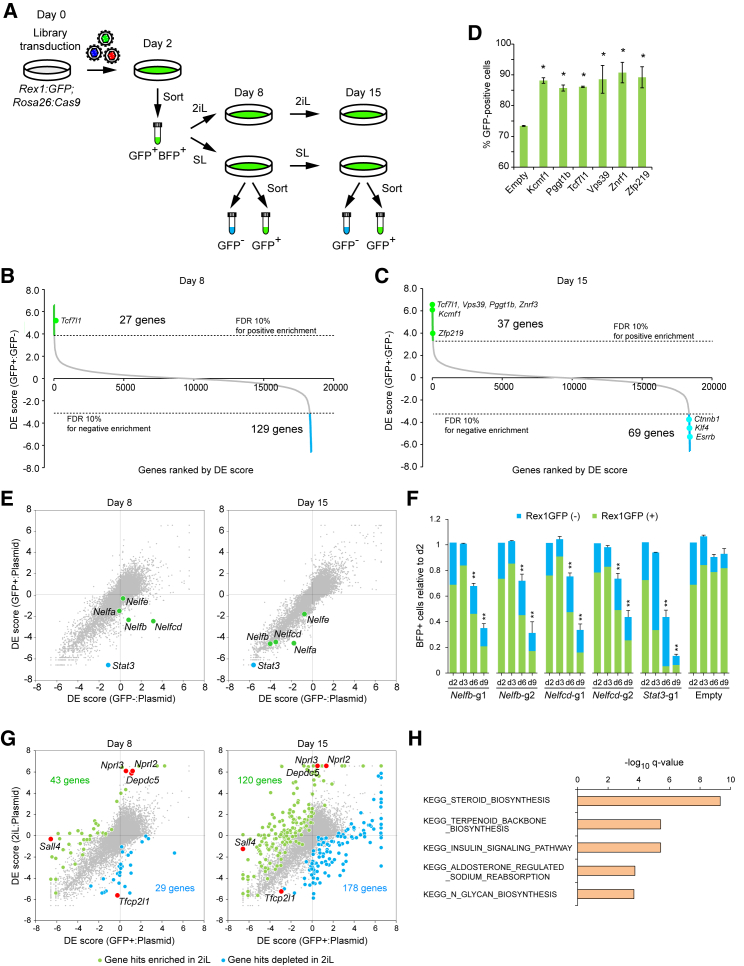
CRISPR-KO Screen in Maintenance of Naive Pluripotency (A) Screening strategy for maintenance of naive pluripotency. Lentivirus used expresses blue fluorescent protein (BFP), and transduced cells were thus enriched on day 2 by sorting. For mESCs in SL, gRNA abundance in sorted GFP+ and GFP− populations was analyzed. (B and C) Screen summaries as ranked DE score plots for day 8 (B) and day 15 (C) by comparing GFP+ and GFP− populations. (D) Validation of newly identified genes. (E and F) Differentiation trajectory (Figure S2) identified potential involvement of the negative elongation factor in naive pluripotency maintenance. (E) Validation experiment was performed with 2 gRNAs each for *Nelfb* and *Nelfcd*, together with a gRNA targeting *Stat3* as a positive control (F). (G) Comparison of the screen results between GFP+ cells in SL and the cells in 2iL. Green and blue dots indicate genes enriched or depleted in cells in 2iL. (H) GO terms overrepresented in processes specifically required in mESCs cultured in 2iL. Data are shown as mean ± SD. (D and F) n = 3. Student’s t test was performed. ^∗^p ≤ 0.05. See also Figures S1 and S2.

Figure 1A outlines our screening strategy. Rex1GFP-Cas9 mESCs were mutagenized with the v2 mouse genome-wide guide RNA (gRNA) library (Tzelepis et al., 2016). On day 2, transduced cells were collected by cell sorting and cultured in either the 2iL or the SL condition. On days 8 and 15 post-transduction, the GFP+ and GFP− populations were collected by sorting for the cells in SL, whereas cells in 2iL were simply collected without sorting. Subsequently, gRNA abundance was analyzed and statistical analyses were performed as detailed later. In all analyses, we computed depletion-enrichment (DE) scores to show contiguous negative-to-positive statistical values (see Experimental Procedures) (Tables S1 and S2).

First, we performed statistical analysis comparing GFP+ and GFP− fractions from the SL condition. This identifies genes that affect the ratio between GFP+ and GFP− populations and thus are most likely to affect self-renewal. From positive selection, we identified 27 and 37 genes whose KO increased the GFP+ fraction on days 8 and 15, respectively, at a cutoff of the false discovery rate (FDR) of 10% (Figures 1B and 1C). Consistent with its established function, *Tcf7l1* was identified as a gene restricting the GFP+ fraction at both time points. We validated 5 genes identified in the day 15 dataset (*Vps39*, *Pggt1b*, *Znrf3*, *Kcmf1*, and *Zfp219*) that have not been previously linked to pluripotency maintenance (Figure 1D). We also identified 129 and 69 genes that, when knocked out, decreased the GFP+ fraction on days 8 and 15, respectively, from negative selection (Figures 1B and 1C). The genes on day 15 included accessory factors such as *Ctnnb1*, *Klf4*, and *Esrrb*.

We then performed additional statistical analysis by comparing the read counts between the library plasmid and the GFP+ or GFP− fractions. This comparison identifies genes that affect mutant representation during the course of screen (i.e., cell survival and/or proliferation). By comparing resulting DE scores between GFP+ and GFP− populations, the kinetics of genes affecting naive pluripotency maintenance can be captured (Figure S2). For instance, genes that exhibit rapid loss of pluripotency upon KO, such as *Pou5f1* and *Sox2*, had been already depleted from both GFP+ and GFP− populations by day 8, but genes in the LIF-Stat3 pathway showed depletion initially from the GFP+ population and then from the entire population, permitting direct observation of the differentiation trajectory (Figures S2G and S2H). We identified two subunits (Nelfb and Nelfcd) of the negative elongation factor complex, showing a trajectory similar to the LIF-Stat3 pathway genes (Figure 1E). Through individual gRNA experiments, we confirmed that gene inactivation resulted in gradual loss of naive pluripotency and eventual depletion from the entire culture (Figure 1F), confirming that our data can accurately capture the loss of the naive state across time.

Lastly, we compared genes essential for survival and/or proliferation between the GFP+ cells in SL and the cells in 2iL and found considerably different requirements to maintain proliferation in these conditions (Figure 1G). For example, *Sall4* is required for self-renewal in the SL condition, but not in the 2iL condition. Loss of *Tfcp2l1* and Gator1 complex genes (*Nprl2*, *Nprl3*, and *Depdc5*) showed a more pronounced effect in the 2iL condition than in the SL condition. Genes specifically required for cells in 2iL were enriched in metabolic and biosynthesis processes, which are likely to be a response to absence of serum constituents, and insulin signaling (Figure 1H). Altogether, these data uncover several genes not previously connected to naive pluripotency maintenance, highlighting the value of our genome-wide loss-of-function screens.

### CRISPR-KO Differentiation Screen Identifies Genes that Impede or Accelerate Pluripotency Exit

**Figure 2:**
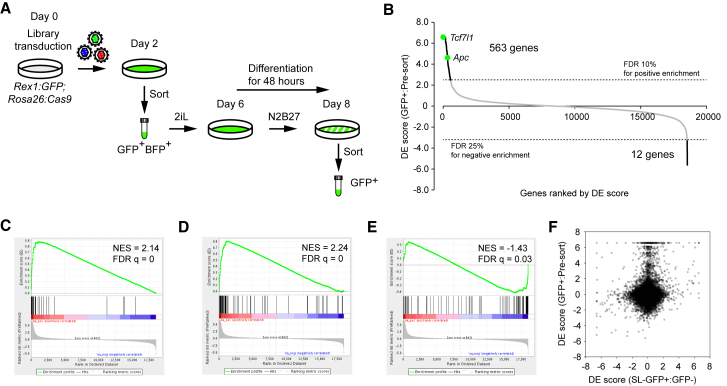
CRISPR-KO Screen in Exit from Naive Pluripotency (A) Screening strategy for exit from naive pluripotency. (B) Screen summary shown as a ranked DE score plot. (C–E) GSEA for a gene set identified by a siRNA screen (Betschinger et al., 2013) (C) and a set of genes identified in positive (D) and negative (E) selection from our self-renewal screen (GFP+:GFP−) on day 15. NES, normalized enrichment score. (F) Comparison of DE scores between self-renewal (day 15) and differentiation screens. Although there are correlations as observed in (D) and (E), most genes identified in exit from pluripotency do not have a major impact on Rex1GFP heterogeneity in maintenance culture. See also Figures S1 and S3.

Next, we performed a CRISPR-KO screen to identify genes required for proper initiation of differentiation using the Rex1GFP reporter (Figure 2A; Figure S3). We compared gRNA abundance of the GFP+ fraction to that of the unsorted population and identified 563 genes (FDR 10% cutoff for positive selection) required for proper exit from pluripotency (Figure 2B) (Tables S1 and S2). The two positive control genes, *Tcf7l1* and *Apc*, were identified among the hits. We were also able to identify 12 genes whose loss accelerated differentiation with a relaxed cutoff of FDR of 25% (Figure 2B). To confirm the validity of our result, we performed gene set enrichment analysis (GSEA) using a control gene set including 28 genes identified by an RNAi screen performed in a similar experimental setting (Betschinger et al., 2013). This gene set showed strong enrichment in our screen, indicating high concordance (Figure 2C). We also performed GSEA using genes identified in our self-renewal screen (Figure 1C) and observed a positive correlation with the differentiation screen; genes that increased the GFP+ fraction in the SL condition showed higher retention of Rex1GFP expression during differentiation, and vice versa (Figures 2D and 2E). This indicates that gene hits in self-renewal in the SL condition most likely show the same effect in the differentiation condition. However, genome-wide comparison of the self-renewal and differentiation screens revealed that most genes showing higher GFP retention upon differentiation did not influence the GFP+:GFP− ratio in the SL condition (Figure 2F). These results suggest that there are at least 2 distinct classes of genes regulating the maintenance of and/or exit from naive pluripotency.

### Pathways Involved in the Exit from Pluripotency Are Diverse

To gain a comprehensive picture of genes involved in naive pluripotency exit, we performed GSEA using the entire Reactome and KEGG gene sets and identified known signaling pathways such as fibroblast growth factor (FGF)-mitogen-activated protein kinase (MAPK), Wnt, and phosphatidylinositol 3-kinase (PI3K) pathways, mRNA degradation, and microRNA (miRNA) biogenesis pathways (Table S3). The remaining processes are relatively less studied in the context of mESC differentiation. For example, mitochondrial genes showed the strongest enrichment; nearly a half of the 563 gene hits were mitochondrial genes. In addition, glycolysis was identified in GSEA. Although it is known that naive pluripotent cells show higher mitochondrial activity than cells with primed pluripotency (Zhou et al., 2012), how ATP production affects the onset of differentiation remains elusive. In addition, genes involved in endosome and vesicle trafficking were enriched, but their involvement is not well understood.

**Figure 3:**
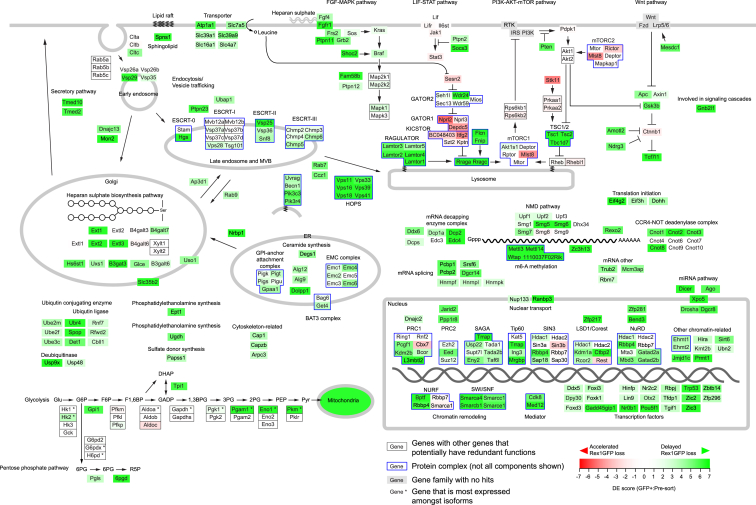
Genes Identified in the CRISPR-KO Screen for Exit from Naive Pluripotency Genes with known functions are placed in pathways, protein complexes, or cellular compartments. When genes with redundant function are present, these genes are boxed in black. Defined protein complexes are boxed in blue. Not all components are shown for protein complexes. See also Figure S4.

We have summarized our findings by grouping our gene hits in the context of signaling, protein complexes, and other functional categories (Figure 3). In the signaling category, genes involved in FGF-MAPK, LIF-STAT, PI3K-AKT, and Wnt pathways were identified. mRNA degradation pathways such as non-sense-mediated decay (Li et al., 2015) and the m^6^-A RNA methylation enzyme complex (Batista et al., 2014, Geula et al., 2015) have been previously described. miRNAs are also known to regulate differentiation (Kanellopoulou et al., 2005, Sinkkonen et al., 2008). In the nucleus, several chromatin-modifying and chromatin-remodeling complexes were identified (Cruz-Molina et al., 2017, Kaji et al., 2006, Whyte et al., 2012). *Pou5f1* was identified as a gene required for differentiation. Although complete loss of Pou5f1 leads to differentiation (Niwa et al., 2000), Pou5f1 is also known to have roles in lineage specification (Wang et al., 2012, Yang et al., 2014). It has also been shown that *Pou5f1*^+/−^ ESCs show enhanced self-renewal capability and resistance to differentiation (Karwacki-Neisius et al., 2013). Because double-stranded break (DSB)-mediated genome editing generates various alleles, our mutant library must have contained heterozygously edited cells, which correspondingly showed a delayed differentiation phenotype.

In addition to known genes and pathways, we identified other pathways not previously connected to naive pluripotency exit. For instance, genes in the heparan sulfate biosynthesis pathway were identified. As a known positive regulator of FGF signaling (Ornitz, 2000), the deficiency likely results in weakened FGF-MAPK signaling. We also identified multiple genes involved in vesicle trafficking and endocytosis. Of the various complexes identified, all 6 genes that compose the homotypic fusion and protein sorting (HOPS) complex showed strong differentiation defects, and one of the genes, *Vps39*, was identified in the self-renewal screen as a factor that decreases heterogeneity (Figures 1C and 1D). We validated some factors by individual gRNA experiments (Figure S4), showing the accuracy of our screen results. Altogether, these findings indicate that our screen both confirmed and added to genes known to participate in naive pluripotency exit. Our screen therefore provides a comprehensive dataset for better understanding the molecular mechanisms underlying exit from naive pluripotency.

### Increasing mTORC1 Activity through Gator1 or Tsc1/2 Loss Results in Opposing Phenotypes

The two mTOR-containing complexes, namely, mTORC1 and mTORC2, are crucial mediators or regulators of the PI3K-Akt pathway in response to external growth stimuli and involved in multiple processes such as translation regulation, energy metabolism, autophagy, and development (Saxton and Sabatini, 2017). mTORC1 activity is also regulated by amino acid sensing (Wolfson and Sabatini, 2017). In the previous small interfering RNA (siRNA) screen, mTORC1 regulators such as Tsc1/2, RagA/C, folliculin, and the Lamtor complex were identified (Betschinger et al., 2013). Our screen identified additional factors involved in mTORC1 regulation and thus further connects the mTOR network to pluripotency regulation (Figure 3). These factors include the Gator1, Gator2, and Kicstor complexes, as well as Stk11. At a relaxed cutoff, we were able to identify Rictor, an essential component of the mTORC2 complex. We also identified Mlst8, a factor that was identified in both mTORC1 and mTORC2 complexes but that is specifically required for mTORC2 function (Guertin et al., 2006). With the exception of Wdr24, these genes, when perturbed, demonstrated accelerated differentiation.

**Figure 4:**
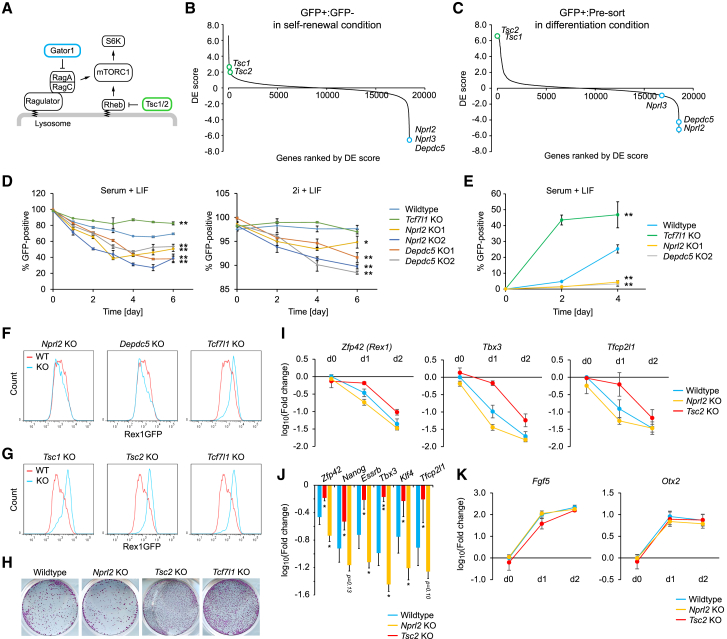
Gator1 and Tsc2 Loss Exhibit Opposing Phenotype on Naive Pluripotency Network Resolution (A) Schematic of mTORC1 regulators. (B and C) Ranked DE score plots from the self-renewal (B) and differentiation (C) screens, highlighting opposing phenotypes between Tsc1/2 and Gator1. (D) Maintenance of naive pluripotency measured as a percentage of Rex1GFP+ cells in the SL condition (left panel) and the 2iL condition (right panel). (E) Reacquisition of naive pluripotency. (F and G) RexGFP profiles of indicated KO mESCs after 27 hr differentiation for Gator1 (F) and Tsc1/2 (G) complex. *Tcf7l1* KO mESCs were used as a positive control. (H) Commitment assay. (I–K) qRT-PCR analysis of differentiating wild-type, *Nprl2* KO mESCs, and *Tsc2* KO mESCs at the indicated days. Selected naive (I and J) and formative (K) markers were analyzed. Day 1 data are summarized in (J). Expression was normalized to day 0 wild-type expression, from which log_10_(fold change) were calculated. Data are shown as mean ± SD. (D, E, and I–K) n = 3. Student’s t test was performed. ^∗^p ≤ 0.05; ^∗∗^p ≤ 0.01. See also Figures S4 and S5.

The Tsc1/2 complex functions as guanosine triphosphatase (GTPase)-activating protein (GAP) toward Rheb (Inoki et al., 2003) (Figure 4A). Genetic deletion of the Tsc1/2 complex leaves Rheb in a guanosine triphosphate (GTP)-bound active form, resulting in constitutive activation of mTORC1 (Zhang et al., 2003). The Gator1 complex also negatively regulates mTORC1 activity through its GAP activity toward RagA in response to amino acid sensing (Bar-Peled et al., 2013) (Figure 4A). Therefore, Tsc1/2 and Gator1 complexes negatively regulate mTORC1 via two distinct signaling cascades. However, in both self-renewal and differentiation screens, these two complexes showed opposing phenotypes. During self-renewal, Gator1 complex KO increased heterogeneity, while Tsc1/2 KO acted to preserve homogeneity (Figure 4B). Under differentiation conditions, Tsc1/2 deficiency resulted in strong resistance to differentiation, whereas Gator1 KO accelerated differentiation (Figure 4C). Because both mTORC1 regulators are less understood in the context of mESC self-renewal and differentiation, we sought to carry out further molecular studies on these hits.

### Gator1 Depletion Diminishes Self-Renewal and Promotes Differentiation

First, we generated *Nprl2* and *Depdc5* KO mESCs in the Rex1GFP background (Figures S5A and S5B). These KO mESCs showed indistinguishable morphology from wild-type cells and, as expected, upregulation of mTORC1 activity (Figure S5E). To investigate their self-renewal capability, we analyzed the percentage of cells retaining Rex1GFP expression after sorting GFP+ cells. In the SL condition, the GFP+ fraction in wild-type cells decreased for the first 4 days and plateaued around 70%, whereas *Tcf7l1* KO mESCs maintained a higher GFP+ fraction around 90% (Figure 4D, left panel). All *Nprl2* or *Depdc5* KO clones showed kinetics similar to wild-type cells but plateaued at a GFP+ fraction of 40%–50%, significantly lower than that of wild-type cells (Figure 4D, left panel). In the 2iL condition, there was no difference between wild-type and *Tcf7l1* KO mESCs; however, both *Nprl2* and *Depdc5* KO mESCs lost 5%–10% of the GFP+ fraction by day 6 (Figure 4D, right panel). We also investigated reactivation of *Rex1* expression by culturing sorted GFP− cells. In wild-type and *Tcf7l1* KO mESCs, approximately 20% and 50%, respectively, reactivated GFP expression by day 4, but reactivation in both *Nprl2* and *Depdc5* KO mESCs was far less efficient, resulting in only 2%–3% (Figure 4E). These results validate the Gator1 KO phenotype observed in our self-renewal screen and indicate that self-renewal capability is partially compromised in Gator1 KO mESCs.

### Tsc1/2 Depletion Delays Differentiation and Reinforces Naive Pluripotency

To analyze differentiation phenotype of the two mTORC1-negative regulators, we also generated *Tsc1* and *Tsc2* KO mESCs and confirmed expected mTORC1 upregulation (Figures S4C−S4E). We analyzed GFP profiles on day 1 of differentiation. All phenotypes observed in individual KO mESCs were in agreement with our screening results: Gator1 KO mESCs lost Rex1GFP faster than wild-type, whereas Tsc1/2 KO mESCs failed to initiate differentiation (Figures 4F and 4G). To test whether the Rex1GFP profiles correlated with cellular lineage commitment, we reseeded cells into the 2iL medium after 24 hr differentiation. We found that the number of alkaline phosphatase-positive colonies correlated with the Rex1GFP profiles (Figure 4H). To confirm, we analyzed the expression level of key naive and formative-stage markers up to day 2. All naive markers showed significant delay in downregulation in *Tsc2* KO mESCs, whereas 4 of the 6 markers tested showed significant accelerated downregulation at day 1 in *Nrpl2* KO mESCs (Figures 4I and 4J). However, upregulation of 2 formative-stage makers (*Fgf5* and *Otx2*) was not significantly affected (Figure 4K), although *Fgf5* upregulation is slightly weaker in *Tsc2* KO mESCs at day 1. We thus confirmed the effect of both mTORC1-negative regulators on naive pluripotency by individual gene targeting.

### Gsk3 Is Differentially Regulated in *Nprl2* and *Tsc2* KO mESCs

**Figure 5:**
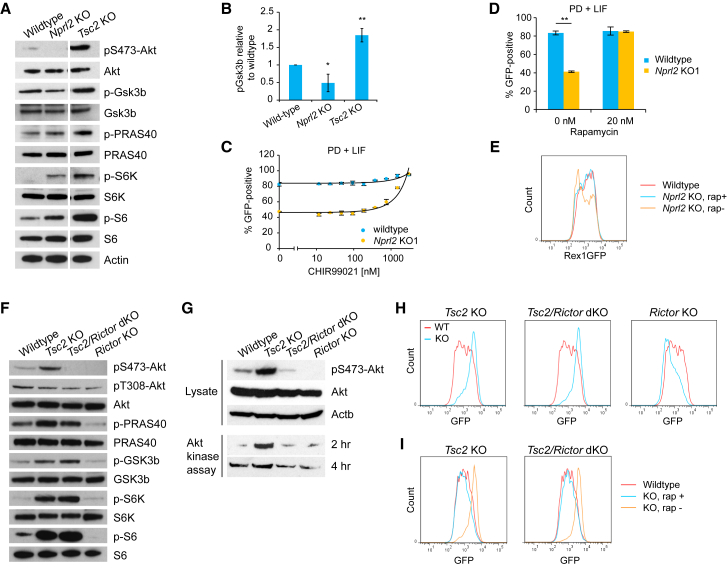
Gsk3 Is Differentially Affected by mTORC1 Upregulation upon *Nprl2* and *Tsc2* Loss (A) Western blot analysis of key phosphorylation sites in the Akt-mTORC1 pathway. (B) Quantification of the phospho-Gsk3β level. (C) Percentage of Rex1GFP+ cells in response to reducing the dose of CHIR992021 in wild-type and *Nprl2* KO mESCs. (D) Restabilization of naive pluripotency by rapamycin in *Nprl2* KO mESCs. (E) Restoration of differentiation in *Nprl2* KO mESCs by rapamycin. (F) Phosphorylation profile in *Tsc2* KO mESCs with or without *Rictor* KO. (G) Akt kinase assay. (H) Rex1GFP profile of indicated KO mESCs after 27 hr differentiation. (I) Full restoration of differentiation in both *Tsc2* sKO and *Tsc2/Rictor* dKO mESCs by rapamycin. Data are shown as mean ± SD. (B and D) n = 3. Student’s t test was performed. ^∗^p ≤ 0.05; ^∗∗^p ≤ 0.01. See also Figures S4 and S5.

mTORC1 upregulation and resulting S6K activation are known to induce the negative feedback loop and attenuate Akt activation (Zhang et al., 2006). Gsk3 is a direct downstream target of Akt and plays a central role in self-renewal and differentiation (Martello et al., 2012, Wray et al., 2011). We therefore investigated phosphorylation status of key proteins in the Akt-mTORC1 pathway. Unexpectedly, while phosphorylation on Akt-S473 was abolished as a result of negative feedback in *Nprl2* KO mESCs, *Tsc2* KO mESCs showed substantial upregulation of the phosphorylation (Figure 5A). Mirroring this pattern, Gsk3β-S9 phosphorylation was significantly downregulated in *Nprl2* KO mESCs but increased in *Tsc2* KO mESCs (Figures 5A and 5B). Another Akt target PRAS40 (also known as Atk1s1) was upregulated in both KO mESCs (Figure 5A). Altogether, both KO mESCs showed expected mTORC1/S6K upregulation but seemingly divergent phosphorylation patterns on Akt and its downstream targets.

### Increased Gsk3 Activity Destabilizes Naive Pluripotency in *Nprl2* KO mESCs

Given that Gsk3 plays a pivotal role in regulating naive pluripotency, we hypothesized that phenotypic discrepancy between *Tsc2* and *Nprl2* KO mESCs is mediated by the difference in Gsk3 regulation. To further investigate the effect of Gsk3 activity in *Nprl2* KO mESCs, we seeded GFP+ *Nprl2* KO mESCs into N2B27+1iL (MEKi + LIF) medium supplemented with serially diluted Gsk3 inhibitor and measured the percentage of GFP+ cells after 3 days. Although 80% of wild-type cells could maintain Rex1GFP expression even in the absence of the GSK3 inhibitor, *Nprl2* KO mESCs were more sensitive to the GSK3 inhibitor dose and more than 50% of the cells lost GFP expression in the absence of the inhibitor (Figure 5C). This result clearly indicates that *Nprl2* KO mESCs have elevated Gsk3 activity and thus depend more on Gsk3 inhibition to maintain Rex1GFP expression. If the negative feedback mechanism is responsible in *Nprl2* KO mESCs, mTORC1 inhibition via rapamycin should reactivate Akt and hence downregulate Gsk3, thereby rescuing phenotype in both self-renewal and differentiation. As shown in Figure 5D, rapamycin-treated *Nprl2* KO mESCs maintained Rex1GFP expression as efficiently as wild-type cells in the absence of the GSK3 inhibitor. The same treatment in differentiation conditions also rescued *Nprl2* KO phenotype, showing a Rex1GFP profile identical to that of wild-type cells (Figure 5E). Altogether, our results revealed an amino acid-sensing mediator, the Gator1 complex, as a regulator of naive pluripotency.

### Increased Akt Activation in *Tsc2* KO mESCs Does Not Contribute to Phenotypic Outcome

*Tsc2* KO mESCs showed expected mTORC1 activation (Figure 5A) and, as seen in *Tsc2* KO mouse embryonic fibroblasts (MEFs) (Zhang et al., 2006), upregulation of Gsk3 phosphorylation. However, in sharp contrast to *Tsc2* KO MEFs, we found that *Tsc2* KO mESCs showed substantial upregulation of phosphorylation on Akt-S473 (Figures 5A and 5F), suggesting that Akt is upregulated, rather than being attenuated. Overexpression of constitutively active Akt is known to sustain self-renewal in the absence of LIF and to be associated with increased Gsk3 phosphorylation (Bechard and Dalton, 2009, Watanabe et al., 2006). These observations raised the possibility that in *Tsc2* KO mESCs, upregulated Akt suppresses Gsk3 and sustains naive pluripotency.

To explore whether downregulation of Akt-S473 alters Gsk3 activity, we inactivated the mTORC2 complex by knocking out *Rictor*, an essential component of the mTORC2 complex (Guertin et al., 2006), in *Tsc2* KO and wild-type backgrounds (Figure S5F). Phosphorylation of Akt-S473 was abolished in both *Tsc2/Rictor* double-KO (dKO) and *Rictor* single-KO (sKO) cells, indicating that mTORC2 is fully responsible for the phosphorylation of Akt-S473 and that, unlike in *Tsc2* KO MEFs (Huang et al., 2008), mTORC2 was ectopically activated in *Tsc2* sKO mESCs. However, phosphorylation at T308 was only slightly affected in KO mESCs (Figure 5F). To confirm Akt activity, we performed a kinase assay using total Akt immunoprecipitated from the KO lines. Consistent with the S473 phosphorylation pattern, Akt from *Tsc2* sKO mESCs showed a markedly upregulated kinase activity, whereas Akt from both *Tsc2*/*Rictor* dKO and *Rictor* sKO mESCs showed minimal activity (Figure 5G). The lack of mTORC2 activity did not affect the mTORC1 pathway in *Tsc2*/*Rictor* dKO mESCs, as evident from the comparable phosphorylation levels on S6K and S6 (Figure 5F). Although *Rictor* sKO and *Tsc2/Rictor* dKO mESCs both showed minimal Akt activity, both Gsk3 and PRAS40 remained highly phosphorylated in *Tsc2/Rictor* dKO mESCs as in *Tsc2* sKO mESCs, but not in *Rictor* sKO mESCs (Figure 5F), suggesting that Gsk3 is not under the control of Akt in a *Tsc2*-deficient background. Consistent with the Gsk3 phosphorylation status, *Tsc2/Rictor* dKO mESCs showed delayed differentiation comparable to that observed in *Tsc2* sKO mESCs (Figure 5H). In contrast, *Rictor* sKO mESCs showed an accelerated differentiation phenotype, which is consistent with our screening data (Figure 3). It has been reported that in *Tsc2*-deficient MEFs, activated S6K constitutively phosphorylates Gsk3 and downregulates its kinase activity, which is reversible upon rapamycin treatment (Zhang et al., 2006). Consistent with the literature, rapamycin treatment fully rescued the delayed differentiation observed in both *Tsc2* sKO and *Tsc2/Rictor* dKO mESCs. Both KO cells showed identical differentiation kinetics to wild-type mESCs (Figure 5I). Altogether, *Tsc2* KO causes Akt activation in mESCs, but activated mTORC1/S6K plays a major role in influencing naive pluripotency through Gsk3 regulation.

### *Nprl2* and *Tsc2* KO Transcriptomes Reveal Differences in Naive and Formative Gene Expression

**Figure 6:**
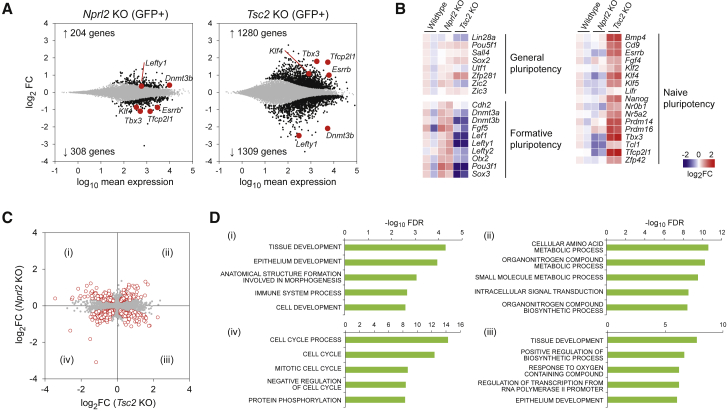
Transcriptome Profile in *Nprl2* and *Tsc2* KO mESCs (A) Depletion-enrichment sequencing (DE-seq) output of differentially expressed genes in *Nprl2* and *Tsc2* KO mESCs compared to wild-type. Genes with FDR < 0.05 were highlighted with black dots, and selected pluripotency markers were highlighted in red. (B) Expression profile of general, naive, and primed pluripotency marker genes. Primed markers were upregulated in *Nprl2* KO mESCs, while naive markers were substantially upregulated in *Tsc2* KO mESCs. (C and D) Comparison of fold changes between *Tsc2* and *Nprl2* KO mESCs. Genes that were significantly (FDR < 0.05) up- or downregulated in either or both KO mESCs were highlighted in red. (D). Gene ontology analysis of genes highlighted in each quadrant in (C).

To further explore the implications of *Nprl2* and *Tsc2* KO on stem cell properties, we performed RNA sequencing (RNA-seq) analysis on both KO lines and compared them with wild-type cells (Figure 6A). To minimize the impact that arises from the different level of heterogeneity present in each KO line, we sorted GFP+ cells before RNA isolation. We detected 512 and 2,589 differentially expressed genes in *Nprl2* and *Tsc2* KO mESCs, respectively, (FDR < 0.05). We first analyzed expression of marker genes for general, naive, and formative pluripotency markers (Figure 6B). Although general pluripotency genes were similarly expressed in all genotypes, strikingly different expression patterns were observed in naive and formative pluripotency genes between the two KO mESCs. In *Tsc2* KO mESCs, naive pluripotency genes such as *Klf4* and *Esrrb* were significantly upregulated, whereas formative pluripotency marker genes such as *Fgf5* and *Dnmt3b* were downregulated. Fold differences of the differentially expressed genes in *Nprl2* KO mESCs were generally smaller than in *Tsc2* KO mESCs, but *Nprl2* KO mESCs showed a significant downregulation in the expression of naive pluripotency genes with concomitant upregulation of formative pluripotency genes. These results indicated that Gsk3 activity levels dictate the naiveness even within the Rex1GFP+ population. *Nprl2* KO mESCs can be maintained in the SL condition, but these cells are already straddling between naive and formative pluripotent states, whereas *Tsc2* KO mESCs are more shielded from extrinsic differentiation cues and the naive state is more preferentially reinforced.

Although these two mTORC1-negative regulator KOs showed opposing phenotypes in terms of self-renewal, differentiation, and gene expression for naive and formative pluripotency markers, there should be common transcriptomic changes that stem from mTORC1 upregulation. We further analyzed the RNA-seq data by comparing fold changes relative to wild-type between *Nprl2* and *Tsc2* KO mESCs (Figure 6C) and performed gene ontology (GO) overrepresentation analysis. Consistent with the preceding observation, genes that were up- or downregulated differently between the two lines were particularly enriched in development-related processes (Figures 6C and 6D, quadrants i and iii). The analysis also detected 76 commonly upregulated genes that showed enrichment in metabolic processes (Figures 6C and 6D, quadrant ii), which are potential downstream targets of mTORC1 in mESCs. Negative regulators of cell cycle were commonly downregulated in both KO mESCs (Figures 6C and 6D, quadrant iv).

## Discussion

More than three decades of studies on mESC have revealed several genetic and epigenetic mechanisms that regulate the stem cell-defining properties of self-renewal and pluripotency. However, a fully complete and predictive overview remains elusive. This is partly due to the lack of scalable genetic methods that allow comprehensive mapping of genes to specific phenotypes. Mammalian biology has typically been studied with resource-intensive, hypothesis-driven approaches or inefficient genome-scale screens, both of which provide a limited and context-dependent account of biological processes. Hypothesis-free forward genetics applied in yeast, *Drosophila*, and *Caenorhabditis elegans* has provided deeper insights into diverse biological processes (Forsburg, 2001, Jorgensen and Mango, 2002, St Johnston, 2002). With the advent of CRISPR-Cas9 technologies, we and others have developed a CRISPR-based loss-of-function screening approach that aims to address this central relationship in the context of mammalian systems (Koike-Yusa et al., 2014, Shalem et al., 2014, Wang et al., 2014). In the present study, we have applied CRISPR-KO screening to explore the genetic basis for naive pluripotency and provide deeper insights into the long-standing question as to how the transition from naive to lineage commitment is achieved.

Unlike previously performed CRISPR-KO screens, most of which studied cell survival and/or proliferation of cultured cancer cells (Hart et al., 2015, Tzelepis et al., 2016, Wang et al., 2017), phenotypic readout in our screens was based on reporter gene expression by fluorescence-activated cell sorting (FACS) analysis. Although a few studies have used this method and found valuable hits (Burr et al., 2017, Parnas et al., 2015), this mode of genetic screening is explored less frequently, possibly due to technical difficulties in cell sorting. These difficulties may result in loss of library complexity, which severely limits the identification of meaningful hits. After a series of optimizations for high-speed cell sorting, we routinely collect 2 × 10^6^–5 × 10^6^ cells per target fraction (20×–50× coverage) and use them for gRNA amplification. The two screens we performed exhibited high sensitivity, detecting true hits in both positive and negative selection, including most genes known to be involved in self-renewal and/or differentiation. This high sensitivity was exemplified by *Tcf7l1*. Loss of *Tcf7l1* increased Rex1GFP+ cells by 15%, from 75% to 90%, in our experimental conditions (Figure 1C). Although this only translates to a marginal fold increase of 1.2, the gene was nonetheless detected in our self-renewal screen with a remarkable significance (FDR = 0.000707). The high-detection sensitivity is likely because of the deployment of our enhanced second-generation CRISPR-KO library (Tzelepis et al., 2016), which could potentially be further improved by using optimization metrics outlined in our recent study (Ong et al., 2017). Another approach to increase detection sensitivity would be the incorporation of the recently developed CRISPR-UMI technology (Michlits et al., 2017). This technology allows us to trace individual mutant clones. Multiple phenotype caused by heterogenous mutant alleles (as exemplified by *Pou5f1* in this study) could be more sensitively detected. Our screening method described here provides the basis for FACS-based genetic screens to the wider research community and should serve as a useful example of its deployment.

Our CRISPR-KO differentiation screen evaluating the exit from pluripotency yielded 575 gene hits (563 and 12 hits for positive and negative selection, respectively). We optimized this screen to perform positive selection to detect genes whose mutation causes sustained Rex1GFP expression. It is therefore not surprising that a smaller number of genes were detected in negative selection (i.e., genes showing accelerating differentiation). We used a relaxed cutoff (FDR of 20%) for negative selection, but *Rictor* (FDR = 0.506) could be validated by individual gRNA and in *Rictor* sKO mESCs. This suggests that although higher noise is expected, some genes under the suboptimal threshold can potentially be meaningful and worthy of further investigation. For example, Sestrin2, encoded by *Sesn2*, has been characterized as a leucine sensor, and its loss results in continuous mTORC1 activation even in the absence of leucine (Wolfson et al., 2016). *Sesn2* was not a significant hit (FDR = 0.609) but nonetheless ranked at 46 in the negative selection. Correspondingly, a leucine transporter, Slc7a5, was detected in the positive selection with an FDR of 0.0038. These again highlight the high sensitivity of CRISPR-KO screens, but there is clearly a room for further improvements to this sensitivity with regards to negative selection; it would be worthwhile to uncover more genes that show accelerated differentiation.

**Figure 7:**
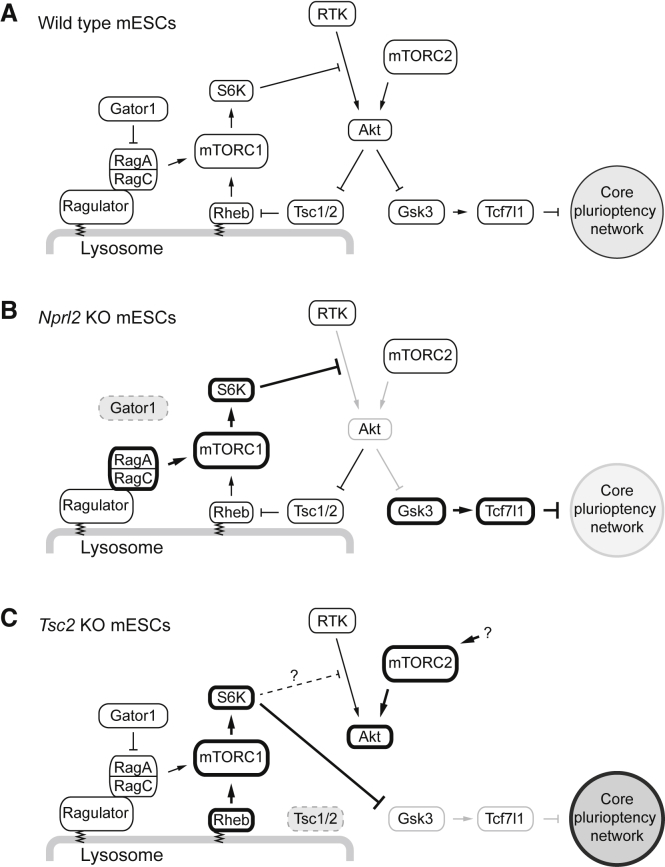
Models of mTORC1-Mediated Gsk3 Regulation in Each Genotype (A) In wild-type cells, receptor tyrosine kinase (RTK)-mediated activation of Akt and the mTORC1-mediated negative feedback are in equilibrium, maintaining appropriate Gsk3 activity level. (B) *Nprl2* loss increases mTORC1 activity and shows stronger negative feedback, resulting in Gsk3 upregulation. (C) *Tsc2* loss also increases mTORC1 activity, but upregulated S6K directly phosphorylates and consequently inactivates Gsk3 (Zhang et al., 2006). mTORC2 is upregulated in the absence of Tsc2 protein in mESCs.

The previously performed screens have identified genes required for differentiation, but there has been no screen that has analyzed accelerated differentiation upon naive exit. Many genes identified from the negative selection have not been previously described in the context of pluripotency regulation and were surprisingly overrepresented with mTOR-related mediators. mTOR KO embryos exhibit post-implantation lethality at embryonic day 5.5–6.5, and mTOR KO mESCs cannot be established from KO blastocysts (Murakami et al., 2004). It has been shown that mouse blastocysts and mESCs treated with mTORC1/2 inhibitors undergo proliferation arrest with maintaining pluripotency, mimicking diapaused embryos (Bulut-Karslioglu et al., 2016). These data indicate that mTOR activity is mainly required for cell proliferation, but our present data suggest that mTOR activity influences the equilibrium of the core naive pluripotency maintenance network through the Gsk3-Tcf7l1 axis (Figure 7).

First, mTORC2 was identified from the negative selection with *Rictor* and *Mlst8*. Through *Rictor* KO mESCs, we demonstrated that mTORC2 deficiency causes a reduction of Akt activity and consequently increases Gsk3 activity. Mlst8 is a component common to both mTORC1 and mTORC2, but it has been shown that Mlst8 is essential for mTORC2, but not for mTORC1 (Guertin et al., 2006). Another finding is that mTORC1 activation by loss of mTORC1-negative regulators in the amino acid-sensing pathway (Gator1, Kicstor, and Sestrin2) leads to destabilized pluripotency maintenance (Figure 7B). We have provided genetic evidence using *Nprl2* KO cells. Because Kicstor and Sestrin2 KO cells in human cancer cells consistently showed mTORC1 upregulation (Wolfson et al., 2016, Wolfson et al., 2017), genetic disruption of these genes in mESCs would mirror the phenotype observed in Gator1 KO mESC. Therefore, our finding connects mTORC2 and the amino acid-sensing pathway to the core pluripotency maintenance network through Akt-Gsk3-Tcf7l1.

We identified another mTORC1-negative regulator, the Tsc1/2 complex, which showed the opposite phenotype compared to Gator1 complex, namely, delayed differentiation. It has been shown that in *Tsc2* KO MEFs, Akt is inactive due to the negative feedback, but activated S6K phosphorylates Gsk3 (Zhang et al., 2006). This Gsk3 phosphorylation can be attenuated by mTORC1 downregulation (Zhang et al., 2006). Our observation in *Tsc2* KO mESCs is in agreement with this previous finding, and the observed differentiation phenotype can be explained by Gsk3 phosphorylation. Therefore, Tsc1/2 is also connected to the core pluripotency network, but the effect is opposite that of the Gator1 complex due to the rewiring of the phosphorylation network (Figure 7C).

Our observations in two mTORC1-negative regulators provide further insights into general functions of these complexes. First, Tsc1/2 complex may play a critical role in regulating Akt-Gsk3 interaction. S6K is activated in both *Tsc2* and *Nprl2* KO mESCs through mTORC1 upregulation, yet only in *Tsc2* KO cells does S6K phosphorylate Gsk3. In *Nprl2* KO mESCs, upregulated S6K seemingly causes conventional negative feedback and attenuates Akt and consequently Gsk3 phosphorylation. Therefore, Gsk3 phosphorylation by activated S6K is specific to the *Tsc2*-deficient background. Second, we unexpectedly found that Tsc2 loss resulted in the upregulation of Akt-S473 phosphorylation, indicative of mTORC2 upregulation. Rictor loss abolishes Akt-S473 phosphorylation, providing evidence for mTORC2 upregulation. It has been shown that the Tsc1/2 complex is required for proper activation of mTORC2 (Huang et al., 2008). This difference suggests that at least in mESC, Tsc2 plays a suppressor role in mTORC2 regulation. Although these might be due to the transcriptomic changes or the change in cell fate caused by gene KO, patients with Tsc1/2 or Gator1 deficiency show a different clinical phenotype (Dabora et al., 2001, Ricos et al., 2016), suggesting undiscovered roles, in addition to conventional mTORC1 regulation, that would be worthy of further investigation.

Together with the screen on self-renewal, our differentiation screen provides an invaluable resource to further understand naive pluripotency regulation and the genes required for the induction of cellular differentiation. The molecular function of some hits (e.g., the KICSTOR complex) were only recently characterized. Further interrogation of the data presented here can be useful not only in understanding pluripotency regulation but also in uncovering the fundamental molecular functions involved. The success of our screening approach indicates that with appropriate reporter systems, pooled CRISPR-KO screens can be a powerful approach for fueling insights into stem cell biology and can intimately dissect the molecular pathways that positively or negatively influence differentiation. Proliferation-essential genes in human ESCs have been characterized by genome-wide CRISPR screening (Yilmaz et al., 2018). Cellular differentiation of human pluripotent stem cells has not yet been studied extensively, and such studies would facilitate better understanding in disease mechanisms and generate more efficient differentiation protocols for cell therapy. In addition, several studies have reported the successful derivation of human naive pluripotent stem cells (Takashima et al., 2014, Theunissen et al., 2014). It would be of great interest to investigate whether our findings are recapitulated in the context of human naive pluripotency, which will lead to a greater understanding of the molecular basis of differentiation and lineage commitment. As CRISPR-KO screening technology continues to be developed and improved, it would be beneficial to apply functional genomic approaches to answer such central questions in stem cell biology.

## Experimental Procedures

### Cell Culture

A Rex1GFP mESC line (Wray et al., 2011), was a gift from Austin Smith and cultured on feeder cells in SL: KO-DMEM (Thermo Fisher Scientific) supplemented with 15% fetal bovine serum (Thermo Fisher Scientific), 1% GlutaMAX (Thermo Fisher Scientific), 1% nonessential amino acid (NEAA) (Thermo Fisher Scientific), 0.1 mM 2-mercaptoethanol (Sigma), and 1,000 U mL^−1^ LIF (Millipore). Where indicated, mESCs were cultured on gelatin-coated plates in 2iL medium: NDiff227 (Takara) supplemented with 1% KO serum replacement (KSR) (Thermo Fisher Scientific), 5% BSA (Thermo Fisher Scientific), 1% NEAA, 0.1 mM 2-mercaptoethanol, 1,000 U mL^−1^ LIF, 1.0 μM PD0325901 (Selleck), and 3.0 μM CHIR90021 (Selleck). Differentiation was induced in the NDif227 medium supplemented as mentioned earlier but without the 2 inhibitors and LIF.

### CRISPR-KO Screen on Self-Renewal

Cells (3.2 × 10^7^) were transduced with the mouse v2 CRISPR library (Tzelepis et al., 2016). On day 2, approximately 1.0 × 10^7^ cells double positive for GFP and blue fluorescent protein (BFP) were collected by sorting. Half of them were cultured on feeder cells in SL, and the other half were in 2iL medium. Thirty million cells were reseeded at every passage to maintain 300× coverage. On days 8 and 15 post-transduction, cells in SL were sorted based on GFP expression and genomic DNA was isolated. Cells in 2iL were directly subjected to genomic DNA isolation.

### CRISPR-KO Screen on Exit from Pluripotency

Transduction and sorting on day 2 were performed as described earlier. Sorted cells were cultured for an additional 4 days in 2iL medium. On day 6, cells were trypsinized and 45 million cells were plated on eight 15-cm dishes (10,000 cells cm^−1^) in NDiff227 differentiation medium. After 2 days, cells were trypsinized and 20 million cells were kept as a pre-sort control. The remaining cells were used for sorting, and approximately 3 million GFP+ cells (top 2%–3%) were collected. Genomic DNA from the pre-sort and the GFP+ fraction were isolated.

### Statistical Analyses

Statistical analyses of CRISPR screens were performed by MAGeCK (Li et al., 2014). Statistical tests of quantitative data were performed by Student’s t test as indicated in each figure.

## Data and Software Availability

The accession number for the RNA-seq data reported in this paper is GEO: GSE107060. The accession number for the CRISPR-KO screening data, including raw read counts and MAGeCK output, reported in this paper is BioStudies: S-BSST61.

## Supplemental Information

Document S1. Supplemental Experimental Procedures, Figures S1–S5, and Tables S3–S7

Table S1. DE Scores for All MAGeCK Analyses in Self-Renewal and Differentiation Screens, Related to Figures 1, 2, 3, and 4

Table S2. Hit Gene List, Related to Figures 1, 2, and 3

Document S2. Article plus Supplemental Information
