# Supplementary material for: Genome-wide CRISPR-KO Screen Uncovers mTORC1-Mediated Gsk3 Regulation in Naive Pluripotency Maintenance and Dissolution
Source: Cell Rep. 2018 Jul 11;24(2):489–502. doi: 10.1016/j.celrep.2018.06.027 (PMC6057492; doi:10.1016/j.celrep.2018.06.027)
Supplement: Document S1. Supplemental Experimental Procedures, Figures S1–S5, and Tables S3–S7 [file mmc1.pdf]

Cell Reports, Volume *24*

Supplemental Information

Genome-wide CRISPR-KO Screen Uncovers mTORC1-Mediated Gsk3 Regulation in Naive Pluripotency Maintenance and Dissolution

Meng Li, Jason S.L. Yu, Katarzyna Tilgner, Swee Hoe Ong, Hiroko Koike-Yusa, and Kosuke Yusa

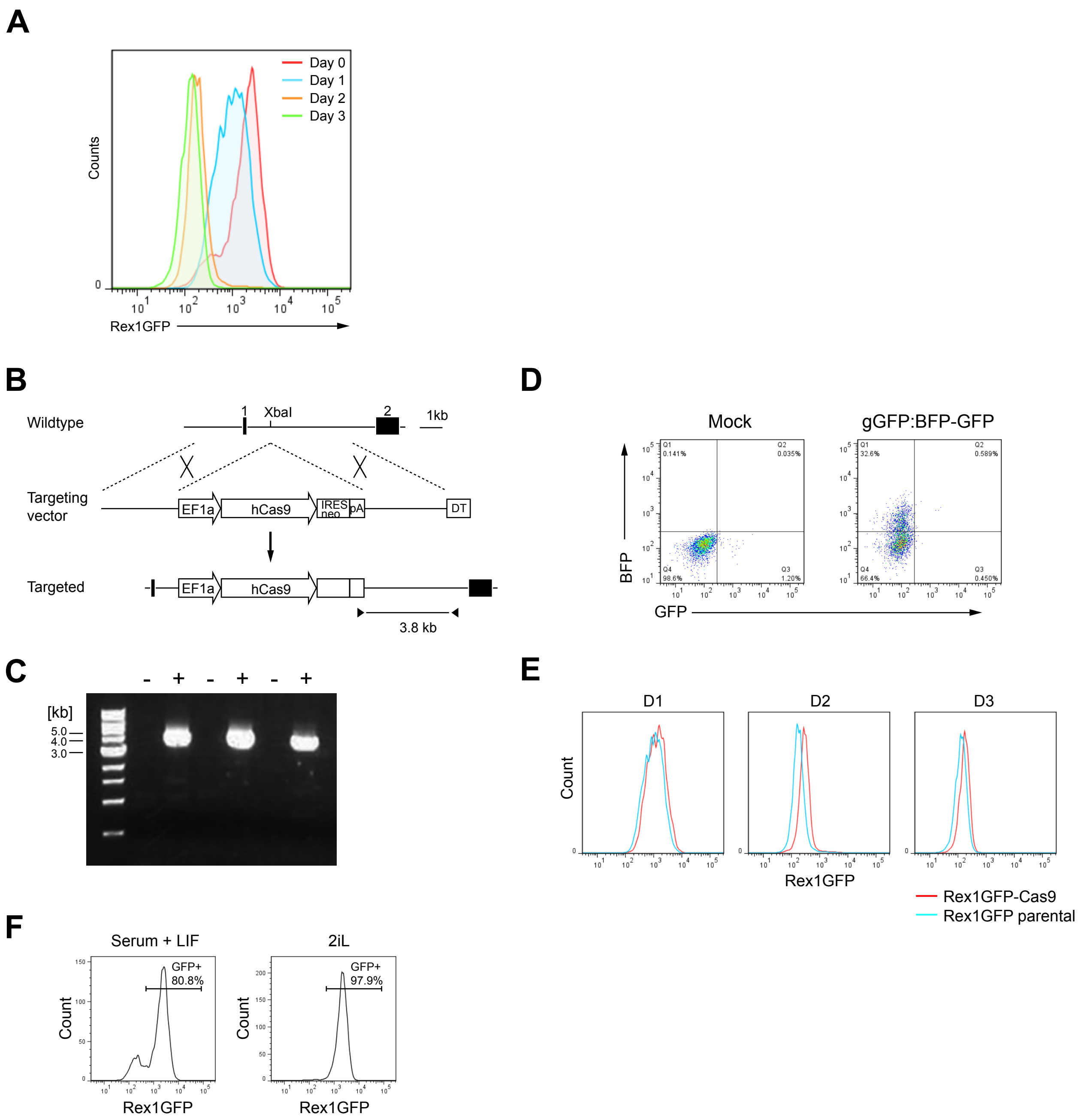

Figure S1. Generation and characterisation of CRISPR-ready Rex1GFP mESCs, Related to Figures 1 and 2.

A. Rex1GFP profile during differentiation in N2B27. **B**. Knock-in strategy of the Cas9 expression cassette at the mouse *Rosa26* locus. The targeting vector used was described in (Tzelepis et al., 2016). **C**. Detection of targeted insertion by long-range PCR. **D**. Cas9 functional assay in Cas9-expressing cells. Two days after transduction, cells were differentiated in N2B27 to remove GFP signal derived from the *Rex1* locus. **E**. Comparison of Rex1GFP profiles with or without Cas9 expression. **F**. Rex1GFP profiles in cells cultured in SL or 2iL condition.

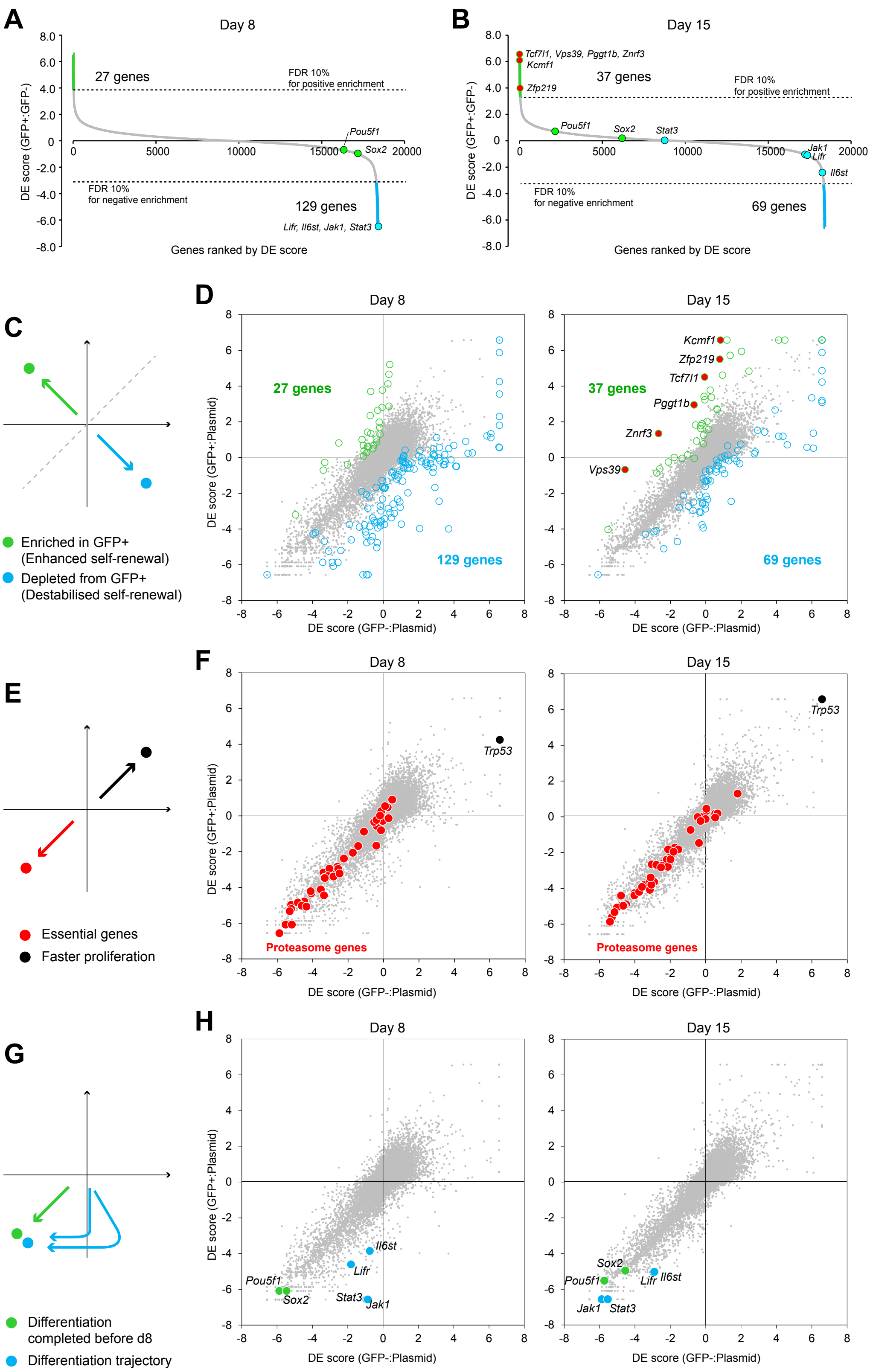

Figure S2. Patterns of signature genes in proliferation-DE plots, Related to Figure 1

A-B. Genes ranked by DE score derived from MAGeCK analysis between GFP+ and GFP- populations on d8 (A) and d15 (B) as shown in Figure 1B and C, respectively. **C-D**. Genes significantly enriched in (green) or depleted from (blue) the GFP+ population (shown in A and B with the same colour) are highlighted in the DE plots. DE scores in these plots were derived from MAGeCK analysis between plasmid counts and GFP- (x axis) or GFP+ (y axis) population; hence, these DE scores represent proliferation phenotype in each population. Genes validated in Figure 1D are highlighted in D (right panel). **E-F**. Genes commonly essential (e.g. Proteasome genes) or giving growth advantage (e.g. *Trp53*) are located in the bottom-left or top-right quadrant, respectively, along the +45° diagonal line. **G-H**. Trajectory of known pluripotency maintenance genes. *Pou5f1* and *Sox2* show rapid differentiation after knockout and depletion from the entire population; therefore, these genes were detected as essential to both population (the bottom-left corner on the diagonal line). On the other hand, genes in the LIF-Stat3 pathways showed depletion from GFP+ population on d8 and then from the entire population by d15.

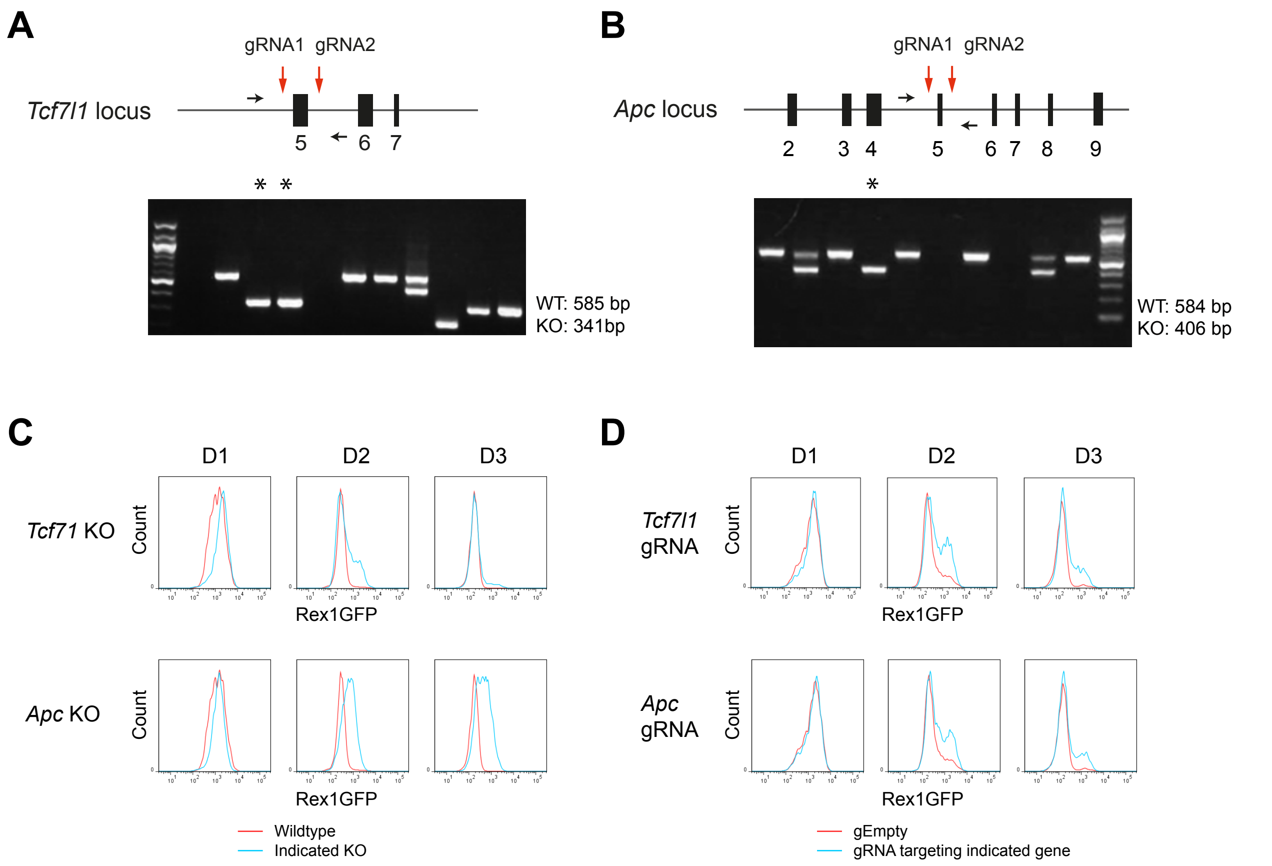

Figure S3. Generation of Tcf7l1 and Apc KO mESCs and their phenotype on differentiation, Related to Figure 2.

A-B. CRISPR-based gene KO and genotyping strategy of *Tcf7l1* (A) and *Apc* (B). **C**. Rex1GFP profiles of indicated KO mESCs between d1 and d3 differentiation. **D**. Rex1GFP profiles of bulk KO mESCs generated by lentiviral gRNA expression. Astariscs indicate knockout clones.

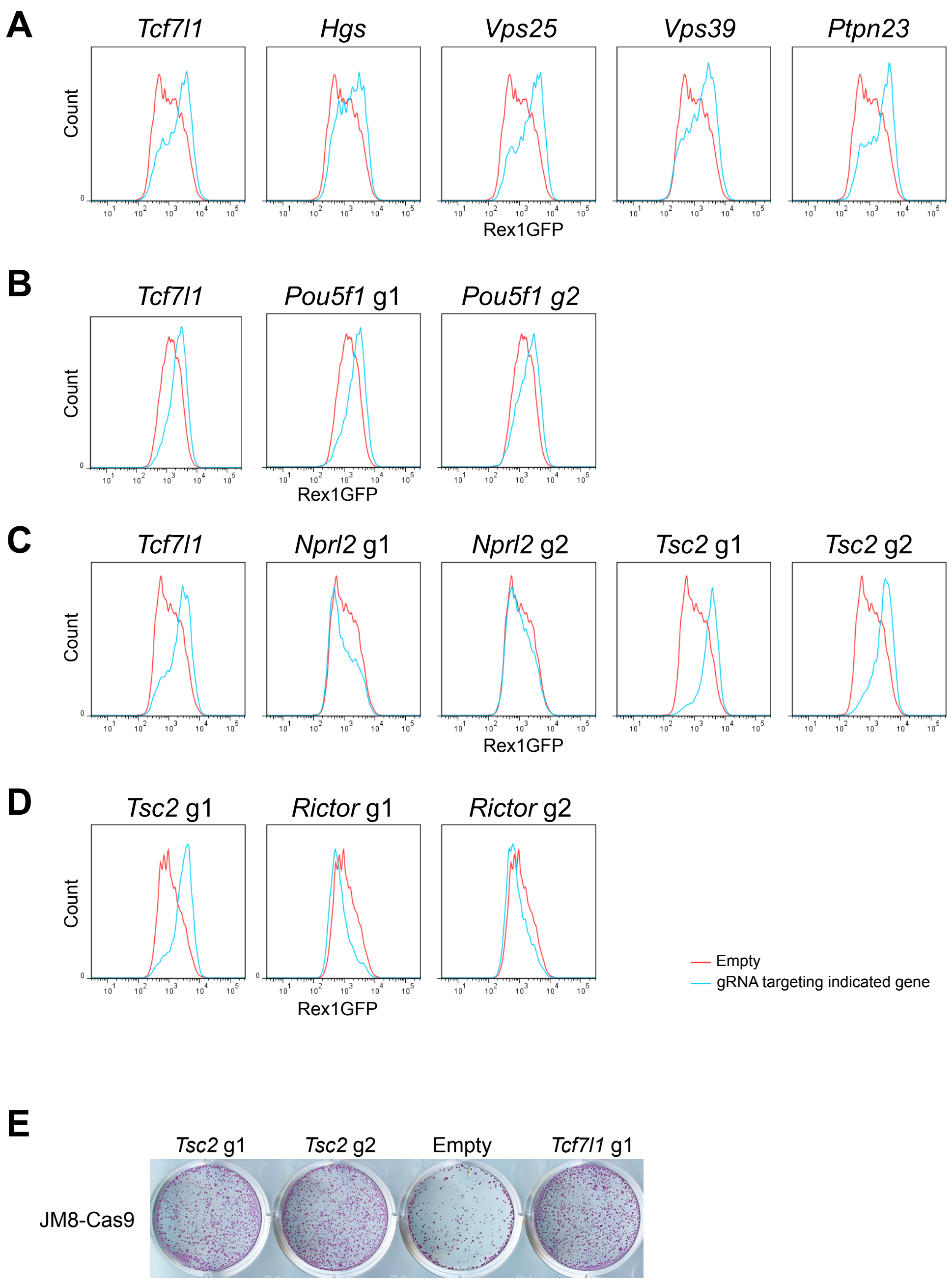

Figure S4. Validation of differentiation screen hits, Related to Figures 3,4 and 5.

A-D. Rex1-Cas9 mESCs were transduced with lentiviral expressing indicated gRNA. 5-7 days post transduction, BFP+/GFP+ cells were collected by sorting and differentiated in Ndiff227 differentiation medium for 27 h. Genes involved in vesicle trafficking (A), *Pou5f1* (B), negative regulators of mTORC1 (C) and mTORC2 (D) were disrupted by lentiviral gRNA expression. *Tcf7l1* (A-C) and *Tsc2* (D) were used as positive controls. **E**. *Tsc2* KO phenotype was also confirmed in a different mESC line. JM8-Cas9 cells were transduced with lentivirus expressing *Tsc2* gRNA. Seven days post transduction, BFP+ cells were collected by sorting, differentiated for 24h and then re-seeded in 2iL medium. Resulting colonies were stained with alkaline phosphatase kit.

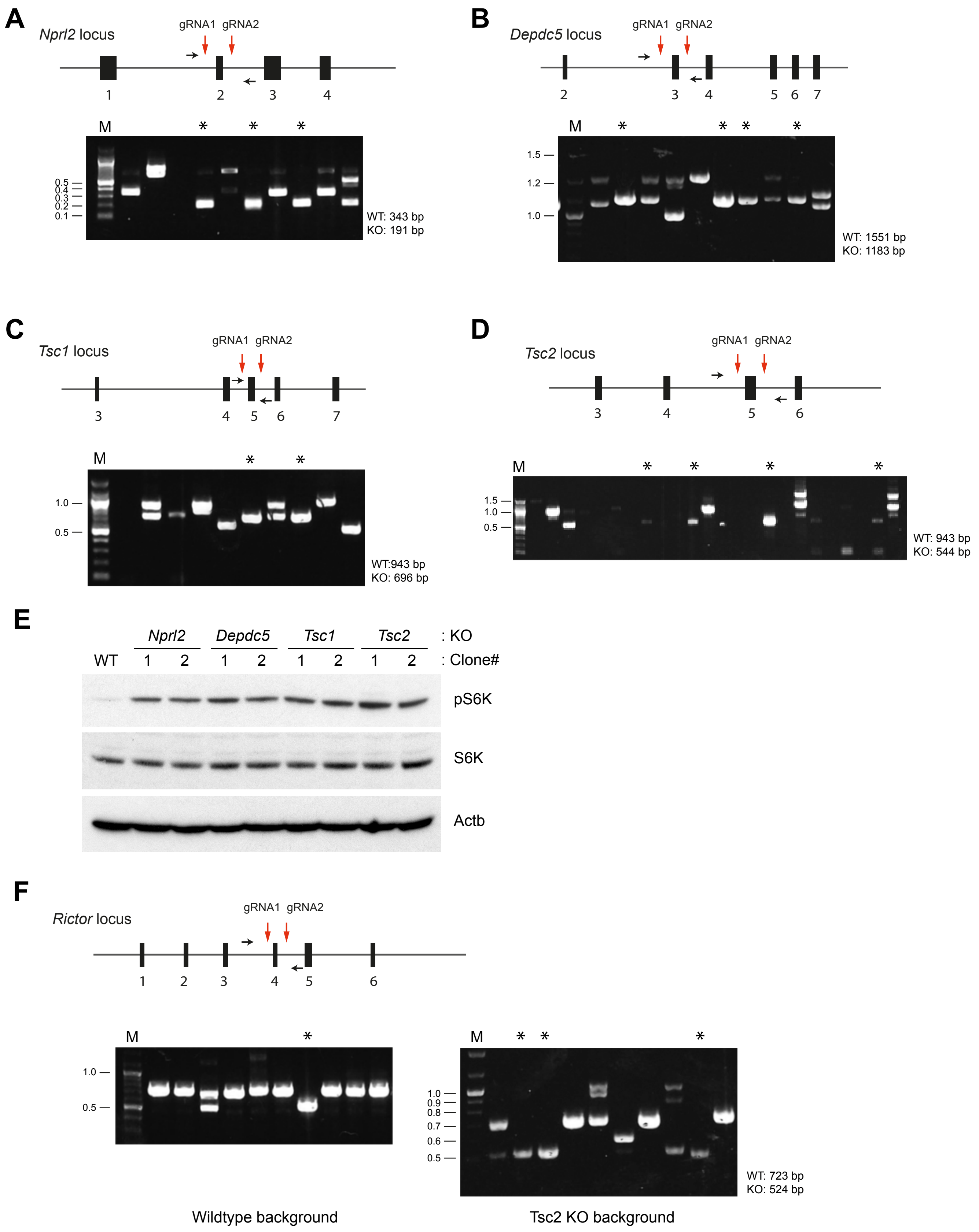

Figure S5. Generation of *Nprl2*, *Depdc5*, *Tsc1*, *Tsc2* and *Rictor* KO mESCs, Related to Figures 4 and 5.

A-D. CRISPR-based gene KO and genotyping strategy of *Nprl2* (A), *Depdc5* (B), *Tsc1* (C) and *Tsc2* (D). **E**. Western blots showing mTORC1 activation as upregulated phospho-S6K. **F**. CRISPR-based gene KO and genotyping strategy of Rictor. Asterisks indicate knockout clones.

Supplemental Experimental Procedures

Cell culture

A mESC line, Rex1dGFP-IRES-Bsd (Wray et al., 2011), was a kind gift from Austin Smith. mESCs were cultured on mitomycin C-treated mouse embryonic fibroblasts (MEFs) in SL (Serum+LIF): Knockout DMEM (Thermo Fisher) supplemented with 15% foetal bovine serum (Thermo Fisher), 1 % GlutaMax (Thermo Fisher), 1 % nonessential amino acids (Thermo Fisher), 0.1 mM 2-mercaptoethanol (Sigma) and 1,000 U ml^{-1} leukaemia inhibitory factor (LIF; Millipore). Where indicated, mESCs were cultured on gelatin-coated plates in 2iL medium: NDiff227 (Takara) supplemented with 1% knockout serum replacement (Thermo Fisher), 5 % BSA (Thermo Fisher) , 1 % nonessential amino acids, 0.1 mM 2-mercaptoethanol, 1,000 U ml^{-1} LIF, 1.0 μM PD0325901 (Selleck) and 3.0 μM CHIR90021 (Selleck). Differentiation was induced in NDif227 medium supplemented with 1% knockout serum replacement (Thermo Fisher), 5 % BSA (Thermo Fisher) , 1 % nonessential amino acids and 0.1 mM 2-mercaptoethanol. MEFs were cultured in DMEM (Thermo Fisher) supplemented with 15% foetal bovine serum, 1 % GlutaMax, 1 % nonessential amino acids, 0.1 mM 2-mercaptoethanol and mitotically inactivated by mitomycin C (Sigma) treatment at 15 μg ml^{-1} for 2.5 h before use. 293FT (Thermo Fisher) cells were cultured in DMEM (Thermo Fisher) containing 10% FBS and 1% GlutaMax. All cell lines used in this study are negative for mycoplasma contamination.

Generation of Rex1-Cas9 cell lines

The human elongation factor 1α promoter-driven Cas9 expression cassette was knocked into the *Rosa26* locus as described previously (Tzelepis et al., 2016). Cas9 expression was tested using a Cas9 reporter system with pKLV2-U6gRNA5(gBFP)-PGKmCherry2ABFP-W (Tzelepis et al., 2016). A selected cell line ($Rex1^{GFPd2\text{-}IRES\text{-}Bsd/+}$;$Rosa26^{EF1a\text{-}hCas9\text{-}IRES\text{-}neo/+}$) was designated as Rex1-Cas9 mESCs.

CRISPR-KO screen on self-renewal

Thirty two million Rex1-Cas9 cells were transduced with the mouse v2 CRISPR library (Tzelepis et al., 2016) at a pre-defined condition that gave rise to 30% transduction efficiency as described before (Koike-Yusa et al., 2014). On day2, approximately 10 million cells double positive for GFP and BFP were collected by cell sorting. Of these, 5 million cells were further cultured on mitomycin C-treated MEFs in SL and the other 5 million cells were cultured in 2iL medium. Thirty million cells were plated at each passage to maintain 300x representation of each gRNA. On d8 and d15 after transduction, cells from the SL condition were sorted according to their GFP expression and genomic DNA was isolated from each fraction using DNeasy Blood and Tissue kit (Qiagen). Cells from the 2iL condition were directly subjected to genomic DNA isolation using Blood and Cell Culture DNA Maxi kit (Qiagen).

CRISPR-KO screen on exit from pluripotency

Transduction and cell sorting on day 2 were performed as described above. Sorted cells were plated on gelatin-coated plate in 2iL medium and cultured for an additional 4 days. On day 6, cells were trypsinised and a total of 45 million cells were plated on eight 15-cm dishes (10,000 cells cm^{-1}) in NDiff227 differentiation medium to induce differentiation. After 2 days of differentiation, cells were trypsinised and 20 million cells were kept as a pre-sort control. The remaining cells were used for cell sorting and approximately 3 million GFP+ cells (top 2-3 %) were collected. Genomic DNA from the pre-sort and GFP+ fraction were isolated using Blood and Cell Culture DNA Maxi kit (Qiagen) and DNeasy Blood and Tissue kit, respectively. This screen was performed in 4 biological replicates.

gRNA sequencing

gRNA amplification from genomic DNA and Illumina sequencing were performed as described previously (Koike-Yusa et al., 2014) with minor modifications. As the total amount of genomic DNA from the sorted populations (2-3 million cells) is limited, all available genomic DNA (typically 5 μg) was used in the first-round PCR at 200 ng per reaction in 24 reactions to maximise the coverage.

CRISPR-KO screen data analysis

Data analysis was performed essentially as described previously. Briefly, read counts were generated using an in-house gRNA counting script and then statistical analysis was performed using MAGeCK (Li et al., 2014). DE scores were calculated from gene-level significance returned by MAGeCK with the following formula:

DE score = $\log_{10}$(gene-level depletion P value) – $\log_{10}$(gene-level enrichment P value).

GSEA was performed using the GSEA pre-ranked module on GenePattern (https://genepattern.broadinstitute.org/). A gene rank based on DE scores was used as a rank list.

Generation of KO cell lines by exon deletion

Genes listed in Supplementary Table 3 were knocked out by deleting a 'critical' exon, which would most likely induce nonsense-mediated decay when deleted. Two gRNAs, one each in the upstream and downstream intron, were designed using WGE (Supplementary Table 3) and cloned into pKLV2-U6gRNA5(BbsI)-PGKpuroBFP-W (Tzelepis et al., 2016). Rex1-Cas9 cells were co-transfected with a pair of gRNAs using LipofectamineLTX as described previously (Koike-Yusa et al., 2014). BFP-positive cells were then collected 3 days after transfection by cell sorting (BD Influx or Mo-Flo) and plated on 10-cm dishes at a clonal density. Colonies were subsequently picked and genotyped using primers listed in Supplementary Table 4 as described before (Koike-Yusa et al., 2014). Clones that carried expected deletion on both alleles were further expanded (Supplementary Fgis. 3, 5).

Generation of KO mESCs by lentiviral CRISPR

Genes listed in Supplementary Table 5 were knocked out by transducing Rex1-Cas9 cells with gRNA-expressing lentivirus. gRNAs were selected from our v2 mouse library based on the degree of phenotype, and cloned into pKLV2-U6gRNA5(BbsI)-PGKpuroBFP-W. Lentiviral transduction was performed as described previously (Koike-Yusa et al., 2014). Cells were further cultured and used for assays at indicated time points.

Self-renewal assay

GFP+ or GFP- cells were sorted from parental cells and *Tcf7l1*, *Nprl2* or *Depdc5* KO mESCs and cultured either on feeder layers in M15L or on gelatin-coated dishes in 2iL. On indicated days, cells were harvested and analysed by flow cytometry (BD Fortessa II).

Differentiation assay

3.8 x 10^5 Rex1GFP$^+$ cells were collected by cell sorting from wildtype or KO cell lines that had been maintained in SL condition and plated on a well of gelatin-coated 12-well plates in NDiff227 differentiation medium. When gene knockout was conducted by lentiviral CRISPR, 3.8 x 10^5 cells double-positive for GFP and BFP were collected. Rex1GFP expression was measured 24-27 h post plating, unless otherwise stated. Where indicated, rapamycin (20 nM) or DMSO was added when sorted cells were plated for differentiation. For replating assay, cells were differentiated as described above for 28 h, trypsinised, counted and replated into 2iL medium at 20,000 cells per well of 12-well plate. After 6-8 days, cells were stained with alkaline phosphatase staining kit (Sigma) and colonies were counted.

RNA-seq and data analysis

GFP+ cells were collected by cell sorting from wildtype, *Nprl2* KO and *Tsc2* KO mESCs and used for total RNA isolation with RNeasy mini kit (Qiagen). Total RNA was then converted into Illumina libraries using KAPA Stranded mRNA-Seq Kit (KAPA Biosystems) and sequenced by paired-end 75-bp sequencing on Illumina HiSeq2500. Transcript counts were generated using Kalisto (Bray et al., 2016) and differential expression was analysed using

DEseq2 (Love et al., 2014). Gene ontology (GO) analysis was performed using GO_Biological Processes on the GSEA platform (http://software.broadinstitute.org/gsea/).

Western blot

mESCs were directly lysed on tissue culture plates by adding RIPA buffer (Sigma) supplemented with phosphatase inhibitor (Sigma, 1:100) and protease inhibitor cocktails (Sigma, 1:1000). Lysates were then centrifuged at 15,000 rpm for 15 minutes at 4 °C, and supernatant was collected and stored at -80 °C. Total protein concentration was quantified using Bradford Protein Assay (BioRad) following the manufacturer's protocol. The quantified protein samples were diluted with 4x sample buffer (Thermo Fisher) supplemented with Reducing agent (Thermo Fisher) and heat-denatured at 70 °C for 10 min. The denatured protein samples were loaded at 5-20 µg per lane on a 4-12% gradient Bis-Tris gel (Thermo Fisher) and resolved at 180 V for 1 h in MOPS-SDS running (Thermo Fisher). Dissolved proteins were then transferred onto a PVDF membrane (Millipore) at 90 V for 1 h at 4 °C by wet transfer. Membranes were blocked for 30 minutes in either 5 % BSA (Sigma) or 5 % milk (Sigma) in TBST buffer (TBS supplemented 0.1 % Tween 20) and then probed in diluted primary antibody overnight at 4 °C. The membranes were washed three times in TBST and then probed with appropriate horse radish peroxidase-conjugated secondary antibody for 1 h at room temperature. The membranes were washed three times in TBST, incubated with ECL (Roche) for 5 min at room temperature, and exposed onto an X-ray film.

Kinase assay

mESCs were feeder-depleted by brief incubation on gelatinised dishes in SL medium and allowed to plate down on a fresh 10 cm dish overnight. Cells were then lysed and processed as per Akt Activity Assay Kit (Abcam) with modifications. In brief, Akt was immunoprecipitated via overnight incubation with pan-specific Akt antibody followed by 2 h incubation with Protein-A dynabeads (Thermo Fisher) both at 4 °C with agitation. Beads were then washed and incubated with GSK-3α substrate/ATP reaction mix at 30 °C for 2 or 4 h. Reaction was halted by the addition of 4x sample buffer and processed for immunoblotting to analyse the phosphorylation status of the GSK-3α substrate. All antibodies were supplied by the kit and dilutions were used as indicated in the supplied protocol.

RT-qPCR

Rex1GFP+ cells from wildtype, *Nprl2* KO and *Tsc2* KO cells that had been maintained in SL medium were collected by cell sorting and 5.0×10^5 cells were plated to a gelatin-coated 10-cm dish in NDiff227 differentiation medium. Cells were collected immediately after sorting as d0 samples and after differentiation for 1 and 2 days, and used for total RNA isolation. Reverse transcription was performed using SuperScript III (Thermo Fisher) according to the manufacturer's instruction. qPCR was then performed using Agilent Mx3005P qPCR system with Brilliant III Ultra-Fast SYBR Green PCR Mix with Low ROX (Agilent) and primers listed in Supplementary Table 6.

Table S1. DE scores for all MAGeCK analyses in self-renewal and differentiation screens (Excel file), Related to Figures 1-4.

Table S2. Hit gene list (Excel file), Related to Figures 1-3.

Table S3. Representative Reactome/KEGG gene sets positively enriched in the differentiation screen, Related to Figures 2 and 3.

REACTOME/KEGG gene set	Key process	NES	FDR q
REACTOME_RESPIRATORY_ELECTRON_TRANSPORT	Mitochondria	2.61	0
REACTOME_MITOCHONDRIAL_TRNA_AMINOACYLATION	Mitochondria	2.17	0
KEGG_OXIDATIVE_PHOSPHORYLATION	Mitochondria	2.59	0
REACTOME_GLYCOLYSIS	Glucose metabolism	1.66	0.044
REACTOME_MICRORNA_MIRNA_BIOGENESIS	miRNA	1.59	0.065
REACTOME_METABOLISM_OF_MRNA	mRNA	1.98	0
REACTOME_DESTABILIZATION_OF_MRNA_BY_AUF1_HNRNP_D0	mRNA	1.66	0.042
KEGG_RNA_DEGRADATION	mRNA	2.17	0
KEGG_PROTEASOME	Proteasome	1.77	0.008
KEGG_GLYCOSYLPHOSPHATIDYLINOSITOL_GPI_ANCHOR_BIOSYNTHESIS	GPI anchor	1.80	0.005
KEGG_GLYCOSAMINOGLYCAN_BIOSYNTHESIS_HEPARAN_SULFATE	Heparan sulfate	1.87	0.002
REACTOME_FRS2_MEDIATED_CASCADE	FGF-MAPK	1.71	0.032
REACTOME_PROLONGED_ERK_ACTIVATION_EVENTS	FGF-MAPK	1.63	0.058
REACTOME_INSULIN_RECEPTOR_SIGNALLING_CASCADE	PI3K-AKT-mTORC1 -FGF-MAPK	1.53	0.101
REACTOME_SIGNALING_BY_WNT	Wnt	1.82	0.009
REACTOME_ENDOSOMAL_SORTING_COMPLEX_REQUIRED _FOR_TRANSPORT_ESCRT	Endosome-trafficking	1.81	0.009
REACTOME_ER_PHAGOSOME_PATHWAY	Endosome-trafficking	1.70	0.033

Table S4. gRNAs used to generate KO clones, Related to Figures 2,4 and 5.

Gene	gRNA1	gRNA2
Tcf7l1	GCTCCCAAAGAGCGGTGGTG	TGAAAGGAGCCACCGGTGAG
Apc	ACAAGCTAATACATATTGCC	GACAGTGCAGCTTTTAGATT
Nprl2	CCGGACCCAACGTCCACTGA	GTGTGAGGCTTTAGTTGGGT
Depdc5	CACAGGGCACCCCATCATGT	ACAAAACATGCTCGTCTCTA
Tsc1	GGCGACATCAGGCTCAGCAC	GCAGCCATGTGTATGCGGGA
Tsc2	AAAAGGTGCTGCAGTTCACG	TCCAAGCTTAATGCATTAGG
Rictor	TGCTTGTCTATGCACAATTT	TAACAATTTAAGTCCGAGCT

Table S5. Genotyping primers used to generate KO clones, Related to Figures 2,4 and 5.

Gene	Fw primer	Rv primer
Tcf7l1	AGCCATTTTGACGTCTGTCC	CCGAGAGCTCCTGTCAGAAC
Apc	CGTCAGTGCAGTGTTCCTTC	AGTCTGAAGTCAGCCCAGGA
Nprl2	CAAAGTAGACCACTGGGTGGA	AGAAGAAGCTGATTGGCTGC
Depdc5	ACTCTCAGGGAAAAGGCAGA	TGCTTTTGCAAGTCAAGTCG
Tsc1	GGGGATAGGGATAGGGGTCT	ATGAACTGCAGGGTTTCTGG
Tsc2	GACAGGAGGCAAGCAGAAAC	GCTAGAGAAGGGCAGGGAGT
Rictor	ACGGTGGGACAGAAACTCAG	TCAAGCAGTTTCAGTGCCAC

Table S6. gRNAs used for lentiviral CRISPR-mediated gene knockout, Related to Figures 1-3.

Gene	gRNA	Addgene ID
Tcf7l1-g1	TTACGGTGCGCGCTCCACC	105015
Tcf7l1-g2	GCTGTCTTTGGGTCGATCTC	105016
Kcmf1-g1	TGAGTTACGATGAATCGAG	105017
Pggt1b-g1	GGTCAAATCTGAGTCGCTG	105018
Vps39-g1	CTGTGGTGCTTAACGAGGA	105019
Znrf1-g1	TTGTACTCATCGGTTCCAC	105020
Zfp219-g1	ACGGGGCACGCATGATCAA	105021
Apc-g1	GTATTGTTGGGAAATTCCCG	105022
Nelfb-g1	AAGACCCGGCGCCAAGGAG	105023
Nelfb-g2	GGATCTACACGGGACACCG	105024
Nelfcd-g1	GATCAAGCACTTCGACCCT	105025
Nelfcd-g2	TCTGATGCGGGGTACCAAG	105026
Stat3	ATCGCTTACTCTCCGCATC	105027
Hgs-g1	ATCTGCGACCTGATCCGTC	105032
Vps25-g1	CAATCTGAATTGACTCCAC	105033
Ptpn23-g1	CTAAAGCGCATCCTCGCCA	105034
Pou5f1-g1	TCGTATGCGGGCGGACATG	105035
Pou5f1-g2	CTAGTCCCCCAAGTTGGCG	105036
Nprl2-g1	TCGCGAGAACTGTTTGACA	105037
Nprl2-g2	CGGCCAGCTTTTTAACGAT	105038
Tsc2-g1	CTCATACACTCGAGTGGCG	105039
Tsc2-g2	CAATCGCATCCGAATGATA	105040
Rictor-g1	TAGCGCAGCGCTCGCAACC	105041
Rictor-g2	GACATTCAGCAGAGCAACG	105042

Table S7. Primers used for the RT-qPCR assay, Related to Figure 4.

Gene	Fw primer	Rv primer
Zfp42	CCCTCGACAGACTGACCCTAA	TCGGGGCTAATCTCACTTTCAT
Nanog	TCTTCCTGGTCCCCACAGTTT	GCAAGAATAGTTCTCGGGATGAA
Esrrb	GCACCTGGGCTCTAGTTGC	TACAGTCCTCGTAGCTCTTGC
Tbx3	AGATCCGGTTATCCCTGGGAC	CAGCAGCCCCCACTAACTG
Klf4	GTGCCCCGACTAACCGTTG	GTCGTTGAACTCCTCGGTCT
Tfcp2l1	CAGCCCGAACACTACAACCAG	CAGCCGGATTTCATACGACTG
Fgf5	AAGTAGCGCGACGTTTTCTTC	CTGGAAACTGCTATGTTCCGAG
Otx2	TATCTAAAGCAACCGCCTTACG	AAGTCCATACCCGAAGTGGTC

Deposited datasets

1. RNA-seq analyses. Rex1GFP+ population in wildtype, Nprl2 KO and Tsc2 KO mESCs and differentiation timecourse (day 0-3) of wildtype Rex1GFP mESCs. Gene Expression Omnibus: GSE107060

2. CRISPR-KO screen. Raw readcounts and all MAGeCK outputs of self-renewal and differentiation. BioStudies: S-BSST61
