# Supplementary material for: Genome-wide CRISPR-KO Screen Uncovers mTORC1-Mediated Gsk3 Regulation in Naive Pluripotency Maintenance and Dissolution
Source: Cell Rep. 2018 Jul 11;24(2):489–502. doi: 10.1016/j.celrep.2018.06.027 (PMC6057492; doi:10.1016/j.celrep.2018.06.027)
Supplement: Document S2. Article plus Supplemental Information [file mmc4.pdf]

Resource

Cell Reports

Genome-wide CRISPR-KO Screen Uncovers mTORC1-Mediated Gsk3 Regulation in Naive Pluripotency Maintenance and Dissolution

Graphical Abstract

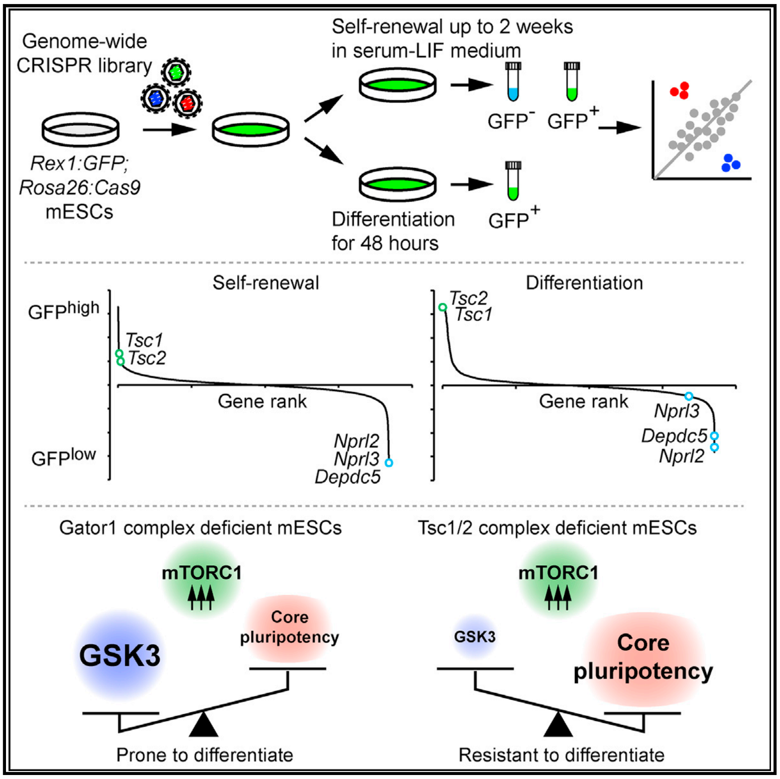

Authors

Meng Li, Jason S.L. Yu, Katarzyna Tilgner, Swee Hoe Ong, Hiroko Koike-Yusa, Kosuke Yusa

Correspondence

ky1@sanger.ac.uk

In Brief

Li et al. conducted genome-wide CRISPR screens in mouse ESCs to identify genes affecting maintenance of and exit from naive pluripotency using a Rex1GFP reporter. They show that loss of two mTORC1-negative regulators, Tsc1/2 and Gator1, can cause opposing phenotypes through differential regulation of Gsk3 activity.

Highlights

- Genome-wide CRISPR screening identifies naive pluripotency regulators in mouse ESCs
- mTORC1-negative regulators from two axes show opposing phenotypes
- Gator1 is required for proper self-renewal and differentiation via Gsk3 regulation
- Tsc2 loss causes Akt-dependent, mTORC1-dependent Gsk3 suppression

Data and Software Availability

GSE107060

Li et al., 2018, Cell Reports *24*, 489–502
July 10, 2018 © 2018 The Author(s).
https://doi.org/10.1016/j.celrep.2018.06.027

OPEN ACCESS
CellPress

Genome-wide CRISPR-KO Screen Uncovers mTORC1-Mediated Gsk3 Regulation in Naive Pluripotency Maintenance and Dissolution

Meng Li,[1] Jason S.L. Yu,[1] Katarzyna Tilgner,[1] Swee Hoe Ong,[1] Hiroko Koike-Yusa,[1] and Kosuke Yusa[1,2,*]
[1]Wellcome Sanger Institute, Hinxton, Cambridge CB10 1SA, UK
[2]Lead Contact
*Correspondence: ky1@sanger.ac.uk
https://doi.org/10.1016/j.celrep.2018.06.027

SUMMARY

The genetic basis of naive pluripotency maintenance and loss is a central question in embryonic stem cell biology. Here, we deploy CRISPR-knockout-based screens in mouse embryonic stem cells to interrogate this question through a genome-wide, non-biased approach using the Rex1GFP reporter as a phenotypic readout. This highly sensitive and efficient method identified genes in diverse biological processes and pathways. We uncovered a key role for negative regulators of mTORC1 in maintenance and exit from naive pluripotency and provided an integrated account of how mTORC1 activity influences naive pluripotency through Gsk3. Our study therefore reinforces Gsk3 as the central node and provides a comprehensive, data-rich resource that will improve our understanding of mechanisms regulating pluripotency and stimulate avenues for further mechanistic studies.

INTRODUCTION

Mouse embryonic stem cells (mESCs) are derived from the inner cell mass of blastocyst-stage embryos and can be indefinitely propagated while maintaining the ability to differentiate into all three germ layers. They have served not only as a platform for genome manipulation and production of transgenic mice but also as an essential model system to study the molecular mechanisms of self-renewal and differentiation. In particular, mechanisms that underpin the maintenance of pluripotency have been the subject of intense research, establishing the framework through which the pluripotent state is regulated by intrinsic and extrinsic factors. Intrinsically, the core transcription factors Pou5f1, Sox2, and Nanog act together with accessory factors Esrrb, Klf2, and Tfcp2l1 to consolidate the pluripotent identity (Hackett and Surani, 2014). Extrinsically, leukemia inhibitory factor (LIF)-STAT3 signaling plays a key role to sustain pluripotency (Ohtsuka et al., 2015) and Wnt signaling cooperates to suppress differentiation (Sato et al., 2004), whereas FGF-MAPK signaling is essential for mESCs to initiate differentiation (Kunath et al., 2007). In uncovering these basic principles, culture conditions that permit the preservation of pluripotency via dual inhibition of MEK and Gsk3 kinases (termed 2i) were established (Ying et al., 2008). mESCs cultured with 2i closely resemble epiblasts in pre-implantation embryos, sharing transcriptomic and epigenomic features that reflect the ground or naive state of pluripotency (Leitch et al., 2013; Marks et al., 2012).

As the mechanisms of pluripotency maintenance have become clearer, research focus has shifted toward understanding how the exit from pluripotency and initiation of lineage specification are achieved. In response to differentiation cues, mESCs must resolve the naive pluripotency network and initiate transcriptional events that drive the progression through an intermediate or formative state to the primed state (Smith, 2017). One key factor regulating this process is Tcf7l1, which is a transcriptional suppressor and colocalizes with Pou5l1 and Sox2, thereby counteracting their transcriptional activation and suppressing the intrinsic pluripotency program (Cole et al., 2008). Conversely, loss of Tcf7l1 resulted in upregulation of Nanog, severely delaying the onset of differentiation (Pereira et al., 2006). The activity of Tcf7l1 is subject to regulation by Wnt signaling and thus depends on Gsk3 activity. Inhibition of Gsk3 results in the nuclear translocation of β-catenin, which upon binding to Tcf7l1, abrogates its suppressor activity (Wray et al., 2011; Yi et al., 2011). This is a clear example of how extrinsic signaling dictates the dissolution of the core pluripotency network. Although reverse and forward genetic approaches have been successful in identifying such factors (Betschinger et al., 2013; Guo et al., 2011; Kaji et al., 2006; Leeb et al., 2014; Pereira et al., 2006), the full repertoire of genes and pathways involved in this process remains elusive.

The CRISPR-Cas system is the defense machinery found in a range of bacterial and archaea species (Makarova et al., 2015). Among them, the CRISPR-Cas9 system derived from *Streptococcus pyogenes* is most extensively characterized (Jinek et al., 2012, 2014; Nishimasu et al., 2014; Sternberg et al., 2014) and has been adapted into versatile genetic tools (Adli, 2018). The key advantage of the CRISPR-Cas9 system is the high consistency and efficiency in generating targeted gene knockouts, which has enabled us and others to carry out genome-scale CRISPR-knockout (KO) screening in mammalian cells (Koike-Yusa et al., 2014; Shalem et al., 2014; Wang et al., 2014). CRISPR-KO screening has shown superior detection sensitivity compared to RNAi screens (Evers et al., 2016), and its resolving power is evident in the unraveling of genetic

This is an open access article under the CC BY license (http://creativecommons.org/licenses/by/4.0/).

dependencies in cancer cells (Hart et al., 2015; Tzelepis et al., 2016; Wang et al., 2017).

Here we performed CRISPR-KO phenotypic screens to gain more in-depth insight and comprehensive understanding of the maintenance of and exit from naive pluripotency. The unbiased nature of CRISPR-KO screening revealed multiple genes and protein complexes whose functions have not previously been associated with pluripotency maintenance and/or differentiation. In particular, our screen revealed that regulation of Gsk3 activity is a key requirement in initiating differentiation. In addition, regulation of Gsk3 is mediated by Akt/mTOR signaling, subsequently linking nutrient and energy metabolism pathways to the exit from naive pluripotency. Our study therefore represents the most comprehensive account of the factors involved in the regulation of naive pluripotency, providing a key resource for further experimental interrogation.

RESULTS

CRISPR-KO Self-Renewal Screen Identifies Genes Regulating Naive Pluripotency

We previously performed a cell-essentiality screen in JM8 mESCs and identified 1,680 genes as essential for survival and proliferation (Tzelepis et al., 2016). However, because the phenotypic readout was proliferation, we could not distinguish factors that positively or negatively affect pluripotency maintenance from those affecting cell survival and/or proliferation. In this study, we therefore redesigned a screen using a Rex1GFP reporter (Wray et al., 2011) as a phenotypic readout. *Rex1* (also known as *Zfp42*) expression is strictly restricted to the naive pluripotent state, and its pattern reflects a heterogeneity typically observed in mESCs cultured in the serum + LIF (SL) condition (Chambers et al., 2007). Upon differentiation, *Rex1* is rapidly downregulated, allowing the near real-time readout of the pluripotent state. However, caution is required when interpreting Rex1GFP phenotype, because non-related mechanisms can influence the GFP expression level. Because genes required for cell survival and/or proliferation in the 2i + LIF (2iL) condition have not been investigated and may differ from those required in the SL condition, we sought to perform a cell-essentiality screen in 2iL in parallel. We generated Rex1GFP-Cas9 mESCs by introducing the *Cas9* gene into the *Rosa26* locus (Figure S1) and used this line as wild-type mESCs throughout this study.

Figure 1A outlines our screening strategy. Rex1GFP-Cas9 mESCs were mutagenized with the v2 mouse genome-wide guide RNA (gRNA) library (Tzelepis et al., 2016). On day 2, transduced cells were collected by cell sorting and cultured in either the 2iL or the SL condition. On days 8 and 15 post-transduction, the GFP+ and GFP− populations were collected by sorting for the cells in SL, whereas cells in 2iL were simply collected without sorting. Subsequently, gRNA abundance was analyzed and statistical analyses were performed as detailed later. In all analyses, we computed depletion-enrichment (DE) scores to show contiguous negative-to-positive statistical values (see Experimental Procedures) (Tables S1 and S2).

First, we performed statistical analysis comparing GFP+ and GFP− fractions from the SL condition. This identifies genes that affect the ratio between GFP+ and GFP− populations and thus are most likely to affect self-renewal. From positive selection, we identified 27 and 37 genes whose KO increased the GFP+ fraction on days 8 and 15, respectively, at a cutoff of the false discovery rate (FDR) of 10% (Figures 1B and 1C). Consistent with its established function, *Tcf7l1* was identified as a gene restricting the GFP+ fraction at both time points. We validated 5 genes identified in the day 15 dataset (*Vps39*, *Pggt1b*, *Znrf3*, *Kcmf1*, and *Zfp219*) that have not been previously linked to pluripotency maintenance (Figure 1D). We also identified 129 and 69 genes that, when knocked out, decreased the GFP+ fraction on days 8 and 15, respectively, from negative selection (Figures 1B and 1C). The genes on day 15 included accessory factors such as *Ctnnb1*, *Klf4*, and *Esrrb*.

We then performed additional statistical analysis by comparing the read counts between the library plasmid and the GFP+ or GFP− fractions. This comparison identifies genes that affect mutant representation during the course of screen (i.e., cell survival and/or proliferation). By comparing resulting DE scores between GFP+ and GFP− populations, the kinetics of genes affecting naive pluripotency maintenance can be captured (Figure S2). For instance, genes that exhibit rapid loss of pluripotency upon KO, such as *Pou5f1* and *Sox2*, had been already depleted from both GFP+ and GFP− populations by day 8, but genes in the LIF-Stat3 pathway showed depletion initially from the GFP+ population and then from the entire population, permitting direct observation of the differentiation trajectory (Figures S2G and S2H). We identified two subunits (Nelfb and Nelfcd) of the negative elongation factor complex, showing a trajectory similar to the LIF-Stat3 pathway genes (Figure 1E). Through individual gRNA experiments, we confirmed that gene inactivation resulted in gradual loss of naive pluripotency and eventual depletion from the entire culture (Figure 1F), confirming that our data can accurately capture the loss of the naive state across time.

Lastly, we compared genes essential for survival and/or proliferation between the GFP+ cells in SL and the cells in 2iL and found considerably different requirements to maintain proliferation in these conditions (Figure 1G). For example, *Sall4* is required for self-renewal in the SL condition, but not in the 2iL condition. Loss of *Tfcp2l1* and Gator1 complex genes (*Nprl2*, *Nprl3*, and *Depdc5*) showed a more pronounced effect in the 2iL condition than in the SL condition. Genes specifically required for cells in 2iL were enriched in metabolic and biosynthesis processes, which are likely to be a response to absence of serum constituents, and insulin signaling (Figure 1H). Altogether, these data uncover several genes not previously connected to naive pluripotency maintenance, highlighting the value of our genome-wide loss-of-function screens.

CRISPR-KO Differentiation Screen Identifies Genes that Impede or Accelerate Pluripotency Exit

Next, we performed a CRISPR-KO screen to identify genes required for proper initiation of differentiation using the Rex1GFP reporter (Figure 2A; Figure S3). We compared gRNA abundance of the GFP+ fraction to that of the unsorted population and identified 563 genes (FDR 10% cutoff for positive selection) required for proper exit from pluripotency (Figure 2B)

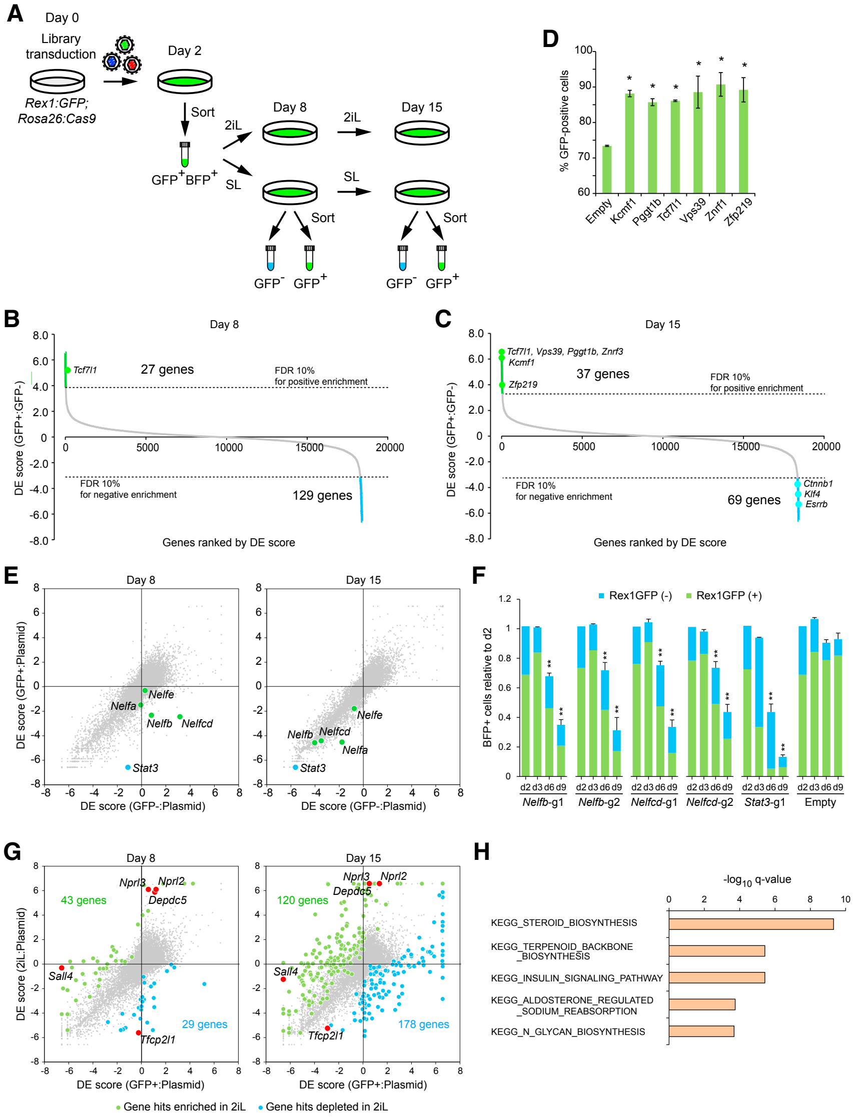

Figure 1. CRISPR-KO Screen in Maintenance of Naive Pluripotency

(A) Screening strategy for maintenance of naive pluripotency. Lentivirus used expresses blue fluorescent protein (BFP), and transduced cells were thus enriched on day 2 by sorting. For mESCs in SL, gRNA abundance in sorted GFP+ and GFP− populations was analyzed.

(B and C) Screen summaries as ranked DE score plots for day 8 (B) and day 15 (C) by comparing GFP+ and GFP− populations.

(D) Validation of newly identified genes.

(E and F) Differentiation trajectory (Figure S2) identified potential involvement of the negative elongation factor in naive pluripotency maintenance. (E) Validation experiment was performed with 2 gRNAs each for *Nelfb* and *Nelfcd*, together with a gRNA targeting *Stat3* as a positive control (F).

(G) Comparison of the screen results between GFP+ cells in SL and the cells in 2iL. Green and blue dots indicate genes enriched or depleted in cells in 2iL.

(H) GO terms overrepresented in processes specifically required in mESCs cultured in 2iL.

Data are shown as mean ± SD. (D and F) n = 3. Student's t test was performed. $^{*}p \leq 0.05$. See also Figures S1 and S2.

(Tables S1 and S2). The two positive control genes, *Tcf7l1* and *Apc*, were identified among the hits. We were also able to identify 12 genes whose loss accelerated differentiation with a relaxed cutoff of FDR of 25% (Figure 2B). To confirm the validity of our result, we performed gene set enrichment analysis (GSEA) using a control gene set including 28 genes identified by an RNAi screen performed in a similar experimental setting (Betschinger et al., 2013). This gene set showed strong enrichment in our screen, indicating high concordance (Figure 2C). We also performed GSEA using genes identified in our self-renewal screen (Figure 1C) and observed a positive correlation with the differentiation screen; genes that increased the GFP+ fraction in the SL condition showed higher retention of Rex1GFP expression during differentiation, and vice versa (Figures 2D and 2E). This indicates that gene hits in self-renewal in the SL condition most likely show the same effect in the differentiation condition. However, genome-wide comparison of the self-renewal and differentiation screens revealed that most genes showing higher GFP retention upon differentiation did not influence the GFP+:GFP− ratio in the SL condition (Figure 2F). These results suggest that there are at least 2 distinct classes of genes regulating the maintenance of and/or exit from naive pluripotency.

Pathways Involved in the Exit from Pluripotency Are Diverse

To gain a comprehensive picture of genes involved in naive pluripotency exit, we performed GSEA using the entire Reactome and KEGG gene sets and identified known signaling pathways such as fibroblast growth factor (FGF)-mitogen-activated protein kinase (MAPK), Wnt, and phosphatidylinositol 3-kinase (PI3K) pathways, mRNA degradation, and microRNA (miRNA) biogenesis pathways (Table S3). The remaining processes are relatively less studied in the context of mESC differentiation. For example, mitochondrial genes showed the strongest enrichment; nearly a half of the 563 gene hits were mitochondrial genes. In addition, glycolysis was identified in GSEA. Although it is known that naive pluripotent cells show higher mitochondrial activity than cells with primed pluripotency (Zhou et al., 2012), how ATP production affects the onset of differentiation remains elusive. In addition, genes involved in endosome and vesicle trafficking were enriched, but their involvement is not well understood.

We have summarized our findings by grouping our gene hits in the context of signaling, protein complexes, and other functional

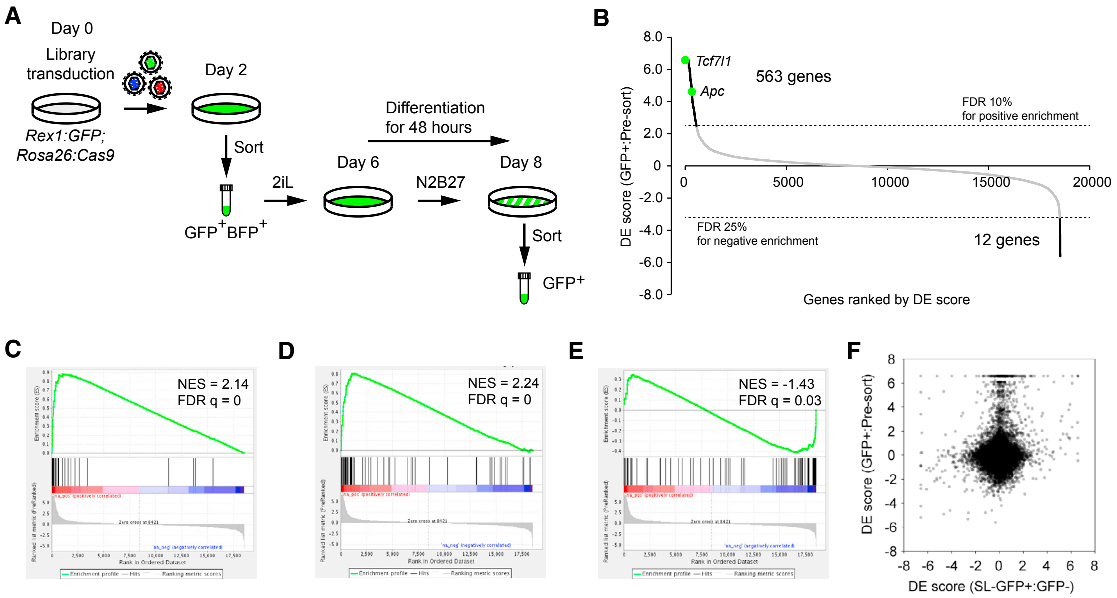

Figure 2. CRISPR-KO Screen in Exit from Naive Pluripotency

(A) Screening strategy for exit from naive pluripotency.

(B) Screen summary shown as a ranked DE score plot.

(C–E) GSEA for a gene set identified by a siRNA screen (Betschinger et al., 2013) (C) and a set of genes identified in positive (D) and negative (E) selection from our self-renewal screen (GFP+:GFP−) on day 15. NES, normalized enrichment score.

(F) Comparison of DE scores between self-renewal (day 15) and differentiation screens. Although there are correlations as observed in (D) and (E), most genes identified in exit from pluripotency do not have a major impact on Rex1GFP heterogeneity in maintenance culture.

See also Figures S1 and S3.

categories (Figure 3). In the signaling category, genes involved in FGF-MAPK, LIF-STAT, PI3K-AKT, and Wnt pathways were identified. mRNA degradation pathways such as non-sense-mediated decay (Li et al., 2015) and the m^6-A RNA methylation enzyme complex (Batista et al., 2014; Geula et al., 2015) have been previously described. miRNAs are also known to regulate differentiation (Kanellopoulou et al., 2005; Sinkkonen et al., 2008). In the nucleus, several chromatin-modifying and chromatin-remodeling complexes were identified (Cruz-Molina et al., 2017; Kaji et al., 2006; Whyte et al., 2012). *Pou5f1* was identified as a gene required for differentiation. Although complete loss of Pou5f1 leads to differentiation (Niwa et al., 2000), Pou5f1 is also known to have roles in lineage specification (Wang et al., 2012; Yang et al., 2014). It has also been shown that *Pou5f1*$^{+/-}$ ESCs show enhanced self-renewal capability and resistance to differentiation (Karwacki-Neisius et al., 2013). Because double-stranded break (DSB)-mediated genome editing generates various alleles, our mutant library must have contained heterozygously edited cells, which correspondingly showed a delayed differentiation phenotype.

In addition to known genes and pathways, we identified other pathways not previously connected to naive pluripotency exit. For instance, genes in the heparan sulfate biosynthesis pathway were identified. As a known positive regulator of FGF signaling (Ornitz, 2000), the deficiency likely results in weakened FGF-MAPK signaling. We also identified multiple genes involved in vesicle trafficking and endocytosis. Of the various complexes identified, all 6 genes that compose the homotypic fusion and protein sorting (HOPS) complex showed strong differentiation defects, and one of the genes, *Vps39*, was identified in the self-renewal screen as a factor that decreases heterogeneity (Figures 1C and 1D). We validated some factors by individual gRNA experiments (Figure S4), showing the accuracy of our screen results. Altogether, these findings indicate that our screen both confirmed and added to genes known to participate in naive pluripotency exit. Our screen therefore provides a comprehensive dataset for better understanding the molecular mechanisms underlying exit from naive pluripotency.

Increasing mTORC1 Activity through Gator1 or Tsc1/2 Loss Results in Opposing Phenotypes

The two mTOR-containing complexes, namely, mTORC1 and mTORC2, are crucial mediators or regulators of the PI3K-Akt pathway in response to external growth stimuli and involved in multiple processes such as translation regulation, energy metabolism, autophagy, and development (Saxton and Sabatini, 2017). mTORC1 activity is also regulated by amino acid sensing (Wolfson and Sabatini, 2017). In the previous small interfering RNA (siRNA) screen, mTORC1 regulators such as Tsc1/2, RagA/C, folliculin, and the Lamtor complex were identified (Betschinger et al., 2013). Our screen identified additional factors involved in mTORC1 regulation and thus further connects the mTOR network to pluripotency regulation (Figure 3). These factors include the Gator1, Gator2, and Kicstor complexes, as well as Stk11. At a relaxed cutoff, we were able to identify Rictor, an

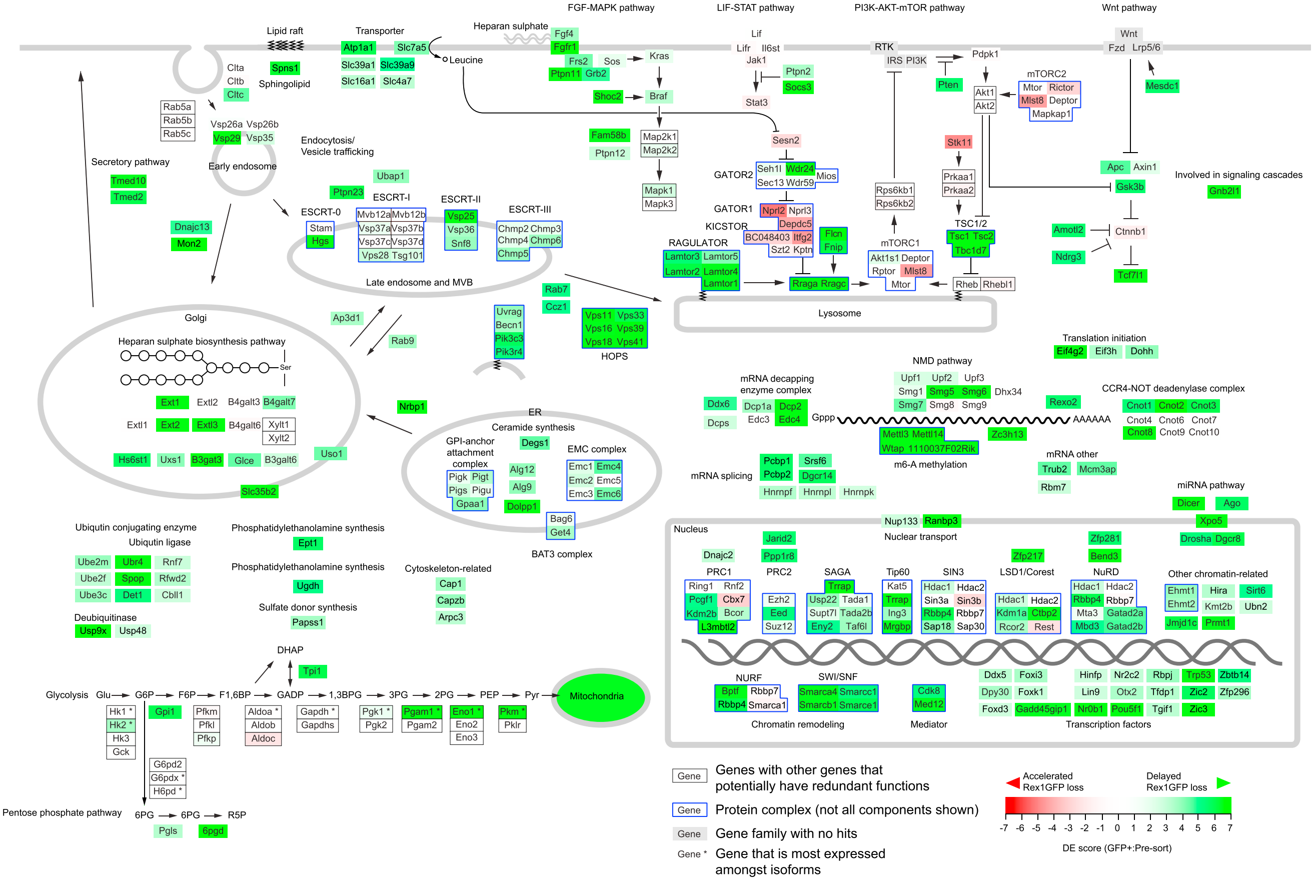

Figure 3. Genes Identified in the CRISPR-KO Screen for Exit from Naive Pluripotency

Genes with known functions are placed in pathways, protein complexes, or cellular compartments. When genes with redundant function are present, these genes are boxed in black. Defined protein complexes are boxed in blue. Not all components are shown for protein complexes. See also Figure S4.

essential component of the mTORC2 complex. We also identified Mlst8, a factor that was identified in both mTORC1 and mTORC2 complexes but that is specifically required for mTORC2 function (Guertin et al., 2006). With the exception of Wdr24, these genes, when perturbed, demonstrated accelerated differentiation.

The Tsc1/2 complex functions as guanosine triphosphatase (GTPase)-activating protein (GAP) toward Rheb (Inoki et al., 2003) (Figure 4A). Genetic deletion of the Tsc1/2 complex leaves Rheb in a guanosine triphosphate (GTP)-bound active form, resulting in constitutive activation of mTORC1 (Zhang et al., 2003). The Gator1 complex also negatively regulates mTORC1 activity through its GAP activity toward RagA in response to amino acid sensing (Bar-Peled et al., 2013) (Figure 4A). Therefore, Tsc1/2 and Gator1 complexes negatively regulate mTORC1 via two distinct signaling cascades. However, in both self-renewal and differentiation screens, these two complexes showed opposing phenotypes. During self-renewal, Gator1 complex KO increased heterogeneity, while Tsc1/2 KO acted to preserve homogeneity (Figure 4B). Under differentiation conditions, Tsc1/2 deficiency resulted in strong resistance to differentiation, whereas Gator1 KO accelerated differentiation (Figure 4C). Because both mTORC1 regulators are less understood in the context of mESC self-renewal and differentiation, we sought to carry out further molecular studies on these hits.

Gator1 Depletion Diminishes Self-Renewal and Promotes Differentiation

First, we generated *Nprl2* and *Depdc5* KO mESCs in the Rex1GFP background (Figures S5A and S5B). These KO mESCs showed indistinguishable morphology from wild-type cells and, as expected, upregulation of mTORC1 activity (Figure S5E). To investigate their self-renewal capability, we analyzed the percentage of cells retaining Rex1GFP expression after sorting GFP+ cells. In the SL condition, the GFP+ fraction in wild-type cells decreased for the first 4 days and plateaued around 70%, whereas *Tcf7l1* KO mESCs maintained a higher GFP+ fraction around 90% (Figure 4D, left panel). All *Nprl2* or *Depdc5* KO clones showed kinetics similar to wild-type cells but plateaued at a GFP+ fraction of 40%–50%, significantly lower than that of wild-type cells (Figure 4D, left panel). In the 2iL condition, there was no difference between wild-type and *Tcf7l1* KO mESCs; however, both *Nprl2* and *Depdc5* KO mESCs lost 5%–10% of the GFP+ fraction by day 6 (Figure 4D, right panel). We also investigated reactivation of *Rex1* expression by culturing sorted GFP− cells. In wild-type and *Tcf7l1* KO mESCs, approximately

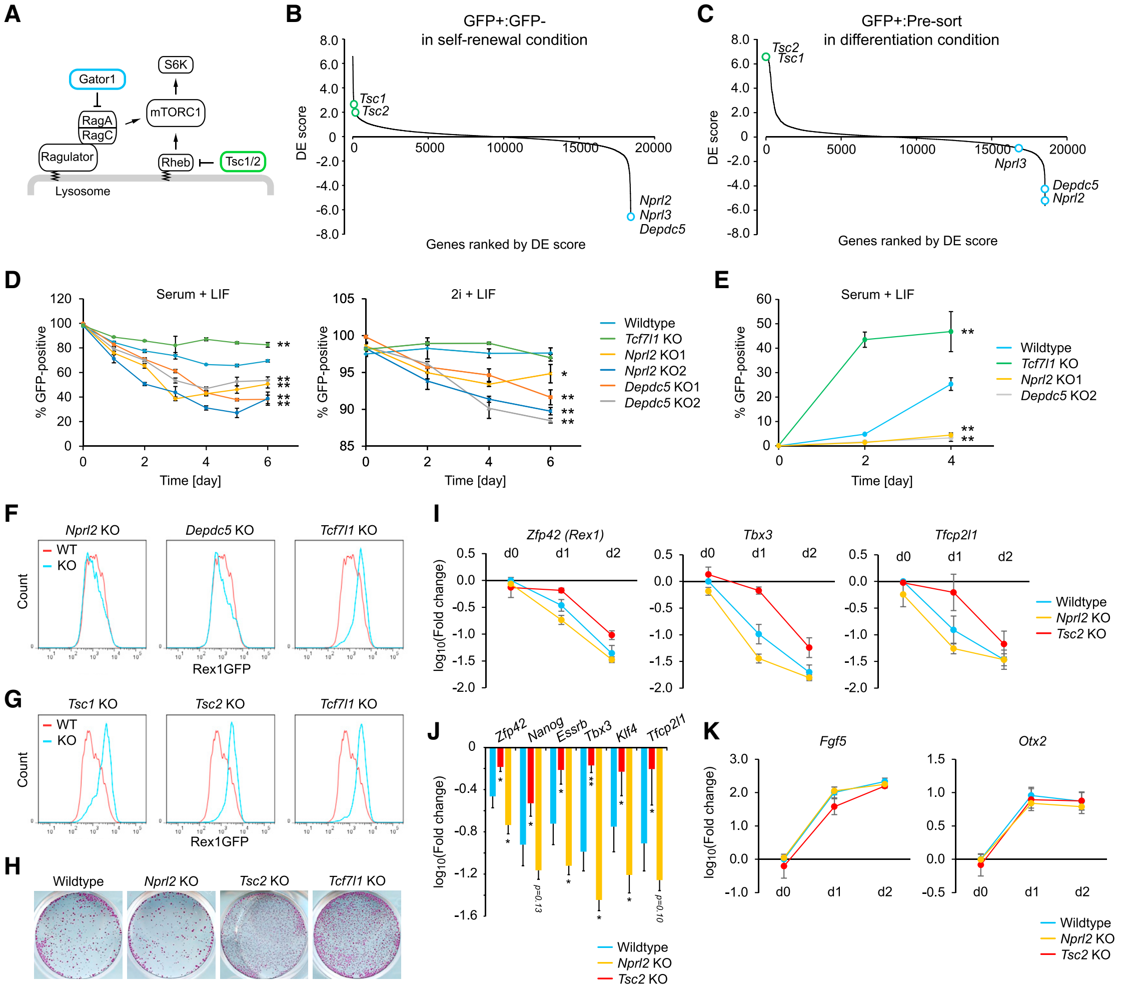

Figure 4. Gator1 and Tsc2 Loss Exhibit Opposing Phenotype on Naive Pluripotency Network Resolution

(A) Schematic of mTORC1 regulators.

(B and C) Ranked DE score plots from the self-renewal (B) and differentiation (C) screens, highlighting opposing phenotypes between Tsc1/2 and Gator1.

(D) Maintenance of naive pluripotency measured as a percentage of Rex1GFP+ cells in the SL condition (left panel) and the 2iL condition (right panel).

(E) Reacquisition of naive pluripotency.

(F and G) RexGFP profiles of indicated KO mESCs after 27 hr differentiation for Gator1 (F) and Tsc1/2 (G) complex. *Tcf7l1* KO mESCs were used as a positive control.

(H) Commitment assay.

(I–K) qRT-PCR analysis of differentiating wild-type, *Nprl2* KO mESCs, and *Tsc2* KO mESCs at the indicated days. Selected naive (I and J) and formative (K) markers were analyzed. Day 1 data are summarized in (J). Expression was normalized to day 0 wild-type expression, from which $\log_{10}$(fold change) were calculated.

Data are shown as mean ± SD. (D, E, and I–K) n = 3. Student's t test was performed. *p $\leq$ 0.05; **p $\leq$ 0.01. See also Figures S4 and S5.

20% and 50%, respectively, reactivated GFP expression by day 4, but reactivation in both *Nprl2* and *Depdc5* KO mESCs was far less efficient, resulting in only 2%–3% (Figure 4E). These results validate the Gator1 KO phenotype observed in our self-renewal screen and indicate that self-renewal capability is partially compromised in Gator1 KO mESCs.

Tsc1/2 Depletion Delays Differentiation and Reinforces Naive Pluripotency

To analyze differentiation phenotype of the two mTORC1-negative regulators, we also generated *Tsc1* and *Tsc2* KO mESCs and confirmed expected mTORC1 upregulation (Figures S4C–S4E). We analyzed GFP profiles on day 1 of differentiation. All phenotypes observed in individual KO mESCs were in agreement with our screening results: Gator1 KO mESCs lost Rex1GFP faster than wild-type, whereas Tsc1/2 KO mESCs failed to initiate differentiation (Figures 4F and 4G). To test whether the Rex1GFP profiles correlated with cellular lineage commitment, we reseeded cells into the 2iL medium after 24 hr differentiation. We found that the number of alkaline phosphatase-positive colonies correlated with the Rex1GFP profiles (Figure 4H). To confirm, we analyzed the expression level of key

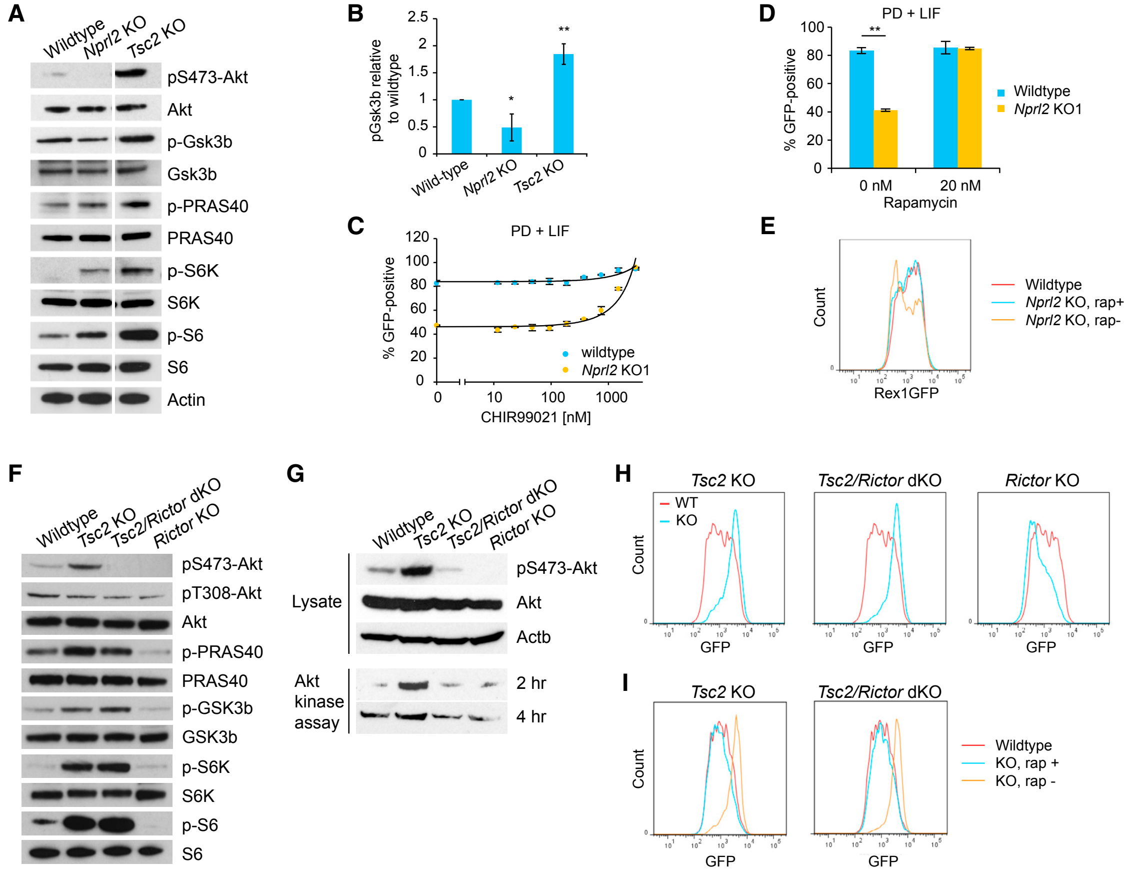

Figure 5. Gsk3 Is Differentially Affected by mTORC1 Upregulation upon *Nprl2* and *Tsc2* Loss

(A) Western blot analysis of key phosphorylation sites in the Akt-mTORC1 pathway.
(B) Quantification of the phospho-Gsk3β level.
(C) Percentage of Rex1GFP+ cells in response to reducing the dose of CHIR992021 in wild-type and *Nprl2* KO mESCs.
(D) Restabilization of naive pluripotency by rapamycin in *Nprl2* KO mESCs.
(E) Restoration of differentiation in *Nprl2* KO mESCs by rapamycin.
(F) Phosphorylation profile in *Tsc2* KO mESCs with or without *Rictor* KO.
(G) Akt kinase assay.
(H) Rex1GFP profile of indicated KO mESCs after 27 hr differentiation.
(I) Full restoration of differentiation in both *Tsc2* sKO and *Tsc2/Rictor* dKO mESCs by rapamycin.
Data are shown as mean ± SD. (B and D) n = 3. Student's t test was performed. *$p \leq 0.05$; **$p \leq 0.01$. See also Figures S4 and S5.

naive and formative-stage markers up to day 2. All naive markers showed significant delay in downregulation in *Tsc2* KO mESCs, whereas 4 of the 6 markers tested showed significant accelerated downregulation at day 1 in *Nrpl2* KO mESCs (Figures 4I and 4J). However, upregulation of 2 formative-stage makers (*Fgf5* and *Otx2*) was not significantly affected (Figure 4K), although *Fgf5* upregulation is slightly weaker in *Tsc2* KO mESCs at day 1. We thus confirmed the effect of both mTORC1-negative regulators on naive pluripotency by individual gene targeting.

Gsk3 Is Differentially Regulated in *Nprl2* and *Tsc2* KO mESCs

mTORC1 upregulation and resulting S6K activation are known to induce the negative feedback loop and attenuate Akt activation (Zhang et al., 2006). Gsk3 is a direct downstream target of Akt and plays a central role in self-renewal and differentiation (Martello et al., 2012; Wray et al., 2011). We therefore investigated phosphorylation status of key proteins in the Akt-mTORC1 pathway. Unexpectedly, while phosphorylation on Akt-S473 was abolished as a result of negative feedback in *Nprl2* KO mESCs, *Tsc2* KO mESCs showed substantial upregulation of the phosphorylation (Figure 5A). Mirroring this pattern, Gsk3β-S9 phosphorylation was significantly downregulated in *Nprl2* KO mESCs but increased in *Tsc2* KO mESCs (Figures 5A and 5B). Another Akt target PRAS40 (also known as Atk1s1) was upregulated in both KO mESCs (Figure 5A). Altogether, both KO mESCs showed expected mTORC1/S6K upregulation but seemingly divergent phosphorylation patterns on Akt and its downstream targets.

Increased Gsk3 Activity Destabilizes Naive Pluripotency in *Nprl2* KO mESCs

Given that Gsk3 plays a pivotal role in regulating naive pluripotency, we hypothesized that phenotypic discrepancy between *Tsc2* and *Nprl2* KO mESCs is mediated by the difference in Gsk3 regulation. To further investigate the effect of Gsk3 activity in *Nprl2* KO mESCs, we seeded GFP+ *Nprl2* KO mESCs into N2B27+1iL (MEKi + LIF) medium supplemented with serially diluted Gsk3 inhibitor and measured the percentage of GFP+ cells after 3 days. Although 80% of wild-type cells could maintain Rex1GFP expression even in the absence of the GSK3 inhibitor, *Nprl2* KO mESCs were more sensitive to the GSK3 inhibitor dose and more than 50% of the cells lost GFP expression in the absence of the inhibitor (Figure 5C). This result clearly indicates that *Nprl2* KO mESCs have elevated Gsk3 activity and thus depend more on Gsk3 inhibition to maintain Rex1GFP expression. If the negative feedback mechanism is responsible in *Nprl2* KO mESCs, mTORC1 inhibition via rapamycin should reactivate Akt and hence downregulate Gsk3, thereby rescuing phenotype in both self-renewal and differentiation. As shown in Figure 5D, rapamycin-treated *Nprl2* KO mESCs maintained Rex1GFP expression as efficiently as wild-type cells in the absence of the GSK3 inhibitor. The same treatment in differentiation conditions also rescued *Nprl2* KO phenotype, showing a Rex1GFP profile identical to that of wild-type cells (Figure 5E). Altogether, our results revealed an amino acid-sensing mediator, the Gator1 complex, as a regulator of naive pluripotency.

Increased Akt Activation in *Tsc2* KO mESCs Does Not Contribute to Phenotypic Outcome

Tsc2 KO mESCs showed expected mTORC1 activation (Figure 5A) and, as seen in *Tsc2* KO mouse embryonic fibroblasts (MEFs) (Zhang et al., 2006), upregulation of Gsk3 phosphorylation. However, in sharp contrast to *Tsc2* KO MEFs, we found that *Tsc2* KO mESCs showed substantial upregulation of phosphorylation on Akt-S473 (Figures 5A and 5F), suggesting that Akt is upregulated, rather than being attenuated. Overexpression of constitutively active Akt is known to sustain self-renewal in the absence of LIF and to be associated with increased Gsk3 phosphorylation (Bechard and Dalton, 2009; Watanabe et al., 2006). These observations raised the possibility that in *Tsc2* KO mESCs, upregulated Akt suppresses Gsk3 and sustains naive pluripotency.

To explore whether downregulation of Akt-S473 alters Gsk3 activity, we inactivated the mTORC2 complex by knocking out *Rictor*, an essential component of the mTORC2 complex (Guertin et al., 2006), in *Tsc2* KO and wild-type backgrounds (Figure S5F). Phosphorylation of Akt-S473 was abolished in both *Tsc2/Rictor* double-KO (dKO) and *Rictor* single-KO (sKO) cells, indicating that mTORC2 is fully responsible for the phosphorylation of Akt-S473 and that, unlike in *Tsc2* KO MEFs (Huang et al., 2008), mTORC2 was ectopically activated in *Tsc2* sKO mESCs. However, phosphorylation at T308 was only slightly affected in KO mESCs (Figure 5F). To confirm Akt activity, we performed a kinase assay using total Akt immunoprecipitated from the KO lines. Consistent with the S473 phosphorylation pattern, Akt from *Tsc2* sKO mESCs showed a markedly upregulated kinase activity, whereas Akt from both *Tsc2/Rictor* dKO and *Rictor* sKO mESCs showed minimal activity (Figure 5G). The lack of mTORC2 activity did not affect the mTORC1 pathway in *Tsc2/Rictor* dKO mESCs, as evident from the comparable phosphorylation levels on S6K and S6 (Figure 5F). Although *Rictor* sKO and *Tsc2/Rictor* dKO mESCs both showed minimal Akt activity, both Gsk3 and PRAS40 remained highly phosphorylated in *Tsc2/Rictor* dKO mESCs as in *Tsc2* sKO mESCs, but not in *Rictor* sKO mESCs (Figure 5F), suggesting that Gsk3 is not under the control of Akt in a *Tsc2*-deficient background. Consistent with the Gsk3 phosphorylation status, *Tsc2/Rictor* dKO mESCs showed delayed differentiation comparable to that observed in *Tsc2* sKO mESCs (Figure 5H). In contrast, *Rictor* sKO mESCs showed an accelerated differentiation phenotype, which is consistent with our screening data (Figure 3). It has been reported that in *Tsc2*-deficient MEFs, activated S6K constitutively phosphorylates Gsk3 and downregulates its kinase activity, which is reversible upon rapamycin treatment (Zhang et al., 2006). Consistent with the literature, rapamycin treatment fully rescued the delayed differentiation observed in both *Tsc2* sKO and *Tsc2/Rictor* dKO mESCs. Both KO cells showed identical differentiation kinetics to wild-type mESCs (Figure 5I). Altogether, *Tsc2* KO causes Akt activation in mESCs, but activated mTORC1/S6K plays a major role in influencing naive pluripotency through Gsk3 regulation.

Nprl2 and *Tsc2* KO Transcriptomes Reveal Differences in Naive and Formative Gene Expression

To further explore the implications of *Nprl2* and *Tsc2* KO on stem cell properties, we performed RNA sequencing (RNA-seq) analysis on both KO lines and compared them with wild-type cells (Figure 6A). To minimize the impact that arises from the different level of heterogeneity present in each KO line, we sorted GFP+ cells before RNA isolation. We detected 512 and 2,589 differentially expressed genes in *Nprl2* and *Tsc2* KO mESCs, respectively, (FDR < 0.05). We first analyzed expression of marker genes for general, naive, and formative pluripotency markers (Figure 6B). Although general pluripotency genes were similarly expressed in all genotypes, strikingly different expression patterns were observed in naive and formative pluripotency genes between the two KO mESCs. In *Tsc2* KO mESCs, naive pluripotency genes such as *Klf4* and *Esrrb* were significantly upregulated, whereas formative pluripotency marker genes such as *Fgf5* and *Dnmt3b* were downregulated. Fold differences of the differentially expressed genes in *Nprl2* KO mESCs were generally smaller than in *Tsc2* KO mESCs, but *Nprl2* KO mESCs showed a significant downregulation in the expression of naive pluripotency genes with concomitant upregulation of formative pluripotency genes. These results indicated that Gsk3 activity levels dictate the naiveness even within the Rex1GFP+ population. *Nprl2* KO mESCs can be maintained in the SL condition, but these cells are already straddling between naive and formative pluripotent states, whereas *Tsc2* KO mESCs are more shielded from extrinsic differentiation cues and the naive state is more preferentially reinforced.

Although these two mTORC1-negative regulator KOs showed opposing phenotypes in terms of self-renewal, differentiation, and gene expression for naive and formative pluripotency markers, there should be common transcriptomic changes that

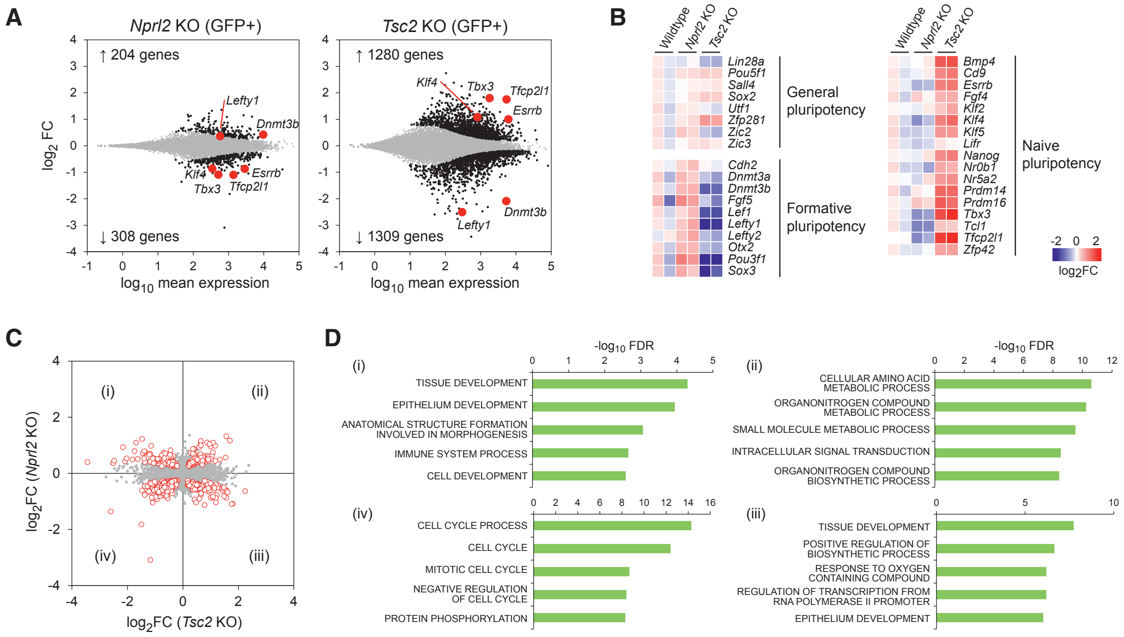

Figure 6. Transcriptome Profile in *Nprl2* and *Tsc2* KO mESCs

(A) Depletion-enrichment sequencing (DE-seq) output of differentially expressed genes in *Nprl2* and *Tsc2* KO mESCs compared to wild-type. Genes with FDR < 0.05 were highlighted with black dots, and selected pluripotency markers were highlighted in red.

(B) Expression profile of general, naive, and primed pluripotency marker genes. Primed markers were upregulated in *Nprl2* KO mESCs, while naive markers were substantially upregulated in *Tsc2* KO mESCs.

(C and D) Comparison of fold changes between *Tsc2* and *Nprl2* KO mESCs. Genes that were significantly (FDR < 0.05) up- or downregulated in either or both KO mESCs were highlighted in red. (D). Gene ontology analysis of genes highlighted in each quadrant in (C).

stem from mTORC1 upregulation. We further analyzed the RNA-seq data by comparing fold changes relative to wild-type between *Nprl2* and *Tsc2* KO mESCs (Figure 6C) and performed gene ontology (GO) overrepresentation analysis. Consistent with the preceding observation, genes that were up- or downregulated differently between the two lines were particularly enriched in development-related processes (Figures 6C and 6D, quadrants i and iii). The analysis also detected 76 commonly upregulated genes that showed enrichment in metabolic processes (Figures 6C and 6D, quadrant ii), which are potential downstream targets of mTORC1 in mESCs. Negative regulators of cell cycle were commonly downregulated in both KO mESCs (Figures 6C and 6D, quadrant iv).

DISCUSSION

More than three decades of studies on mESC have revealed several genetic and epigenetic mechanisms that regulate the stem cell-defining properties of self-renewal and pluripotency. However, a fully complete and predictive overview remains elusive. This is partly due to the lack of scalable genetic methods that allow comprehensive mapping of genes to specific phenotypes. Mammalian biology has typically been studied with resource-intensive, hypothesis-driven approaches or inefficient genome-scale screens, both of which provide a limited and context-dependent account of biological processes. Hypothesis-free forward genetics applied in yeast, *Drosophila*, and *Caenorhabditis elegans* has provided deeper insights into diverse biological processes (Forsburg, 2001; Jorgensen and Mango, 2002; St Johnston, 2002). With the advent of CRISPR-Cas9 technologies, we and others have developed a CRISPR-based loss-of-function screening approach that aims to address this central relationship in the context of mammalian systems (Koike-Yusa et al., 2014; Shalem et al., 2014; Wang et al., 2014). In the present study, we have applied CRISPR-KO screening to explore the genetic basis for naive pluripotency and provide deeper insights into the long-standing question as to how the transition from naive to lineage commitment is achieved.

Unlike previously performed CRISPR-KO screens, most of which studied cell survival and/or proliferation of cultured cancer cells (Hart et al., 2015; Tzelepis et al., 2016; Wang et al., 2017), phenotypic readout in our screens was based on reporter gene expression by fluorescence-activated cell sorting (FACS) analysis. Although a few studies have used this method and found valuable hits (Burr et al., 2017; Parnas et al., 2015), this mode of genetic screening is explored less frequently, possibly due to technical difficulties in cell sorting. These difficulties may result in loss of library complexity, which severely limits the identification of meaningful hits. After a series of optimizations for high-speed cell sorting, we routinely collect 2×10^6–5×10^6 cells per target fraction (20×–50× coverage) and use them for gRNA amplification. The two screens we performed exhibited high sensitivity, detecting true hits in both positive and negative

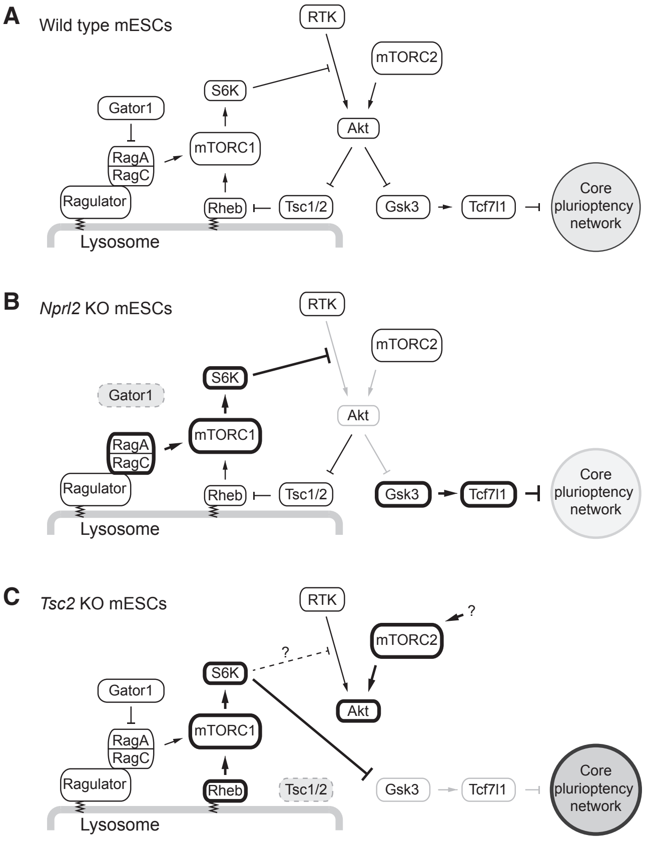

Figure 7. Models of mTORC1-Mediated Gsk3 Regulation in Each Genotype

(A) In wild-type cells, receptor tyrosine kinase (RTK)-mediated activation of Akt and the mTORC1-mediated negative feedback are in equilibrium, maintaining appropriate Gsk3 activity level.

(B) *Nprl2* loss increases mTORC1 activity and shows stronger negative feedback, resulting in Gsk3 upregulation.

(C) *Tsc2* loss also increases mTORC1 activity, but upregulated S6K directly phosphorylates and consequently inactivates Gsk3 (Zhang et al., 2006). mTORC2 is upregulated in the absence of Tsc2 protein in mESCs.

selection, including most genes known to be involved in self-renewal and/or differentiation. This high sensitivity was exemplified by *Tcf7l1*. Loss of *Tcf7l1* increased Rex1GFP+ cells by 15%, from 75% to 90%, in our experimental conditions (Figure 1C). Although this only translates to a marginal fold increase of 1.2, the gene was nonetheless detected in our self-renewal screen with a remarkable significance (FDR = 0.000707). The high-detection sensitivity is likely because of the deployment of our enhanced second-generation CRISPR-KO library (Tzelepis et al., 2016), which could potentially be further improved by using optimization metrics outlined in our recent study (Ong et al., 2017). Another approach to increase detection sensitivity would be the incorporation of the recently developed CRISPR-UMI technology (Michlits et al., 2017). This technology allows us to trace individual mutant clones. Multiple phenotype caused by heterogenous mutant alleles (as exemplified by *Pou5f1* in this study) could be more sensitively detected. Our screening method described here provides the basis for FACS-based genetic screens to the wider research community and should serve as a useful example of its deployment.

Our CRISPR-KO differentiation screen evaluating the exit from pluripotency yielded 575 gene hits (563 and 12 hits for positive and negative selection, respectively). We optimized this screen to perform positive selection to detect genes whose mutation causes sustained Rex1GFP expression. It is therefore not surprising that a smaller number of genes were detected in negative selection (i.e., genes showing accelerating differentiation). We used a relaxed cutoff (FDR of 20%) for negative selection, but *Rictor* (FDR = 0.506) could be validated by individual gRNA and in *Rictor* sKO mESCs. This suggests that although higher noise is expected, some genes under the suboptimal threshold can potentially be meaningful and worthy of further investigation. For example, Sestrin2, encoded by *Sesn2*, has been characterized as a leucine sensor, and its loss results in continuous mTORC1 activation even in the absence of leucine (Wolfson et al., 2016). *Sesn2* was not a significant hit (FDR = 0.609) but nonetheless ranked at 46 in the negative selection. Correspondingly, a leucine transporter, Slc7a5, was detected in the positive selection with an FDR of 0.0038. These again highlight the high sensitivity of CRISPR-KO screens, but there is clearly a room for further improvements to this sensitivity with regards to negative selection; it would be worthwhile to uncover more genes that show accelerated differentiation.

The previously performed screens have identified genes required for differentiation, but there has been no screen that has analyzed accelerated differentiation upon naive exit. Many genes identified from the negative selection have not been previously described in the context of pluripotency regulation and were surprisingly overrepresented with mTOR-related mediators. mTOR KO embryos exhibit post-implantation lethality at embryonic day 5.5–6.5, and mTOR KO mESCs cannot be established from KO blastocysts (Murakami et al., 2004). It has been shown that mouse blastocysts and mESCs treated with mTORC1/2 inhibitors undergo proliferation arrest with maintaining pluripotency, mimicking diapaused embryos (Bulut-Karslioglu et al., 2016). These data indicate that mTOR activity is mainly required for cell proliferation, but our present data suggest that mTOR activity influences the equilibrium of the core naive pluripotency maintenance network through the Gsk3-Tcf7l1 axis (Figure 7).

First, mTORC2 was identified from the negative selection with *Rictor* and *Mlst8*. Through *Rictor* KO mESCs, we demonstrated that mTORC2 deficiency causes a reduction of Akt activity and consequently increases Gsk3 activity. Mlst8 is a component common to both mTORC1 and mTORC2, but it has been shown that Mlst8 is essential for mTORC2, but not for mTORC1 (Guertin et al., 2006). Another finding is that mTORC1 activation by loss of mTORC1-negative regulators in the amino acid-sensing pathway (Gator1, Kicstor, and Sestrin2) leads to destabilized pluripotency maintenance (Figure 7B). We have provided genetic evidence using *Nprl2* KO cells. Because Kicstor and Sestrin2 KO cells in human cancer cells consistently showed mTORC1 upregulation (Wolfson et al., 2016, 2017), genetic disruption of these genes in mESCs would mirror the phenotype observed in Gator1 KO mESC. Therefore, our finding connects mTORC2 and the

amino acid-sensing pathway to the core pluripotency maintenance network through Akt-Gsk3-Tcf7l1.

We identified another mTORC1-negative regulator, the Tsc1/2 complex, which showed the opposite phenotype compared to Gator1 complex, namely, delayed differentiation. It has been shown that in *Tsc2* KO MEFs, Akt is inactive due to the negative feedback, but activated S6K phosphorylates Gsk3 (Zhang et al., 2006). This Gsk3 phosphorylation can be attenuated by mTORC1 downregulation (Zhang et al., 2006). Our observation in *Tsc2* KO mESCs is in agreement with this previous finding, and the observed differentiation phenotype can be explained by Gsk3 phosphorylation. Therefore, Tsc1/2 is also connected to the core pluripotency network, but the effect is opposite that of the Gator1 complex due to the rewiring of the phosphorylation network (Figure 7C).

Our observations in two mTORC1-negative regulators provide further insights into general functions of these complexes. First, Tsc1/2 complex may play a critical role in regulating Akt-Gsk3 interaction. S6K is activated in both *Tsc2* and *Nprl2* KO mESCs through mTORC1 upregulation, yet only in *Tsc2* KO cells does S6K phosphorylate Gsk3. In *Nprl2* KO mESCs, upregulated S6K seemingly causes conventional negative feedback and attenuates Akt and consequently Gsk3 phosphorylation. Therefore, Gsk3 phosphorylation by activated S6K is specific to the *Tsc2*-deficient background. Second, we unexpectedly found that Tsc2 loss resulted in the upregulation of Akt-S473 phosphorylation, indicative of mTORC2 upregulation. Rictor loss abolishes Akt-S473 phosphorylation, providing evidence for mTORC2 upregulation. It has been shown that the Tsc1/2 complex is required for proper activation of mTORC2 (Huang et al., 2008). This difference suggests that at least in mESC, Tsc2 plays a suppressor role in mTORC2 regulation. Although these might be due to the transcriptomic changes or the change in cell fate caused by gene KO, patients with Tsc1/2 or Gator1 deficiency show a different clinical phenotype (Dabora et al., 2001; Ricos et al., 2016), suggesting undiscovered roles, in addition to conventional mTORC1 regulation, that would be worthy of further investigation.

Together with the screen on self-renewal, our differentiation screen provides an invaluable resource to further understand naive pluripotency regulation and the genes required for the induction of cellular differentiation. The molecular function of some hits (e.g., the KICSTOR complex) were only recently characterized. Further interrogation of the data presented here can be useful not only in understanding pluripotency regulation but also in uncovering the fundamental molecular functions involved. The success of our screening approach indicates that with appropriate reporter systems, pooled CRISPR-KO screens can be a powerful approach for fueling insights into stem cell biology and can intimately dissect the molecular pathways that positively or negatively influence differentiation. Proliferation-essential genes in human ESCs have been characterized by genome-wide CRISPR screening (Yilmaz et al., 2018). Cellular differentiation of human pluripotent stem cells has not yet been studied extensively, and such studies would facilitate better understanding in disease mechanisms and generate more efficient differentiation protocols for cell therapy. In addition, several studies have reported the successful derivation of human naive pluripotent stem cells (Takashima et al., 2014; Theunissen et al., 2014). It would be of great interest to investigate whether our findings are recapitulated in the context of human naive pluripotency, which will lead to a greater understanding of the molecular basis of differentiation and lineage commitment. As CRISPR-KO screening technology continues to be developed and improved, it would be beneficial to apply functional genomic approaches to answer such central questions in stem cell biology.

EXPERIMENTAL PROCEDURES

Cell Culture

A Rex1GFP mESC line (Wray et al., 2011), was a gift from Austin Smith and cultured on feeder cells in SL: KO-DMEM (Thermo Fisher Scientific) supplemented with 15% fetal bovine serum (Thermo Fisher Scientific), 1% GlutaMAX (Thermo Fisher Scientific), 1% nonessential amino acid (NEAA) (Thermo Fisher Scientific), 0.1 mM 2-mercaptoethanol (Sigma), and 1,000 U mL^{-1} LIF (Millipore). Where indicated, mESCs were cultured on gelatin-coated plates in 2iL medium: NDiff227 (Takara) supplemented with 1% KO serum replacement (KSR) (Thermo Fisher Scientific), 5% BSA (Thermo Fisher Scientific), 1% NEAA, 0.1 mM 2-mercaptoethanol, 1,000 U mL^{-1} LIF, 1.0 μM PD0325901 (Selleck), and 3.0 μM CHIR90021 (Selleck). Differentiation was induced in the NDif227 medium supplemented as mentioned earlier but without the 2 inhibitors and LIF.

CRISPR-KO Screen on Self-Renewal

Cells (3.2×10^7) were transduced with the mouse v2 CRISPR library (Tzelepis et al., 2016). On day 2, approximately 1.0×10^7 cells double positive for GFP and blue fluorescent protein (BFP) were collected by sorting. Half of them were cultured on feeder cells in SL, and the other half were in 2iL medium. Thirty million cells were reseeded at every passage to maintain 300× coverage. On days 8 and 15 post-transduction, cells in SL were sorted based on GFP expression and genomic DNA was isolated. Cells in 2iL were directly subjected to genomic DNA isolation.

CRISPR-KO Screen on Exit from Pluripotency

Transduction and sorting on day 2 were performed as described earlier. Sorted cells were cultured for an additional 4 days in 2iL medium. On day 6, cells were trypsinized and 45 million cells were plated on eight 15-cm dishes (10,000 cells cm^{-1}) in NDiff227 differentiation medium. After 2 days, cells were trypsinized and 20 million cells were kept as a pre-sort control. The remaining cells were used for sorting, and approximately 3 million GFP+ cells (top 2%–3%) were collected. Genomic DNA from the pre-sort and the GFP+ fraction were isolated.

Statistical Analyses

Statistical analyses of CRISPR screens were performed by MAGeCK (Li et al., 2014). Statistical tests of quantitative data were performed by Student's t test as indicated in each figure.

DATA AND SOFTWARE AVAILABILITY

The accession number for the RNA-seq data reported in this paper is GEO: GSE107060. The accession number for the CRISPR-KO screening data, including raw read counts and MAGeCK output, reported in this paper is BioStudies: S-BSST61.

SUPPLEMENTAL INFORMATION

Supplemental Information includes Supplemental Experimental Procedures, five figures, and seven tables and can be found with this article online at https://doi.org/10.1016/j.celrep.2018.06.027.

ACKNOWLEDGMENTS

We thank Austin Smith, Allan Bradley, Pentao Liu, and Meng Amy Li for discussion and their scientific advice. We also thank Bee Ling Ng, Jennifer Graham, Christopher Hall, and Sam Thompson for flow cytometry analysis and cell sorting and the Sanger Institute DNA pipeline for Illumina sequencing. This work was supported by the Wellcome Trust (WT206194).

AUTHOR CONTRIBUTIONS

M.L. and K.Y. conceived this study. M.L. performed the CRISPR-KO screens, validation of the screen hits, and molecular analysis. J.S.L.Y. performed the kinase assay and qRT-PCR analysis. S.H.O. performed bioinfomatic analysis. K.T. and H.K.-Y. assisted M.L. with experiments. K.Y. and J.S.L.Y. wrote the manuscript, with input from all authors.

DECLARATION OF INTERESTS

The authors declare no competing interests.

Received: January 15, 2018
Revised: April 26, 2018
Accepted: June 6, 2018
Published: July 10, 2018

REFERENCES

Adli, M. (2018). The CRISPR tool kit for genome editing and beyond. Nat. Commun. *9*, 1911.

Bar-Peled, L., Chantranupong, L., Cherniack, A.D., Chen, W.W., Ottina, K.A., Grabiner, B.C., Spear, E.D., Carter, S.L., Meyerson, M., and Sabatini, D.M. (2013). A tumor suppressor complex with GAP activity for the Rag GTPases that signal amino acid sufficiency to mTORC1. Science *340*, 1100–1106.

Batista, P.J., Molinie, B., Wang, J., Qu, K., Zhang, J., Li, L., Bouley, D.M., Lujan, E., Haddad, B., Daneshvar, K., et al. (2014). m(6)A RNA modification controls cell fate transition in mammalian embryonic stem cells. Cell Stem Cell *15*, 707–719.

Bechard, M., and Dalton, S. (2009). Subcellular localization of glycogen synthase kinase 3beta controls embryonic stem cell self-renewal. Mol. Cell. Biol. *29*, 2092–2104.

Betschinger, J., Nichols, J., Dietmann, S., Corrin, P.D., Paddison, P.J., and Smith, A. (2013). Exit from pluripotency is gated by intracellular redistribution of the bHLH transcription factor Tfe3. Cell *153*, 335–347.

Bulut-Karslioglu, A., Biechele, S., Jin, H., Macrae, T.A., Hejna, M., Gertsenstein, M., Song, J.S., and Ramalho-Santos, M. (2016). Inhibition of mTOR induces a paused pluripotent state. Nature *540*, 119–123.

Burr, M.L., Sparbier, C.E., Chan, Y.C., Williamson, J.C., Woods, K., Beavis, P.A., Lam, E.Y.N., Henderson, M.A., Bell, C.C., Stolzenburg, S., et al. (2017). CMTM6 maintains the expression of PD-L1 and regulates anti-tumour immunity. Nature *549*, 101–105.

Chambers, I., Silva, J., Colby, D., Nichols, J., Nijmeijer, B., Robertson, M., Vrana, J., Jones, K., Grotewold, L., and Smith, A. (2007). Nanog safeguards pluripotency and mediates germline development. Nature *450*, 1230–1234.

Cole, M.F., Johnstone, S.E., Newman, J.J., Kagey, M.H., and Young, R.A. (2008). Tcf3 is an integral component of the core regulatory circuitry of embryonic stem cells. Genes Dev. *22*, 746–755.

Cruz-Molina, S., Respuela, P., Tebartz, C., Kolovos, P., Nikolic, M., Fueyo, R., van Ijcken, W.F.J., Grosveld, F., Frommolt, P., Bazzi, H., et al. (2017). PRC2 facilitates the regulatory topology required for poised enhancer function during pluripotent stem cell differentiation. Cell Stem Cell *20*, 689–705.

Dabora, S.L., Jozwiak, S., Franz, D.N., Roberts, P.S., Nieto, A., Chung, J., Choy, Y.S., Reeve, M.P., Thiele, E., Egelhoff, J.C., et al. (2001). Mutational analysis in a cohort of 224 tuberous sclerosis patients indicates increased severity of TSC2, compared with TSC1, disease in multiple organs. Am. J. Hum. Genet. *68*, 64–80.

Evers, B., Jastrzebski, K., Heijmans, J.P., Grernrum, W., Beijersbergen, R.L., and Bernards, R. (2016). CRISPR knockout screening outperforms shRNA and CRISPRi in identifying essential genes. Nat. Biotechnol. *34*, 631–633.

Forsburg, S.L. (2001). The art and design of genetic screens: yeast. Nat. Rev. Genet. *2*, 659–668.

Geula, S., Moshitch-Moshkovitz, S., Dominissini, D., Mansour, A.A., Kol, N., Salmon-Divon, M., Hershkovitz, V., Peer, E., Mor, N., Manor, Y.S., et al. (2015). Stem cells. m6A mRNA methylation facilitates resolution of naïve pluripotency toward differentiation. Science *347*, 1002–1006.

Guertin, D.A., Stevens, D.M., Thoreen, C.C., Burds, A.A., Kalaany, N.Y., Moffat, J., Brown, M., Fitzgerald, K.J., and Sabatini, D.M. (2006). Ablation in mice of the mTORC components raptor, rictor, or mLST8 reveals that mTORC2 is required for signaling to Akt-FOXO and PKCalpha, but not S6K1. Dev. Cell *11*, 859–871.

Guo, G., Huang, Y., Humphreys, P., Wang, X., and Smith, A. (2011). A PiggyBac-based recessive screening method to identify pluripotency regulators. PLoS ONE *6*, e18189.

Hackett, J.A., and Surani, M.A. (2014). Regulatory principles of pluripotency: from the ground state up. Cell Stem Cell *15*, 416–430.

Hart, T., Chandrashekhar, M., Aregger, M., Steinhart, Z., Brown, K.R., MacLeod, G., Mis, M., Zimmermann, M., Fradet-Turcotte, A., Sun, S., et al. (2015). High-resolution CRISPR screens reveal fitness genes and genotype-specific cancer liabilities. Cell *163*, 1515–1526.

Huang, J., Dibble, C.C., Matsuzaki, M., and Manning, B.D. (2008). The TSC1-TSC2 complex is required for proper activation of mTOR complex 2. Mol. Cell. Biol. *28*, 4104–4115.

Inoki, K., Li, Y., Xu, T., and Guan, K.L. (2003). Rheb GTPase is a direct target of TSC2 GAP activity and regulates mTOR signaling. Genes Dev. *17*, 1829–1834.

Jinek, M., Chylinski, K., Fonfara, I., Hauer, M., Doudna, J.A., and Charpentier, E. (2012). A programmable dual-RNA-guided DNA endonuclease in adaptive bacterial immunity. Science *337*, 816–821.

Jinek, M., Jiang, F., Taylor, D.W., Sternberg, S.H., Kaya, E., Ma, E., Anders, C., Hauer, M., Zhou, K., Lin, S., et al. (2014). Structures of Cas9 endonucleases reveal RNA-mediated conformational activation. Science *343*, 1247997.

Jorgensen, E.M., and Mango, S.E. (2002). The art and design of genetic screens: Caenorhabditis elegans. Nat. Rev. Genet. *3*, 356–369.

Kaji, K., Caballero, I.M., MacLeod, R., Nichols, J., Wilson, V.A., and Hendrich, B. (2006). The NuRD component Mbd3 is required for pluripotency of embryonic stem cells. Nat. Cell Biol. *8*, 285–292.

Kanellopoulou, C., Muljo, S.A., Kung, A.L., Ganesan, S., Drapkin, R., Jenuwein, T., Livingston, D.M., and Rajewsky, K. (2005). Dicer-deficient mouse embryonic stem cells are defective in differentiation and centromeric silencing. Genes Dev. *19*, 489–501.

Karwacki-Neisius, V., Göke, J., Osorno, R., Halbritter, F., Ng, J.H., Weiße, A.Y., Wong, F.C., Gagliardi, A., Mullin, N.P., Festuccia, N., et al. (2013). Reduced Oct4 expression directs a robust pluripotent state with distinct signaling activity and increased enhancer occupancy by Oct4 and Nanog. Cell Stem Cell *12*, 531–545.

Koike-Yusa, H., Li, Y., Tan, E.P., Velasco-Herrera, Mdel.C., and Yusa, K. (2014). Genome-wide recessive genetic screening in mammalian cells with a lentiviral CRISPR-guide RNA library. Nat. Biotechnol. *32*, 267–273.

Kunath, T., Saba-El-Leil, M.K., Almousailleakh, M., Wray, J., Meloche, S., and Smith, A. (2007). FGF stimulation of the Erk1/2 signalling cascade triggers transition of pluripotent embryonic stem cells from self-renewal to lineage commitment. Development *134*, 2895–2902.

Leeb, M., Dietmann, S., Paramor, M., Niwa, H., and Smith, A. (2014). Genetic exploration of the exit from self-renewal using haploid embryonic stem cells. Cell Stem Cell *14*, 385–393.

Leitch, H.G., McEwen, K.R., Turp, A., Encheva, V., Carroll, T., Grabole, N., Mansfield, W., Nashun, B., Knezovich, J.G., Smith, A., et al. (2013). Naive pluripotency is associated with global DNA hypomethylation. Nat. Struct. Mol. Biol. *20*, 311–316.

Li, W., Xu, H., Xiao, T., Cong, L., Love, M.I., Zhang, F., Irizarry, R.A., Liu, J.S., Brown, M., and Liu, X.S. (2014). MAGeCK enables robust identification of essential genes from genome-scale CRISPR/Cas9 knockout screens. Genome Biol. *15*, 554.

Li, T., Shi, Y., Wang, P., Guachalla, L.M., Sun, B., Joerss, T., Chen, Y.S., Groth, M., Krueger, A., Platzer, M., et al. (2015). Smg6/Est1 licenses embryonic stem cell differentiation via nonsense-mediated mRNA decay. EMBO J. *34*, 1630–1647.

Makarova, K.S., Wolf, Y.I., Alkhnbashi, O.S., Costa, F., Shah, S.A., Saunders, S.J., Barrangou, R., Brouns, S.J., Charpentier, E., Haft, D.H., et al. (2015). An updated evolutionary classification of CRISPR-Cas systems. Nat. Rev. Microbiol. *13*, 722–736.

Marks, H., Kalkan, T., Menafra, R., Denissov, S., Jones, K., Hofemeister, H., Nichols, J., Kranz, A., Stewart, A.F., Smith, A., and Stunnenberg, H.G. (2012). The transcriptional and epigenomic foundations of ground state pluripotency. Cell *149*, 590–604.

Martello, G., Sugimoto, T., Diamanti, E., Joshi, A., Hannah, R., Ohtsuka, S., Göttgens, B., Niwa, H., and Smith, A. (2012). Esrrb is a pivotal target of the Gsk3/Tcf3 axis regulating embryonic stem cell self-renewal. Cell Stem Cell *11*, 491–504.

Michlits, G., Hubmann, M., Wu, S.H., Vainorius, G., Budusan, E., Zhuk, S., Burkard, T.R., Novatchkova, M., Aichinger, M., Lu, Y., et al. (2017). CRISPR-UMI: single-cell lineage tracing of pooled CRISPR-Cas9 screens. Nat. Methods *14*, 1191–1197.

Murakami, M., Ichisaka, T., Maeda, M., Oshiro, N., Hara, K., Edenhofer, F., Kiyama, H., Yonezawa, K., and Yamanaka, S. (2004). mTOR is essential for growth and proliferation in early mouse embryos and embryonic stem cells. Mol. Cell. Biol. *24*, 6710–6718.

Nishimasu, H., Ran, F.A., Hsu, P.D., Konermann, S., Shehata, S.I., Dohmae, N., Ishitani, R., Zhang, F., and Nureki, O. (2014). Crystal structure of Cas9 in complex with guide RNA and target DNA. Cell *156*, 935–949.

Niwa, H., Miyazaki, J., and Smith, A.G. (2000). Quantitative expression of Oct-3/4 defines differentiation, dedifferentiation or self-renewal of ES cells. Nat. Genet. *24*, 372–376.

Ohtsuka, S., Nakai-Futatsugi, Y., and Niwa, H. (2015). LIF signal in mouse embryonic stem cells. JAK-STAT *4*, e1086520.

Ong, S.H., Li, Y., Koike-Yusa, H., and Yusa, K. (2017). Optimised metrics for CRISPR-KO screens with second-generation gRNA libraries. Sci. Rep. *7*, 7384.

Ornitz, D.M. (2000). FGFs, heparan sulfate and FGFRs: complex interactions essential for development. BioEssays *22*, 108–112.

Parnas, O., Jovanovic, M., Eisenhaure, T.M., Herbst, R.H., Dixit, A., Ye, C.J., Przybylski, D., Platt, R.J., Tirosh, I., Sanjana, N.E., et al. (2015). A genome-wide CRISPR screen in primary immune cells to dissect regulatory networks. Cell *162*, 675–686.

Pereira, L., Yi, F., and Merrill, B.J. (2006). Repression of Nanog gene transcription by Tcf3 limits embryonic stem cell self-renewal. Mol. Cell. Biol. *26*, 7479–7491.

Ricos, M.G., Hodgson, B.L., Pippucci, T., Saidin, A., Ong, Y.S., Heron, S.E., Licchetta, L., Bisulli, F., Bayly, M.A., Hughes, J., et al.; Epilepsy Electroclinical Study Group (2016). Mutations in the mammalian target of rapamycin pathway regulators NPRL2 and NPRL3 cause focal epilepsy. Ann. Neurol. *79*, 120–131.

Sato, N., Meijer, L., Skaltsounis, L., Greengard, P., and Brivanlou, A.H. (2004). Maintenance of pluripotency in human and mouse embryonic stem cells through activation of Wnt signaling by a pharmacological GSK-3-specific inhibitor. Nat. Med. *10*, 55–63.

Saxton, R.A., and Sabatini, D.M. (2017). mTOR signaling in growth, metabolism, and disease. Cell *169*, 361–371.

Shalem, O., Sanjana, N.E., Hartenian, E., Shi, X., Scott, D.A., Mikkelson, T., Heckl, D., Ebert, B.L., Root, D.E., Doench, J.G., and Zhang, F. (2014). Genome-scale CRISPR-Cas9 knockout screening in human cells. Science *343*, 84–87.

Sinkkonen, L., Hugenschmidt, T., Berninger, P., Gaidatzis, D., Mohn, F., Artus-Revel, C.G., Zavolan, M., Svoboda, P., and Filipowicz, W. (2008). MicroRNAs control *de novo* DNA methylation through regulation of transcriptional repressors in mouse embryonic stem cells. Nat. Struct. Mol. Biol. *15*, 259–267.

Smith, A. (2017). Formative pluripotency: the executive phase in a developmental continuum. Development *144*, 365–373.

St Johnston, D. (2002). The art and design of genetic screens: *Drosophila melanogaster*. Nat. Rev. Genet. *3*, 176–188.

Sternberg, S.H., Redding, S., Jinek, M., Greene, E.C., and Doudna, J.A. (2014). DNA interrogation by the CRISPR RNA-guided endonuclease Cas9. Nature *507*, 62–67.

Takashima, Y., Guo, G., Loos, R., Nichols, J., Ficz, G., Krueger, F., Oxley, D., Santos, F., Clarke, J., Mansfield, W., et al. (2014). Resetting transcription factor control circuitry toward ground-state pluripotency in human. Cell *158*, 1254–1269.

Theunissen, T.W., Powell, B.E., Wang, H., Mitalipova, M., Faddah, D.A., Reddy, J., Fan, Z.P., Maetzel, D., Ganz, K., Shi, L., et al. (2014). Systematic identification of culture conditions for induction and maintenance of naive human pluripotency. Cell Stem Cell *15*, 523.

Tzelepis, K., Koike-Yusa, H., De Braekeleer, E., Li, Y., Metzakopian, E., Dovey, O.M., Mupo, A., Grinkevich, V., Li, M., Mazan, M., et al. (2016). A CRISPR dropout screen identifies genetic vulnerabilities and therapeutic targets in acute myeloid leukemia. Cell Rep. *17*, 1193–1205.

Wang, Z., Oron, E., Nelson, B., Razis, S., and Ivanova, N. (2012). Distinct lineage specification roles for NANOG, OCT4, and SOX2 in human embryonic stem cells. Cell Stem Cell *10*, 440–454.

Wang, T., Wei, J.J., Sabatini, D.M., and Lander, E.S. (2014). Genetic screens in human cells using the CRISPR-Cas9 system. Science *343*, 80–84.

Wang, T., Yu, H., Hughes, N.W., Liu, B., Kendirli, A., Klein, K., Chen, W.W., Lander, E.S., and Sabatini, D.M. (2017). Gene essentiality profiling reveals gene networks and synthetic lethal interactions with oncogenic Ras. Cell *168*, 890–903.

Watanabe, S., Umehara, H., Murayama, K., Okabe, M., Kimura, T., and Nakano, T. (2006). Activation of Akt signaling is sufficient to maintain pluripotency in mouse and primate embryonic stem cells. Oncogene *25*, 2697–2707.

Whyte, W.A., Bilodeau, S., Orlando, D.A., Hoke, H.A., Frampton, G.M., Foster, C.T., Cowley, S.M., and Young, R.A. (2012). Enhancer decommissioning by LSD1 during embryonic stem cell differentiation. Nature *482*, 221–225.

Wolfson, R.L., and Sabatini, D.M. (2017). The dawn of the age of amino acid sensors for the mTORC1 pathway. Cell Metab. *26*, 301–309.

Wolfson, R.L., Chantranupong, L., Saxton, R.A., Shen, K., Scaria, S.M., Cantor, J.R., and Sabatini, D.M. (2016). Sestrin2 is a leucine sensor for the mTORC1 pathway. Science *351*, 43–48.

Wolfson, R.L., Chantranupong, L., Wyant, G.A., Gu, X., Orozco, J.M., Shen, K., Condon, K.J., Petri, S., Kedir, J., Scaria, S.M., et al. (2017). KICSTOR recruits GATOR1 to the lysosome and is necessary for nutrients to regulate mTORC1. Nature *543*, 438–442.

Wray, J., Kalkan, T., Gomez-Lopez, S., Eckardt, D., Cook, A., Kemler, R., and Smith, A. (2011). Inhibition of glycogen synthase kinase-3 alleviates Tcf3 repression of the pluripotency network and increases embryonic stem cell resistance to differentiation. Nat. Cell Biol. *13*, 838–845.

Yang, S.H., Kalkan, T., Morissroe, C., Marks, H., Stunnenberg, H., Smith, A., and Sharrocks, A.D. (2014). Otx2 and Oct4 drive early enhancer activation during embryonic stem cell transition from naive pluripotency. Cell Rep. *7*, 1968–1981.

Yi, F., Pereira, L., Hoffman, J.A., Shy, B.R., Yuen, C.M., Liu, D.R., and Merrill, B.J. (2011). Opposing effects of Tcf3 and Tcf1 control Wnt stimulation of embryonic stem cell self-renewal. Nat. Cell Biol. *13*, 762–770.

Yilmaz, A., Peretz, M., Aharony, A., Sagi, I., and Benvenisty, N. (2018). Defining essential genes for human pluripotent stem cells by CRISPR-Cas9 screening in haploid cells. Nat. Cell Biol. *20*, 610–619.

Ying, Q.L., Wray, J., Nichols, J., Batlle-Morera, L., Doble, B., Woodgett, J., Cohen, P., and Smith, A. (2008). The ground state of embryonic stem cell self-renewal. Nature *453*, 519–523.

Zhang, H., Cicchetti, G., Onda, H., Koon, H.B., Asrican, K., Bajraszewski, N., Vazquez, F., Carpenter, C.L., and Kwiatkowski, D.J. (2003). Loss of Tsc1/Tsc2 activates mTOR and disrupts PI3K-Akt signaling through downregulation of PDGFR. J. Clin. Invest. *112*, 1223–1233.

Zhang, H.H., Lipovsky, A.I., Dibble, C.C., Sahin, M., and Manning, B.D. (2006). S6K1 regulates GSK3 under conditions of mTOR-dependent feedback inhibition of Akt. Mol. Cell *24*, 185–197.

Zhou, W., Choi, M., Margineantu, D., Margaretha, L., Hesson, J., Cavanaugh, C., Blau, C.A., Horwitz, M.S., Hockenbery, D., Ware, C., and Ruohola-Baker, H. (2012). HIF1α induced switch from bivalent to exclusively glycolytic metabolism during ESC-to-EpiSC/hESC transition. EMBO J. *31*, 2103–2116.

Cell Reports, Volume *24*

Supplemental Information

Genome-wide CRISPR-KO Screen Uncovers mTORC1-Mediated Gsk3 Regulation in Naive Pluripotency Maintenance and Dissolution

Meng Li, Jason S.L. Yu, Katarzyna Tilgner, Swee Hoe Ong, Hiroko Koike-Yusa, and Kosuke Yusa

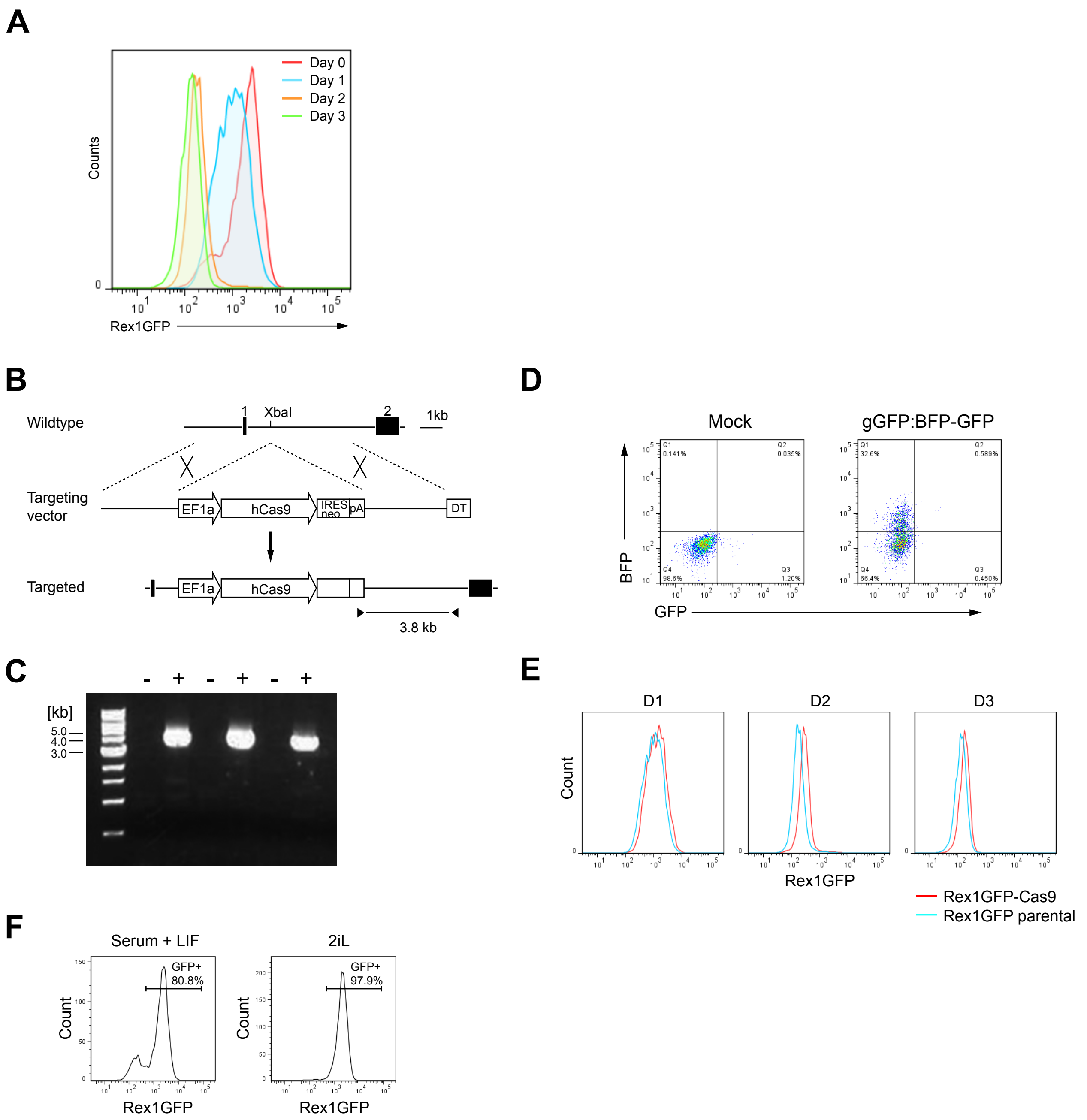

Figure S1. Generation and characterisation of CRISPR-ready Rex1GFP mESCs, Related to Figures 1 and 2.

A. Rex1GFP profile during differentiation in N2B27. **B**. Knock-in strategy of the Cas9 expression cassette at the mouse *Rosa26* locus. The targeting vector used was described in (Tzelepis et al., 2016). **C**. Detection of targeted insertion by long-range PCR. **D**. Cas9 functional assay in Cas9-expressing cells. Two days after transduction, cells were differentiated in N2B27 to remove GFP signal derived from the *Rex1* locus. **E**. Comparison of Rex1GFP profiles with or without Cas9 expression. **F**. Rex1GFP profiles in cells cultured in SL or 2iL condition.

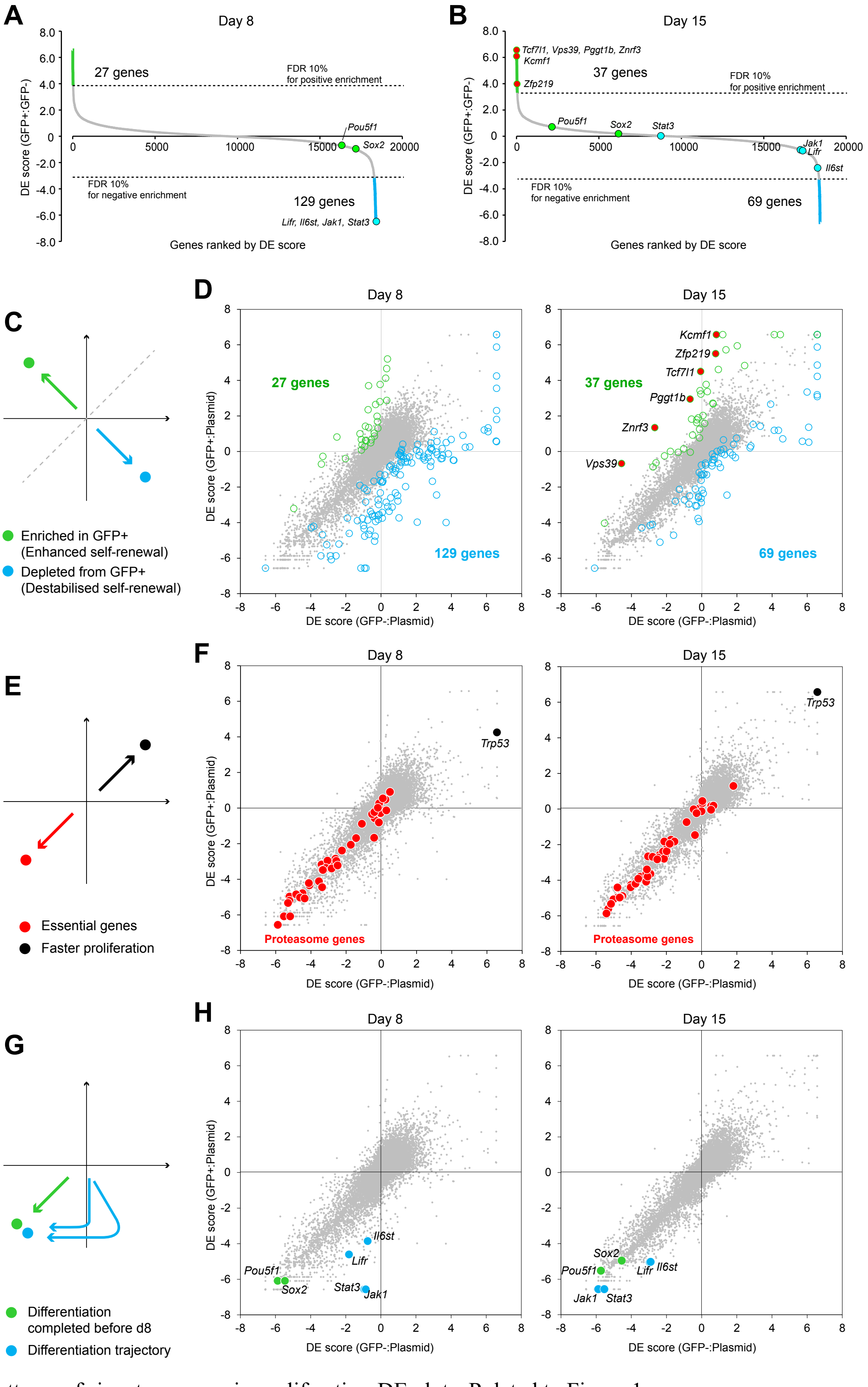

Figure S2. Patterns of signature genes in proliferation-DE plots, Related to Figure 1

A-B. Genes ranked by DE score derived from MAGeCK analysis between GFP+ and GFP- populations on d8 (A) and d15 (B) as shown in Figure 1B and C, respectively. **C-D**. Genes significantly enriched in (green) or depleted from (blue) the GFP+ population (shown in A and B with the same colour) are highlighted in the DE plots. DE scores in these plots were derived from MAGeCK analysis between plasmid counts and GFP- (x axis) or GFP+ (y axis) population; hence, these DE scores represent proliferation phenotype in each population. Genes validated in Figure 1D are highlighted in D (right panel). **E-F**. Genes commonly essential (e.g. Proteasome genes) or giving growth advantage (e.g. *Trp53*) are located in the bottom-left or top-right quadrant, respectively, along the +45° diagonal line. **G-H**. Trajectory of known pluripotency maintenance genes. *Pou5f1* and *Sox2* show rapid differentiation after knockout and depletion from the entire population; therefore, these genes were detected as essential to both population (the bottom-left corner on the diagonal line). On the other hand, genes in the LIF-Stat3 pathways showed depletion from GFP+ population on d8 and then from the entire population by d15.

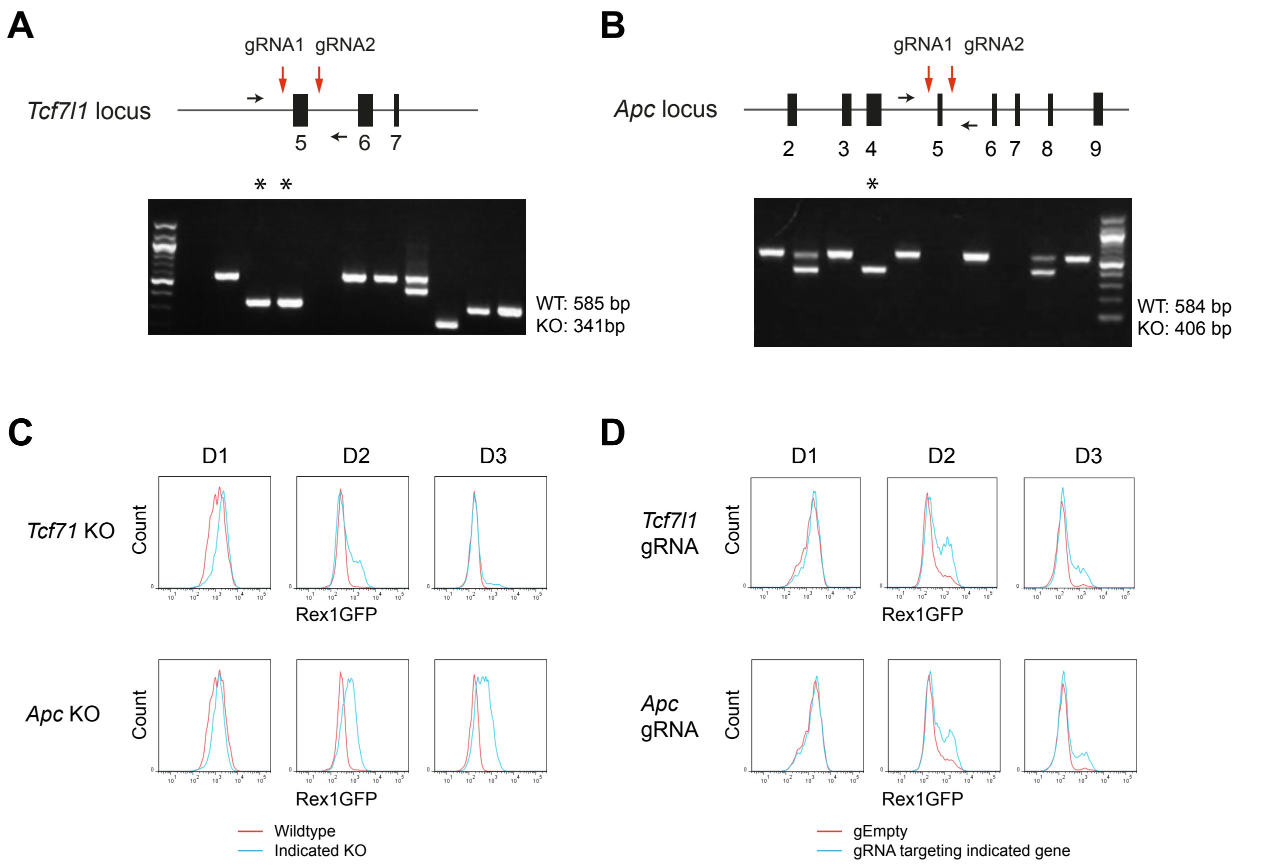

Figure S3. Generation of Tcf7l1 and Apc KO mESCs and their phenotype on differentiation, Related to Figure 2.
A-B. CRISPR-based gene KO and genotyping strategy of *Tcf7l1* (A) and *Apc* (B). **C**. Rex1GFP profiles of indicated KO mESCs between d1 and d3 differentiation. **D**. Rex1GFP profiles of bulk KO mESCs generated by lentiviral gRNA expression. Astariscs indicate knockout clones.

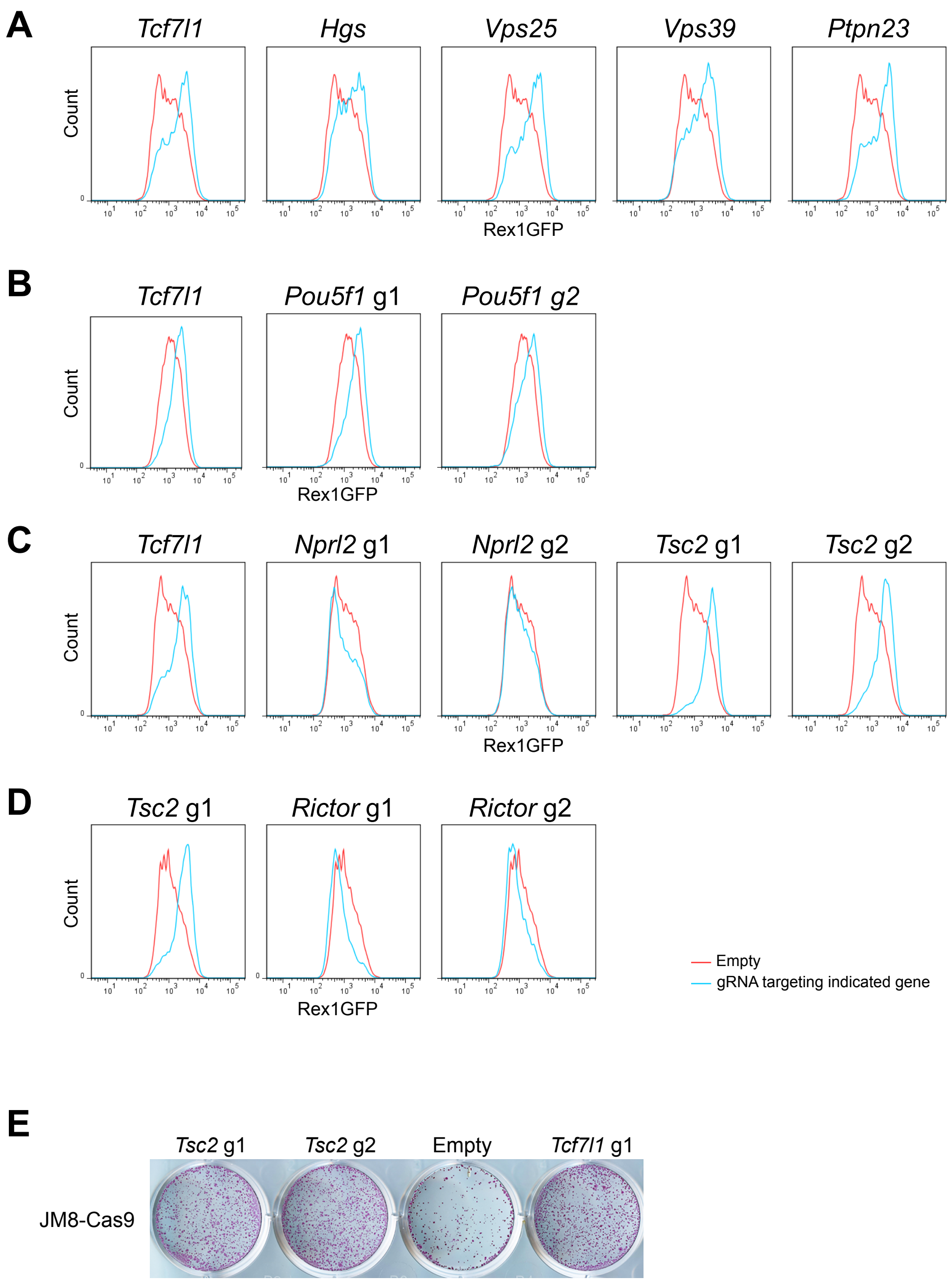

Figure S4. Validation of differentiation screen hits, Related to Figures 3,4 and 5.

A-D. Rex1-Cas9 mESCs were transduced with lentiviral expressing indicated gRNA. 5-7 days post transduction, BFP+/GFP+ cells were collected by sorting and differentiated in Ndiff227 differentiation medium for 27 h. Genes involved in vesicle trafficking (A), *Pou5f1* (B), negative regulators of mTORC1 (C) and mTORC2 (D) were disrupted by lentiviral gRNA expression. *Tcf7l1* (A-C) and *Tsc2* (D) were used as positive controls. **E**. *Tsc2* KO phenotype was also confirmed in a different mESC line. JM8-Cas9 cells were transduced with lentivirus expressing *Tsc2* gRNA. Seven days post transduction, BFP+ cells were collected by sorting, differentiated for 24h and then re-seeded in 2iL medium. Resulting colonies were stained with alkaline phosphatase kit.

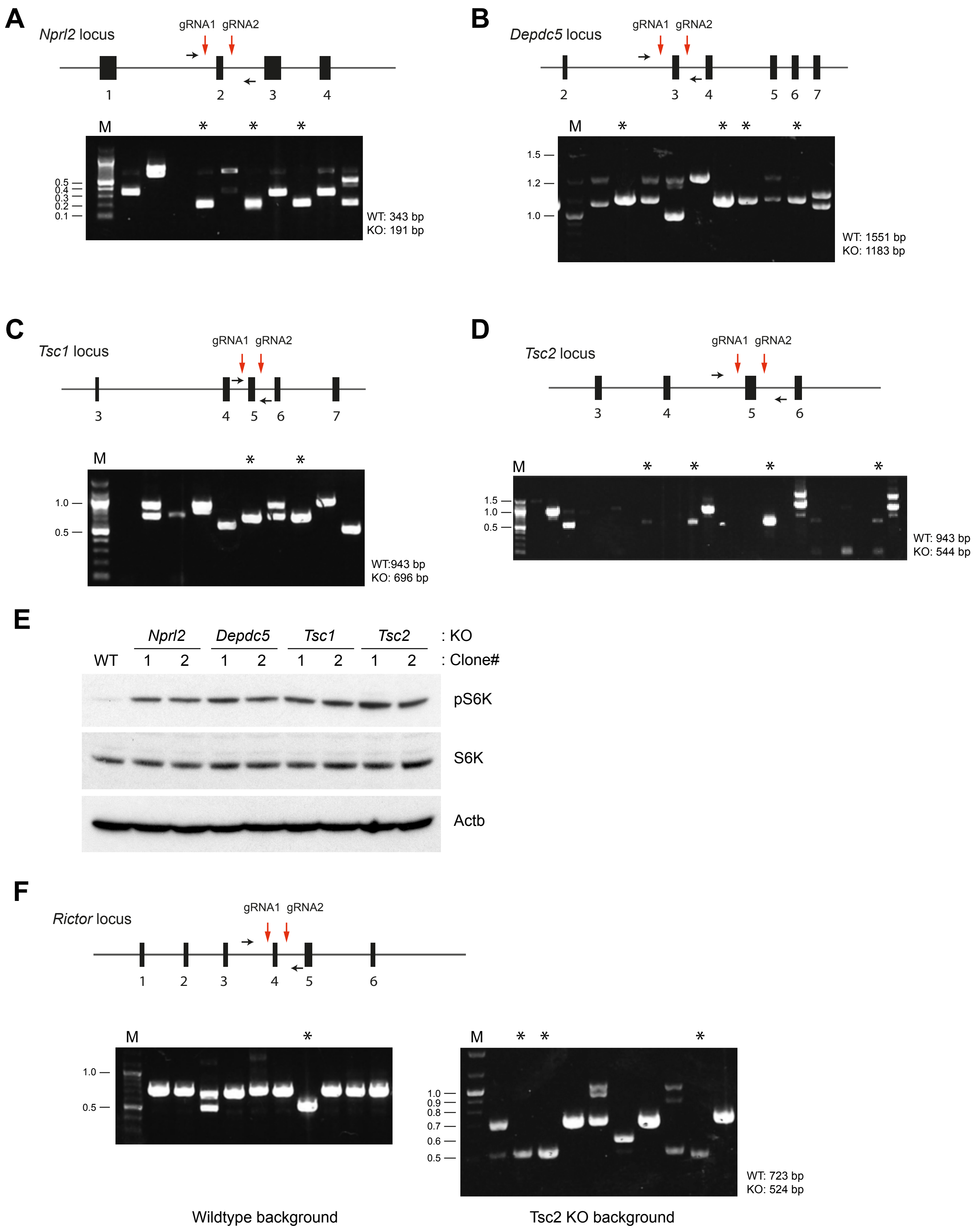

Figure S5. Generation of *Nprl2*, *Depdc5*, *Tsc1*, *Tsc2* and *Rictor* KO mESCs, Related to Figures 4 and 5.

A-D. CRISPR-based gene KO and genotyping strategy of *Nprl2* (A), *Depdc5* (B), *Tsc1* (C) and *Tsc2* (D). **E**. Western blots showing mTORC1 activation as upregulated phospho-S6K. **F**. CRISPR-based gene KO and genotyping strategy of Rictor. Asterisks indicate knockout clones.

Supplemental Experimental Procedures

Cell culture

A mESC line, Rex1dGFP-IRES-Bsd (Wray et al., 2011), was a kind gift from Austin Smith. mESCs were cultured on mitomycin C-treated mouse embryonic fibroblasts (MEFs) in SL (Serum+LIF): Knockout DMEM (Thermo Fisher) supplemented with 15% foetal bovine serum (Thermo Fisher), 1 % GlutaMax (Thermo Fisher), 1 % nonessential amino acids (Thermo Fisher), 0.1 mM 2-mercaptoethanol (Sigma) and 1,000 U ml^{-1} leukaemia inhibitory factor (LIF; Millipore). Where indicated, mESCs were cultured on gelatin-coated plates in 2iL medium: NDiff227 (Takara) supplemented with 1% knockout serum replacement (Thermo Fisher), 5 % BSA (Thermo Fisher) , 1 % nonessential amino acids, 0.1 mM 2-mercaptoethanol, 1,000 U ml^{-1} LIF, 1.0 µM PD0325901 (Selleck) and 3.0 µM CHIR90021 (Selleck). Differentiation was induced in NDif227 medium supplemented with 1% knockout serum replacement (Thermo Fisher), 5 % BSA (Thermo Fisher) , 1 % nonessential amino acids and 0.1 mM 2-mercaptoethanol. MEFs were cultured in DMEM (Thermo Fisher) supplemented with 15% foetal bovine serum, 1 % GlutaMax, 1 % nonessential amino acids, 0.1 mM 2-mercaptoethanol and mitotically inactivated by mitomycin C (Sigma) treatment at 15 µg ml^{-1} for 2.5 h before use. 293FT (Thermo Fisher) cells were cultured in DMEM (Thermo Fisher) containing 10% FBS and 1% GlutaMax. All cell lines used in this study are negative for mycoplasma contamination.

Generation of Rex1-Cas9 cell lines

The human elongation factor 1α promoter-driven Cas9 expression cassette was knocked into the *Rosa26* locus as described previously (Tzelepis et al., 2016). Cas9 expression was tested using a Cas9 reporter system with pKLV2-U6gRNA5(gBFP)-PGKmCherry2ABFP-W (Tzelepis et al., 2016). A selected cell line ($Rex1^{GFPd2\text{-}IRES\text{-}Bsd/+}$;$Rosa26^{EF1a\text{-}hCas9\text{-}IRES\text{-}neo/+}$) was designated as Rex1-Cas9 mESCs.

CRISPR-KO screen on self-renewal

Thirty two million Rex1-Cas9 cells were transduced with the mouse v2 CRISPR library (Tzelepis et al., 2016) at a pre-defined condition that gave rise to 30% transduction efficiency as described before (Koike-Yusa et al., 2014). On day2, approximately 10 million cells double positive for GFP and BFP were collected by cell sorting. Of these, 5 million cells were further cultured on mitomycin C-treated MEFs in SL and the other 5 million cells were cultured in 2iL medium. Thirty million cells were plated at each passage to maintain 300x representation of each gRNA. On d8 and d15 after transduction, cells from the SL condition were sorted according to their GFP expression and genomic DNA was isolated from each fraction using DNeasy Blood and Tissue kit (Qiagen). Cells from the 2iL condition were directly subjected to genomic DNA isolation using Blood and Cell Culture DNA Maxi kit (Qiagen).

CRISPR-KO screen on exit from pluripotency

Transduction and cell sorting on day 2 were performed as described above. Sorted cells were plated on gelatin-coated plate in 2iL medium and cultured for an additional 4 days. On day 6, cells were trypsinised and a total of 45 million cells were plated on eight 15-cm dishes (10,000 cells cm^{-1}) in NDiff227 differentiation medium to induce differentiation. After 2 days of differentiation, cells were trypsinised and 20 million cells were kept as a pre-sort control. The remaining cells were used for cell sorting and approximately 3 million GFP+ cells (top 2-3 %) were collected. Genomic DNA from the pre-sort and GFP+ fraction were isolated using Blood and Cell Culture DNA Maxi kit (Qiagen) and DNeasy Blood and Tissue kit, respectively. This screen was performed in 4 biological replicates.

gRNA sequencing

gRNA amplification from genomic DNA and Illumina sequencing were performed as described previously (Koike-Yusa et al., 2014) with minor modifications. As the total amount of genomic DNA from the sorted populations (2-3 million cells) is limited, all available genomic DNA (typically 5 µg) was used in the first-round PCR at 200 ng per reaction in 24 reactions to maximise the coverage.

CRISPR-KO screen data analysis

Data analysis was performed essentially as described previously. Briefly, read counts were generated using an in-house gRNA counting script and then statistical analysis was performed using MAGeCK (Li et al., 2014). DE scores were calculated from gene-level significance returned by MAGeCK with the following formula:

DE score = $\log_{10}$(gene-level depletion P value) – $\log_{10}$(gene-level enrichment P value).

GSEA was performed using the GSEA pre-ranked module on GenePattern (https://genepattern.broadinstitute.org/). A gene rank based on DE scores was used as a rank list.

Generation of KO cell lines by exon deletion

Genes listed in Supplementary Table 3 were knocked out by deleting a 'critical' exon, which would most likely induce nonsense-mediated decay when deleted. Two gRNAs, one each in the upstream and downstream intron, were designed using WGE (Supplementary Table 3) and cloned into pKLV2-U6gRNA5(BbsI)-PGKpuroBFP-W (Tzelepis et al., 2016). Rex1-Cas9 cells were co-transfected with a pair of gRNAs using LipofectamineLTX as described previously (Koike-Yusa et al., 2014). BFP-positive cells were then collected 3 days after transfection by cell sorting (BD Influx or Mo-Flo) and plated on 10-cm dishes at a clonal density. Colonies were subsequently picked and genotyped using primers listed in Supplementary Table 4 as described before (Koike-Yusa et al., 2014). Clones that carried expected deletion on both alleles were further expanded (Supplementary Fgis. 3, 5).

Generation of KO mESCs by lentiviral CRISPR

Genes listed in Supplementary Table 5 were knocked out by transducing Rex1-Cas9 cells with gRNA-expressing lentivirus. gRNAs were selected from our v2 mouse library based on the degree of phenotype, and cloned into pKLV2-U6gRNA5(BbsI)-PGKpuroBFP-W. Lentiviral transduction was performed as described previously (Koike-Yusa et al., 2014). Cells were further cultured and used for assays at indicated time points.

Self-renewal assay

GFP+ or GFP- cells were sorted from parental cells and *Tcf7l1*, *Nprl2* or *Depdc5* KO mESCs and cultured either on feeder layers in M15L or on gelatin-coated dishes in 2iL. On indicated days, cells were harvested and analysed by flow cytometry (BD Fortessa II).

Differentiation assay

3.8 x 10^5 Rex1GFP$^+$ cells were collected by cell sorting from wildtype or KO cell lines that had been maintained in SL condition and plated on a well of gelatin-coated 12-well plates in NDiff227 differentiation medium. When gene knockout was conducted by lentiviral CRISPR, 3.8 x 10^5 cells double-positive for GFP and BFP were collected. Rex1GFP expression was measured 24-27 h post plating, unless otherwise stated. Where indicated, rapamycin (20 nM) or DMSO was added when sorted cells were plated for differentiation. For replating assay, cells were differentiated as described above for 28 h, trypsinised, counted and replated into 2iL medium at 20,000 cells per well of 12-well plate. After 6-8 days, cells were stained with alkaline phosphatase staining kit (Sigma) and colonies were counted.

RNA-seq and data analysis

GFP+ cells were collected by cell sorting from wildtype, *Nprl2* KO and *Tsc2* KO mESCs and used for total RNA isolation with RNeasy mini kit (Qiagen). Total RNA was then converted into Illumina libraries using KAPA Stranded mRNA-Seq Kit (KAPA Biosystems) and sequenced by paired-end 75-bp sequencing on Illumina HiSeq2500. Transcript counts were generated using Kalisto (Bray et al., 2016) and differential expression was analysed using

DEseq2 (Love et al., 2014). Gene ontology (GO) analysis was performed using GO_Biological Processes on the GSEA platform (http://software.broadinstitute.org/gsea/).

Western blot

mESCs were directly lysed on tissue culture plates by adding RIPA buffer (Sigma) supplemented with phosphatase inhibitor (Sigma, 1:100) and protease inhibitor cocktails (Sigma, 1:1000). Lysates were then centrifuged at 15,000 rpm for 15 minutes at 4 °C, and supernatant was collected and stored at -80 °C. Total protein concentration was quantified using Bradford Protein Assay (BioRad) following the manufacturer's protocol. The quantified protein samples were diluted with 4x sample buffer (Thermo Fisher) supplemented with Reducing agent (Thermo Fisher) and heat-denatured at 70 °C for 10 min. The denatured protein samples were loaded at 5-20 µg per lane on a 4-12% gradient Bis-Tris gel (Thermo Fisher) and resolved at 180 V for 1 h in MOPS-SDS running (Thermo Fisher). Dissolved proteins were then transferred onto a PVDF membrane (Millipore) at 90 V for 1 h at 4 °C by wet transfer. Membranes were blocked for 30 minutes in either 5 % BSA (Sigma) or 5 % milk (Sigma) in TBST buffer (TBS supplemented 0.1 % Tween 20) and then probed in diluted primary antibody overnight at 4 °C. The membranes were washed three times in TBST and then probed with appropriate horse radish peroxidase-conjugated secondary antibody for 1 h at room temperature. The membranes were washed three times in TBST, incubated with ECL (Roche) for 5 min at room temperature, and exposed onto an X-ray film.

Kinase assay

mESCs were feeder-depleted by brief incubation on gelatinised dishes in SL medium and allowed to plate down on a fresh 10 cm dish overnight. Cells were then lysed and processed as per Akt Activity Assay Kit (Abcam) with modifications. In brief, Akt was immunoprecipitated via overnight incubation with pan-specific Akt antibody followed by 2 h incubation with Protein-A dynabeads (Thermo Fisher) both at 4 °C with agitation. Beads were then washed and incubated with GSK-3α substrate/ATP reaction mix at 30 °C for 2 or 4 h. Reaction was halted by the addition of 4x sample buffer and processed for immunoblotting to analyse the phosphorylation status of the GSK-3α substrate. All antibodies were supplied by the kit and dilutions were used as indicated in the supplied protocol.

RT-qPCR

Rex1GFP+ cells from wildtype, *Nprl2* KO and *Tsc2* KO cells that had been maintained in SL medium were collected by cell sorting and 5.0×10^5 cells were plated to a gelatin-coated 10-cm dish in NDiff227 differentiation medium. Cells were collected immediately after sorting as d0 samples and after differentiation for 1 and 2 days, and used for total RNA isolation. Reverse transcription was performed using SuperScript III (Thermo Fisher) according to the manufacturer's instruction. qPCR was then performed using Agilent Mx3005P qPCR system with Brilliant III Ultra-Fast SYBR Green PCR Mix with Low ROX (Agilent) and primers listed in Supplementary Table 6.

Table S1. DE scores for all MAGeCK analyses in self-renewal and differentiation screens (Excel file), Related to Figures 1-4.

Table S2. Hit gene list (Excel file), Related to Figures 1-3.

Table S3. Representative Reactome/KEGG gene sets positively enriched in the differentiation screen, Related to Figures 2 and 3.

REACTOME/KEGG gene set	Key process	NES	FDR q
REACTOME_RESPIRATORY_ELECTRON_TRANSPORT	Mitochondria	2.61	0
REACTOME_MITOCHONDRIAL_TRNA_AMINOACYLATION	Mitochondria	2.17	0
KEGG_OXIDATIVE_PHOSPHORYLATION	Mitochondria	2.59	0
REACTOME_GLYCOLYSIS	Glucose metabolism	1.66	0.044
REACTOME_MICRORNA_MIRNA_BIOGENESIS	miRNA	1.59	0.065
REACTOME_METABOLISM_OF_MRNA	mRNA	1.98	0
REACTOME_DESTABILIZATION_OF_MRNA_BY_AUF1_HNRNP_D0	mRNA	1.66	0.042
KEGG_RNA_DEGRADATION	mRNA	2.17	0
KEGG_PROTEASOME	Proteasome	1.77	0.008
KEGG_GLYCOSYLPHOSPHATIDYLINOSITOL_GPI_ANCHOR_BIOSYNTHESIS	GPI anchor	1.80	0.005
KEGG_GLYCOSAMINOGLYCAN_BIOSYNTHESIS_HEPARAN_SULFATE	Heparan sulfate	1.87	0.002
REACTOME_FRS2_MEDIATED_CASCADE	FGF-MAPK	1.71	0.032
REACTOME_PROLONGED_ERK_ACTIVATION_EVENTS	FGF-MAPK	1.63	0.058
REACTOME_INSULIN_RECEPTOR_SIGNALLING_CASCADE	PI3K-AKT-mTORC1 -FGF-MAPK	1.53	0.101
REACTOME_SIGNALING_BY_WNT	Wnt	1.82	0.009
REACTOME_ENDOSOMAL_SORTING_COMPLEX_REQUIRED _FOR_TRANSPORT_ESCRT	Endosome-trafficking	1.81	0.009
REACTOME_ER_PHAGOSOME_PATHWAY	Endosome-trafficking	1.70	0.033

Table S4. gRNAs used to generate KO clones, Related to Figures 2,4 and 5.

Gene	gRNA1	gRNA2
Tcf7l1	GCTCCCAAAGAGCGGTGGTG	TGAAAGGAGCCACCGGTGAG
Apc	ACAAGCTAATACATATTGCC	GACAGTGCAGCTTTTAGATT
Nprl2	CCGGACCCAACGTCCACTGA	GTGTGAGGCTTTAGTTGGGT
Depdc5	CACAGGGCACCCCATCATGT	ACAAAACATGCTCGTCTCTA
Tsc1	GGCGACATCAGGCTCAGCAC	GCAGCCATGTGTATGCGGGA
Tsc2	AAAAGGTGCTGCAGTTCACG	TCCAAGCTTAATGCATTAGG
Rictor	TGCTTGTCTATGCACAATTT	TAACAATTTAAGTCCGAGCT

Table S5. Genotyping primers used to generate KO clones, Related to Figures 2,4 and 5.

Gene	Fw primer	Rv primer
Tcf7l1	AGCCATTTTGACGTCTGTCC	CCGAGAGCTCCTGTCAGAAC
Apc	CGTCAGTGCAGTGTTCCTTC	AGTCTGAAGTCAGCCCAGGA
Nprl2	CAAAGTAGACCACTGGGTGGA	AGAAGAAGCTGATTGGCTGC
Depdc5	ACTCTCAGGGAAAAGGCAGA	TGCTTTTGCAAGTCAAGTCG
Tsc1	GGGGATAGGGATAGGGGTCT	ATGAACTGCAGGGTTTCTGG
Tsc2	GACAGGAGGCAAGCAGAAAC	GCTAGAGAAGGGCAGGGAGT
Rictor	ACGGTGGGACAGAAACTCAG	TCAAGCAGTTTCAGTGCCAC

Table S6. gRNAs used for lentiviral CRISPR-mediated gene knockout, Related to Figures 1-3.

Gene	gRNA	Addgene ID
Tcf7l1-g1	TTACGGTGCGCGCTCCACC	105015
Tcf7l1-g2	GCTGTCTTTGGGTCGATCTC	105016
Kcmf1-g1	TGAGTTACGATGAATCGAG	105017
Pggt1b-g1	GGTCAAATCTGAGTCGCTG	105018
Vps39-g1	CTGTGGTGCTTAACGAGGA	105019
Znrf1-g1	TTGTACTCATCGGTTCCAC	105020
Zfp219-g1	ACGGGGCACGCATGATCAA	105021
Apc-g1	GTATTGTTGGGAAATTCCCG	105022
Nelfb-g1	AAGACCCGGCGCCAAGGAG	105023
Nelfb-g2	GGATCTACACGGGACACCG	105024
Nelfcd-g1	GATCAAGCACTTCGACCCT	105025
Nelfcd-g2	TCTGATGCGGGGTACCAAG	105026
Stat3	ATCGCTTACTCTCCGCATC	105027
Hgs-g1	ATCTGCGACCTGATCCGTC	105032
Vps25-g1	CAATCTGAATTGACTCCAC	105033
Ptpn23-g1	CTAAAGCGCATCCTCGCCA	105034
Pou5f1-g1	TCGTATGCGGGCGGACATG	105035
Pou5f1-g2	CTAGTCCCCCAAGTTGGCG	105036
Nprl2-g1	TCGCGAGAACTGTTTGACA	105037
Nprl2-g2	CGGCCAGCTTTTTAACGAT	105038
Tsc2-g1	CTCATACACTCGAGTGGCG	105039
Tsc2-g2	CAATCGCATCCGAATGATA	105040
Rictor-g1	TAGCGCAGCGCTCGCAACC	105041
Rictor-g2	GACATTCAGCAGAGCAACG	105042

Table S7. Primers used for the RT-qPCR assay, Related to Figure 4.

Gene	Fw primer	Rv primer
Zfp42	CCCTCGACAGACTGACCCTAA	TCGGGGCTAATCTCACTTTCAT
Nanog	TCTTCCTGGTCCCCACAGTTT	GCAAGAATAGTTCTCGGGATGAA
Esrrb	GCACCTGGGCTCTAGTTGC	TACAGTCCTCGTAGCTCTTGC
Tbx3	AGATCCGGTTATCCCTGGGAC	CAGCAGCCCCCACTAACTG
Klf4	GTGCCCCGACTAACCGTTG	GTCGTTGAACTCCTCGGTCT
Tfcp2l1	CAGCCCGAACACTACAACCAG	CAGCCGGATTTCATACGACTG
Fgf5	AAGTAGCGCGACGTTTTCTTC	CTGGAAACTGCTATGTTCCGAG
Otx2	TATCTAAAGCAACCGCCTTACG	AAGTCCATACCCGAAGTGGTC

Deposited datasets

1. RNA-seq analyses. Rex1GFP+ population in wildtype, Nprl2 KO and Tsc2 KO mESCs and differentiation timecourse (day 0-3) of wildtype Rex1GFP mESCs. Gene Expression Omnibus: GSE107060

2. CRISPR-KO screen. Raw readcounts and all MAGeCK outputs of self-renewal and differentiation. BioStudies: S-BSST61
